# SeedGerm-VIG: an open and comprehensive pipeline to quantify seed vigor in wheat and other cereal crops using deep learning–powered dynamic phenotypic analysis

**DOI:** 10.1093/gigascience/giaf129

**Published:** 2025-10-16

**Authors:** Jie Dai, Zhenjie Wen, Mujahid Ali, Jinlong Huang, Shuchen Liu, Jianhua Zhao, Felipe Pinheiro, Changcai Yang, Bin Wang, Lingzhen Ye, Xueying Guan, Ji Zhou

**Affiliations:** College of Engineering, College of Agriculture, Academy for Advanced Interdisciplinary Studies, Plant Phenomics Research Centre, Nanjing Agricultural University, Nanjing 210095, China; College of Engineering, College of Agriculture, Academy for Advanced Interdisciplinary Studies, Plant Phenomics Research Centre, Nanjing Agricultural University, Nanjing 210095, China; College of Engineering, College of Agriculture, Academy for Advanced Interdisciplinary Studies, Plant Phenomics Research Centre, Nanjing Agricultural University, Nanjing 210095, China; College of Engineering, College of Agriculture, Academy for Advanced Interdisciplinary Studies, Plant Phenomics Research Centre, Nanjing Agricultural University, Nanjing 210095, China; College of Engineering, College of Agriculture, Academy for Advanced Interdisciplinary Studies, Plant Phenomics Research Centre, Nanjing Agricultural University, Nanjing 210095, China; College of Engineering, College of Agriculture, Academy for Advanced Interdisciplinary Studies, Plant Phenomics Research Centre, Nanjing Agricultural University, Nanjing 210095, China; Data Sciences Department, National Institute of Agricultural Botany (NIAB) , Crop Science Centre (CSC), Cambridge CB3 0LE, UK; Center for Agroforestry Mega Data Science, School of Future Technology, College of Computer and Information Sciences, Fujian Agriculture and Forestry University, Fuzhou 350002, China; College of Engineering, College of Agriculture, Academy for Advanced Interdisciplinary Studies, Plant Phenomics Research Centre, Nanjing Agricultural University, Nanjing 210095, China; Zhejiang Provincial Key Laboratory of Crop Genetic Resources, Institute of Crop Science, Plant Precision Breeding Academy, College of Agriculture and Biotechnology, Zhejiang University, Hangzhou 310058, China; Zhejiang Provincial Key Laboratory of Crop Genetic Resources, Institute of Crop Science, Plant Precision Breeding Academy, College of Agriculture and Biotechnology, Zhejiang University, Hangzhou 310058, China; College of Engineering, College of Agriculture, Academy for Advanced Interdisciplinary Studies, Plant Phenomics Research Centre, Nanjing Agricultural University, Nanjing 210095, China; Data Sciences Department, National Institute of Agricultural Botany (NIAB) , Crop Science Centre (CSC), Cambridge CB3 0LE, UK

**Keywords:** seed vigor, germination, vision-based deep learning, dynamic trait analysis, wheat

## Abstract

**Background:**

As one of the most important cereal crops, wheat (*Triticum aestivum* L.) production and grain quality are essential to many nations in the world. Early developmental phases such as seed germination and seedling establishment are key to wheat’s growth and development as they impact directly on a crop’s early performance and yield potential. Hence, it is critical to develop varieties with favorable early growth characteristics under various growing conditions.

**Results:**

Here, we present SeedGerm-VIG, an automated and comprehensive pipeline developed for assessing seed vigor in wheat and other cereal crops. Building on the SeedGerm system, we integrated multiple deep learning models (i.e., YOLOv8x-Germ and optimized U-Net) and computer vision algorithms into the automated seed-level analysis pipeline to identify key germination phases and measure seed-, root-, and seedling-level phenotypic traits. Then, by using a time-series directed graph, we not only tracked root tips to reliably measure root emergence during the germination procedure (seed-lot *R*^2^ = 84.1%) but also established a new approach to examine speed and uniformity of seed germination. These resulted in the establishment of a vigor scoring matrix, through which 21 commercial genotypes’ (n = 494 randomly sampled seeds, with over 29,500 seed-level images) vigor scores were summarized and evaluated at key phases such as protrusion, radicle emergence, and chloroplast biogenesis. These measures largely matched with manual assessment based on the International Seed Testing Association (ISTA) guidelines. Finally, we also demonstrated that the SeedGerm-VIG pipeline could be used to assess seed vigor for other cereal crops, including rice (*n* = 120 seeds) and barley (*n* = 240 seeds), reproducibly.

**Conclusions:**

In conclusion, we believe that our work demonstrates a valuable step forward to enable a broader plant and crop research community to examine seed vigor and vigor-related phenotypic features in an automated manner, facilitating effective and scalable plant selection and relevant seed science research for crop improvement.

## Introduction

The imminent challenges of climate changes, growing population, and fertilizer shortage have brought diverse threats to global food security [1]. As one of the most consumed cereal grains in the world, wheat (*Triticum aestivum* L.) production and grain quality are vital to many nations in the world [2, 3]. Early developmental phases, such as seed germination (growth stage, GS 00–09) and seedling establishment (GS 10–19), are particularly critical for wheat growth and development as low-quality establishment often translates into (i) a reduced plant density and thus lower yield production, (ii) decreased crop effectiveness when competing against weeds, and (iii) the potential development of early-stage plant diseases [4–6]. Hence, better seed performance at phases such as protrusion, radicle emergence, and seedling establishment is likely leading to improved crop health and performance, ensuring yield potential under field conditions [7, 8].

In general, the combination of favorite germination and seedling establishment characteristics under varied growing conditions is defined as high seed vigor by the International Seed Testing Association (ISTA) [9]. As a complex trait, seed vigor is not only an critical aspect of seed quality [10, 11] but also key for breeders and researchers to genetically improve plants [7, 12]. Due to the importance of high-vigor features such as seed production capability and seed longevity under various storage conditions, seed vigor is widely accepted as an important subject in seed science research and crop improvement, forming the foundation of modern crop breeding, cultivation, agronomic management, and crop production [7, 12, 13].

Traditionally, assessing seed vigor involves many tests and experiments based on physiological and biochemical parameters of seed lots. For example, one popular method is to evaluate cumulative germination rates to identify high- or low-vigor groups of seed lots [14]. For such experiments, radicle length at specific time points (e.g., 48 hours after imbibition) was measured, through which the effectiveness of germination and seed viability was estimated [15]. Other methods such as accelerated aging [16], electrical conductivity [17], and seedling emergence [18] were also used, leading to the classification of different vigor groups. Recently, biochemical markers were employed to study vigor, including (i) sugar content (e.g., glucose and fructose), as sugars can negatively impact germination [19], and (ii) protein content, because it correlates with better seed performance [20]. Spectrometric reflectance and gas chromatography were also introduced to the research domain: (i) spectrometric reflectance collected by multi- and hyperspectral sensors and spectroscopies was applied to classify high- and low-performing seed lots [21, 22], and (ii) gas chromatography was employed to estimate seed vigor groups based on volatile organic compounds released during seed metabolism [23].

Among the above approaches, the use of morphological attributes of seeds (e.g., size and shape), radicles (e.g., length), and seedlings (e.g., establishment timing and rates) provides direct evidence in determining seed vigor. For instance, larger seeds were reported to have a higher vigor potential due to greater nutrient reserves [24]; the physical structure of the seed coat can influence water uptake during imbibition and thus affect the speed of seed germination [25]. Additionally, phenotypic variations of germination- and vigor-related features can lead to genetic studies of seed vigor, enabling assessment of seed quality and thus crop performance to accelerate crop breeding [26, 27].

Since the 21st century, advances in remote sensing, computer vision (CV), and deep learning (DL) technologies have opened a new door for evaluating germination- and vigor-related traits or aspects [11]. Using color, spectral, and morphological traits, diverse vision-based solutions have been introduced, including (i) *Pheno*Seeder [28] and *SeedExtractor* [29], developed to analyze size- and color-based traits based on red, green, and blue (RGB) seed imagery; (ii) *Germinator* [30] and *SeedQuant* [31], built to identify germination status using color and contrast features; (iii) *SeedGerm* [32] applied supervised machine learning (ML) to quantify cumulative germination rates for plant species such as tomato, pepper, and *Brassica*; (iv) RootNav 2.0 [33], using DL techniques to measure root features for wheat, *Arabidopsis*, and *Brassica*; and (v) hyperspectral imaging and DL models, which were combined to estimate oil composition [34] and chemical components (e.g., amino acids and lipids) to study seed quality [35].

Still, many of the above methods focus on measuring traits at a specific time point (e.g., protrusion or when radicles reach a certain length), which misses the dynamic nature of the early developmental phase as seed performance can fluctuate during germination, particularly when seeds are interacting with external stimuli [36]. After imbibition, radicles play a vital role in absorbing resources and supporting seedling establishment, enabling the active transition phase between heterotroph and photoautotroph [37, 38]. Hence, the ability to dynamically measure germination-related phenotypic changes at the seed level (e.g., changes of radicle length) will facilitate plant researchers and breeders to quantitatively examine seed performance, so that seed vigor can be evaluated objectively and comprehensively.

Here, we present SeedGerm-VIG, an open analytic pipeline developed to assess seed vigor for wheat and other cereals such as rice and barley. Building on the SeedGerm platform previously reported [32], we performed time-lapse imaging of wheat seed germination (for 4–7 days, depending on treatments), followed by the application of SeedGerm-VIG to (i) identify seed-level germination phases using the YOLOv8x-powered [39] DL model; (ii) detect seeds and seedlings (e.g., coleoptile) based on an optimized U-Net model [40], through which seed-level morphological features such as seed area, perimeter, length, width and roundness, seed coat color, and the seedling system can be measured; and (iii) track positions of radicles, lateral roots, and seedling tips over time to pinpoint their emergence rates. Finally, utilizing time points and growth rates of protrusion, radicle emergence, and chloroplast biogenesis (when seedlings turn green), we successfully establish a vigor scoring matrix to quantify this complex trait for 21 commercial genotypes at key developmental stages during germination, whose results are partially compatible with the ISTA’s guidelines [15].

## Findings

### Time-lapse seed germination imaging

**Figure 1:**
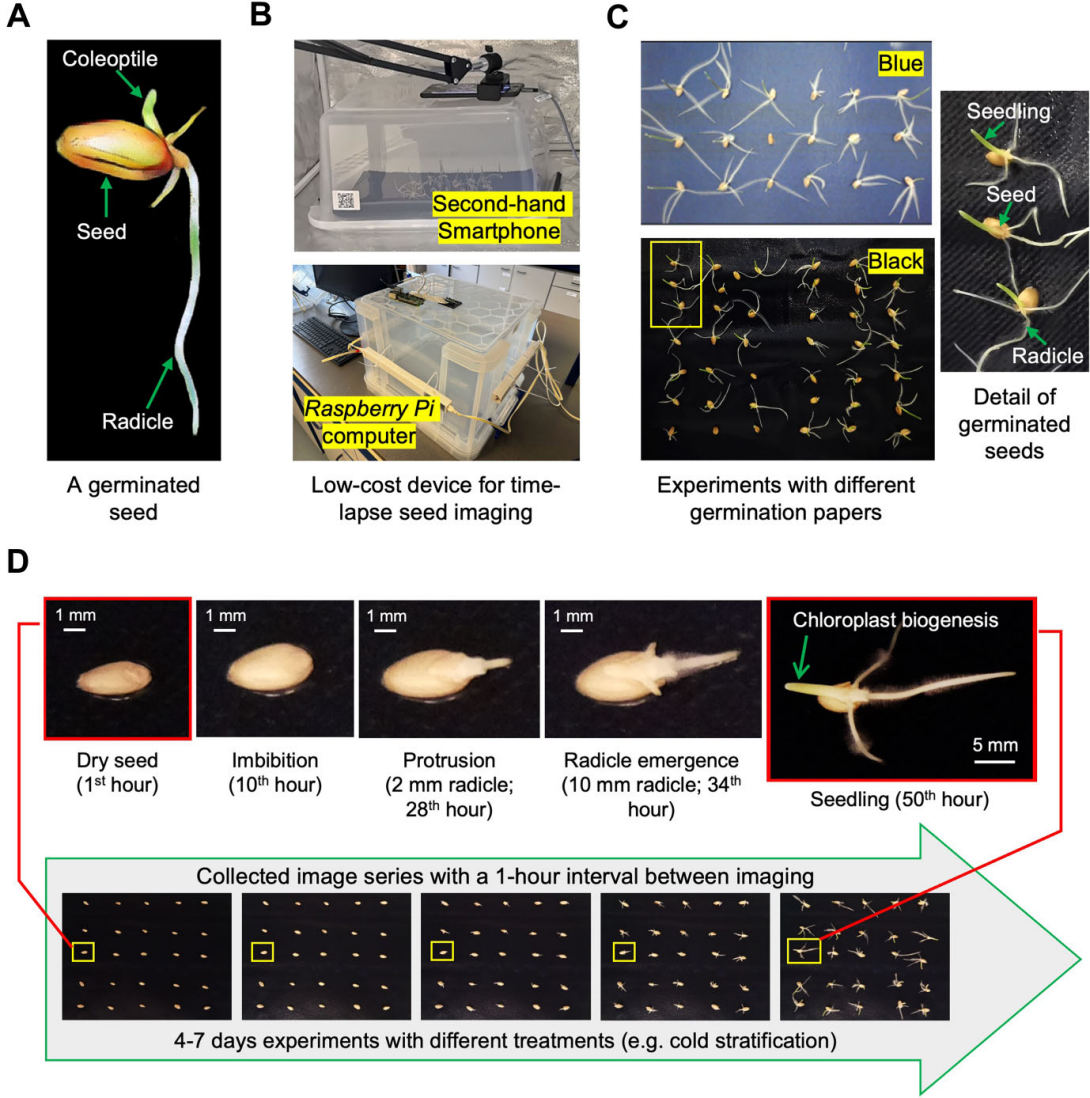
Time-lapse seed germination imaging and acquired image series using SeedGerm devices. (a) A germinated wheat seed consisting of coleoptile, seed and radicles. (b) A set of low-cost SeedGerm devices equipped with second-hand smartphone or *Raspberry Pi* sensors to perform overhead time-lapse imaging. (c) Germination experiments with blue and black germination papers. (d) Representative seed- and seed-lot level images of genotype 4 (G4) collected at key germination phases during the germination procedure. Scale bars provided to show the size of seed, radicles, and seedling.

We first established many low-cost SeedGerm devices [32] to perform RGB overhead time-lapse imaging of seed germination with high-definition (HD) seed-level images (Fig. 1A). Experiments were conducted in translucent plastic boxes, with either second-hand smartphones (with a maximum of 3,840 × 2,464 pixels per image) or *Raspberry Pi* image sensors (with a maximum of 4,608 × 4,608 pixels per image) mounted on the top (Fig. 1B). A series of RGB images were acquired with 1-hour intervals between each shot, recording from dry seeds and imbibition to seedling establishment after the chloroplast biogenesis phase (Fig. 1C, D). In total, 21 commercial wheat genotypes known for different germination paces were selected.

To improve the generalization of the SeedGerm-VIG pipeline, we trialed different experimental settings such as different seeds (e.g., 18, 20, 25, or 30 seeds) and blue or black germination papers (Fig. 1C). Depending on plant species, treatments (e.g., cold stratification and ambient temperatures), and potential germination speeds, time-lapse imaging was set from 96 hours (i.e., 4 days) to 168 hours (i.e., 7 days), with HD seed-lot image series (i.e., RGB images acquired in the experiments) stored and synchronized via OneDrive (Supplementary Table S1).

### A high-quality training dataset for assessing seed vigor

A seed-lot image series normally covers the entire germination procedure until the seedling is fully established (Fig. 1D, lower). For the 21 experiments performed in this study, a total of 1,890 seed-lot images were collected. To enable the DL model to identify germination phases for every seed in a given experiment, plant specialists first used LabelMe [41] to annotate germination phases based on seed-level images (Fig. 1D, upper), including 250 images at imbibition (IMB), 218 at protrusion (PRO), 278 during radicle emergence (RE), and 215 during seedling establishment (SE). These images were randomly selected from 48 seed-lot images with either black (19) or blue germination papers (29).

After image augmentation (e.g., adding noise and image flipping; see Methods), an augmented training set called “SeedVig-phase” was established, which contained 3,053 images, relatively evenly distributed across 4 key germination phases (i.e., 776 images at IMB, 720 at PRO, 784 during RE, and 773 during SE). Additionally, to perform background removal for accurate seed-level trait analysis, more images were annotated, including 644 seeds without roots and seedlings, 702 seeds with roots and seedlings, and 680 seedlings. Similarly, image augmentation was applied and created a training set called “SeedVig-traits,” consisting of 1,932 seeds, 2,106 seeds with roots and seedlings, and 2,040 seedlings. The 2 evenly distributed training sets (Supplementary Fig. S1) were both used in developing and benchmarking DL models.

### The YOLOv8x-Germ model for identifying key germination phases

**Figure 2:**
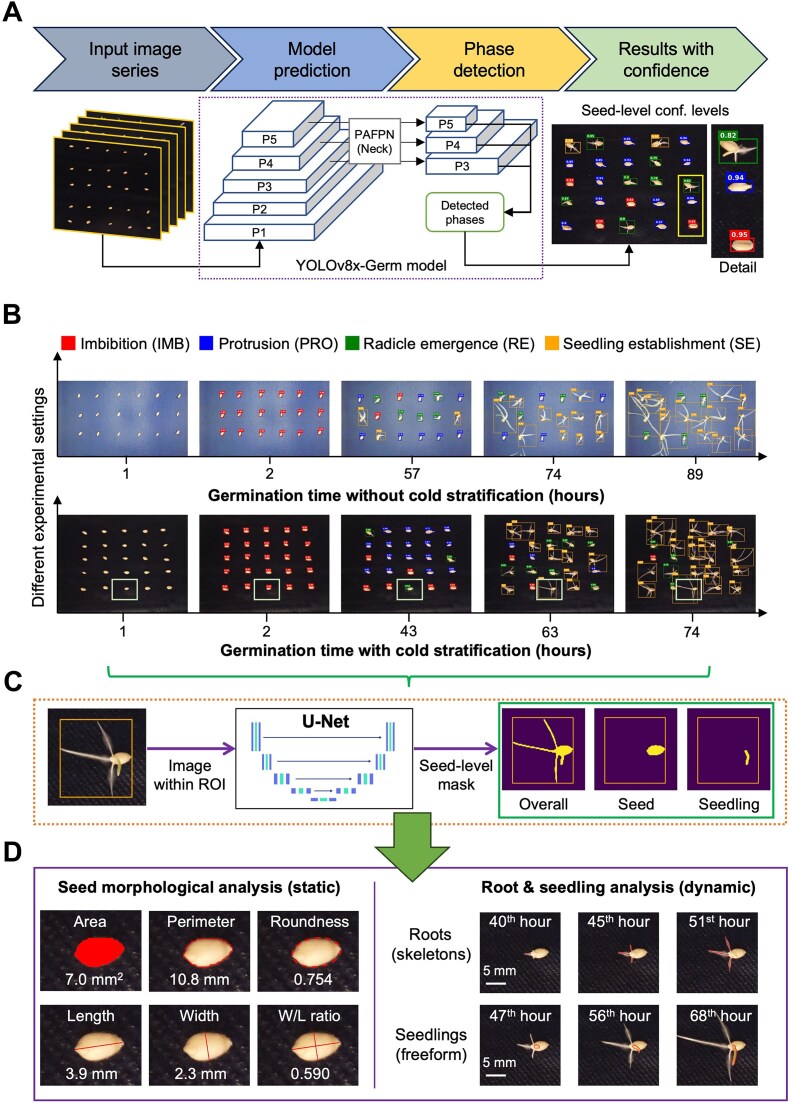
The analysis workflow of SeedGerm-VIG for identifying key germination phases for every seed in a seed lot, followed by the measurement of seed-level static and dynamic germination traits. (a) A general analysis workflow for seed-level germination phase identification using the YOLOv8x-Germ model, with seed-level confidence displayed. (b) Seed-level germination phases identified by the YOLOv8x-Germ model from seed-lot level image series (upper: G2; bottom: G7) together with bounding boxes, ranging from imbibition (IMB), protrusion (PRO), radicle emergence (RE), to seedling establishment (SE). For a given experiment, the germination phase (i.e. IMB, PRO, RE, or SE) was defined when over 75% of the seeds reached a certain phase. (c) Seed-level masks generated using an optimized U-Net model, dividing foreground objects (e.g. seeds, roots, and seedlings) from background signals (e.g. germination papers). (d) Phenotypic analysis of seeds, radicles and seedlings based on the identified foreground objects.

To automate the identification of key germination phases at the seed level in seed-lot image series, we developed a YOLOv8x-powered model called “YOLOv8x-Germ” using the “SeedVig-phase” training set, which was then embedded in the SeedGerm-VIG pipeline (Fig. 2A, upper). Seed-level bounding boxes were generated with color coding to indicate different germination phases (i.e., red, blue, green, and orange for IMB, PRO, RE, and SE phases, respectively), with confidence levels (0–1, with 2 decimal places retained) attached to signify the probability of the DL-based estimation (Fig. 2A, lower). The YOLOv8x-Germ model was used to identify seed-level germination phases and regions of interest (ROIs) in any seed-lot images collected under dissimilar conditions (Fig. 2B). According to the ISTA guideline [9], a specific germination phase is determined when at least 75% of seeds in a given seed lot reach the phase. We therefore used the unseen data in the “SeedVig-phase” dataset to evaluate the DL model’s performance when it was used to identify key germination phases for all the seeds in a seed lot. This resulted in highly accurate identification of germination phases at the seed-lot level, from IMB (100.0%) and SE (100.0%) to PRO (90.0%) and RE (83.3%) phases (Supplementary Fig. S2, right). To standardize DL-based phase identification for experiments with different numbers of seeds, 15 seeds from all the experiments (i.e., seed lots) were sampled (Supplementary Fig. S3).

### U-Net powered seed-level trait analysis and evaluation

To analyze germination- and vigor-related traits, after identifying seed-level bounding boxes (i.e., ROIs) of all the seeds in a seed lot, we developed a U-Net–based model to precisely remove background objects (i.e., black and blue germination papers, as well as root hairs) within the ROIs (Fig. 2C). Using a seed in genotype 7 (G7; row 5, column 3; highlighted in Fig. 2B), the U-Net model enabled us to retain seed, radicle, lateral roots, and seedling within the ROIs. Then, seed color and morphological traits, such as seed coat color, area, perimeter, roundness, length, width, and width and length (W/L) ratio, were quantified to examine dry and imbibed seeds (Fig. 2D, left), followed by the measurement of root-related (both radicles and 2 lateral roots) and seedling-related features, such as root length (using 2-dimensional [2D] skeletons), seedling size, and color (Fig. 2D, right). Table 1 summarizes all the static and dynamic traits quantified by the SeedGerm-VIG pipeline at 3 germination phases.

**Table 1:** The summary of traits quantified at 3 germination phases

Germination phases	Static traits	Dynamic traits
**Imbibition (IMB) and protrusion (PRO)**	Seed area (mm^2^)	Change rates of seed area (%)
	Seed length (mm)	Change rates of seed length (%)
	Seed width (mm)	Change rates of seed width (%)
	Seed perimeter (mm)	Change rates of seed perimeter (%)
	Seed W/L ratio (0–1)	
	Seed roundness (0–1)	
	Seed coat color red (0–255)	
	Seed coat color green (0–255)	
	Seed coat color blue (0–255)	
	Time point of PRO (seed level; hour)	
	Time point of PRO (seed-lot level; hour)	
**Radicle emergence (RE)**	Radicle length (mm)	Change rates of radicle length (mm/h)
	Root 2 length (mm)	Change rates of roots 2 and 3 length (mm/h)
	Root 3 length (mm)	
	Time point of RE (seed level; hour)	
	Time point of RE (seed-lot level; hour)	
**Seedling establishment (SE)**	Seedling length (mm)	Duration from coleoptile emergence to chloroplast biogenesis (seed level; hour)
	Time point of coleoptile emergence (hour)	
	Time point of chloroplast biogenesis (hour)	
	Time point of SE (seed level; hour)	
	Time point of SE (seed-lot level; hour)	

Computationally derived traits were evaluated against manual scoring using correlation analyses, resulting in significant correlations for these traits (*P* < 0.001; Supplementary Fig. S4), including seed length (*R*^2^ = 0.894), seed width (*R*^2^ = 0.859), seed perimeter (*R*^2^ = 0.881), seed area (*R*^2^ = 0.881), radicle length (*R*^2^ = 0.800), and seedling size (*R*^2^ = 0.791), which indicated the reliability of the SeedGerm-VIG pipeline in phenotypic analysis. Moreover, we compared traditional germination traits scored manually and the SeedGerm-VIG–derived trait analysis, including germination potential (GP) on the first, second, and third days (i.e., days 1, 2, and 3) of the experiment (i.e., GP_1_, GP_2_, GP_3_); germination index (GI_1_, GI_2_, GI_3_); mean germination time (MGT); and time to 50% germination (T_50_). The correlation coefficients between the 8 traditional and SeedGerm-VIG–derived traits range from 0.728 to 0.910 (Supplementary Fig. S5), demonstrating the reliability of the SeedGerm-VIG pipeline. Additionally, we computed the broad-sense heritability for the SeedGerm-VIG–derived traits, estimating the genetic influence on them (Supplementary Tables S2–S4).

### A graph-based tracking method to study root emergence

**Figure 3:**
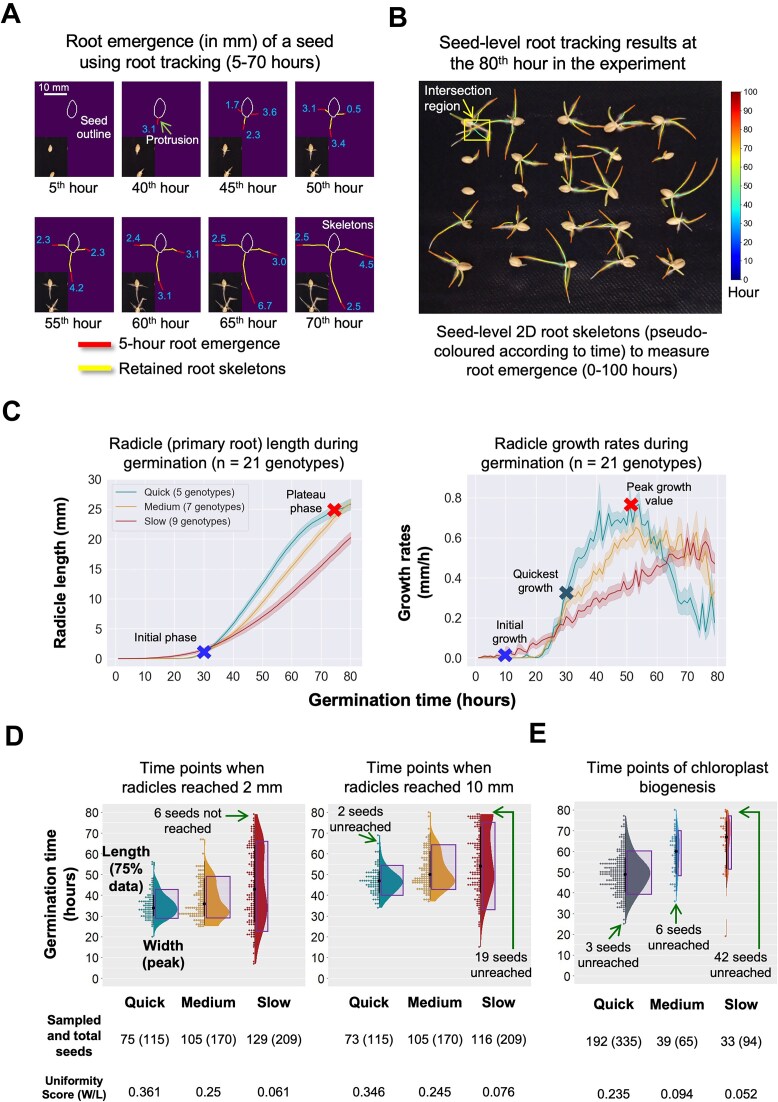
Tracking radicle and lateral root tips, growth speed profiles, and uniformity analysis during key germination phases. (a) Radicle emergence of a seed (genotype 7, G7) measured using the root tip tracking algorithm with 5-hour intervals. (b) Seed-level root tracking results of G7 at the 80^th^ hour, with root skeletons created and pseudo-coloured according to time (0-100 hours). (c) Profile curves of radicle length (left) and radicle growth rate (right) between 0 and 80 hours, which were classified into the Quick, Medium, and Slow Speed groups. (d) Raincloud plots used to demonstrate how uniform radicles in the three Speed groups reached 2 and 10 mm over time. (e) Raincloud plots show how uniform seeds reached chloroplast biogenesis in the three Speed groups between 0 and 80 hours. Arrows point out seeds did not reach key germination phases. Uniformity scores were provided based on 75% of the data obtained from the experiments.

To quantify timing, duration, and growth rate of root emergence (including radicle and lateral roots) for a seed lot during the germination procedure, we utilized the 2D radicle skeleton (Fig. 2D, upper right) to derive dynamic traits (e.g., germination speed and phase-based uniformity) for assessing seed vigor. A temporal directed graph was established to track radicle and lateral root tips for all the seeds in a given experiment. Using a given seed in G7 (row 1, column 2; Fig. 3A), the graph recorded 2D coordinates and growth direction of every primary root (i.e., radicle) and lateral root tips that emerged from the seed before root intersection, including (i) radicle and lateral root tips’ coordinates and positional changes within 5-hour periods, (ii) the distance and growth direction of the same root tip (red skeletons and blue growth distances, in millimeters; Fig. 3A), and (iii) cumulative root growth (yellow skeletons, in millimeters; Fig. 3A). Detailed algorithmic explanation is given (Methods; Supplementary Fig. S6).

When root intersection happened (lower, Fig. 3A), the root skeletons were extracted within root intersection regions (highlighted with a yellow solid rectangle; Fig. 3B). Intersected points, angles, and starting points of root skeletons, as well as the most likely growth direction (i.e., based on the previously recorded root graphs), were determined (Supplementary Fig. S7, left and middle), enabling us to associate root skeletons with their corresponding seeds within intersection regions. The above algorithmic steps were applied to track root tips throughout the germination procedure (Supplementary Fig. S7, right). For example, by tracking roots of 25 seeds (i.e., G7), we visualized the tracking result at the 80^th^ hour, where root skeletons were pseudo-colored according to time (i.e., 0–100 hours, from dark blue to dark red), followed by the measurement of seed-level root emergence based on assembled root skeletons. Noticeably, root skeletons did not exactly match with the 80^th^-hour image because they were assembled during a time when roots were slightly moved during the experiment (Fig. 3B). To ensure that the SeedGerm-VIG work could reach the broader plant research community, we created a graphical user interface (GUI) to include the above algorithmic steps using widget, so that nonexperts can use the pipeline relatively easily (see Methods).

### The growth patterns of radicles

Using the root tip tracking algorithm, we examined the 21 genotypes and their radicle emergence characteristics. According to the ISTA guideline [15], when the length of a radicle reaches 2 mm, the seed is “germinated.” When a seed’s radicle length reaches 10 mm, the seed’s root system is “established” [42, 43]. In our case, by quantifying radicle length at different time points with 1-hour intervals when radicles emerged to 10 mm (roughly between 0 and 80 hours), we were able to calculate 1-hour growth rates for all genotypes, leading to 21 radicle growth profile curves. All the genotypes followed a gradual increase pattern, with varying growth speeds (Supplementary Fig. S8). Using the radicle length as a metric, we applied the agglomerative clustering method [44] to categorize the 21 curves (Supplementary Table S5) into 3 categories: quick (5 genotypes; teal), medium (7 genotypes; orange), and slow (9 genotypes; dark red) groups (Fig. 3C, left). The differences between the 3 groups occurred at around the 30^th^ hour (highlighted with a blue cross) when seeds were entering protrusion. The quick group’s radicles began to slow down at the 75^th^ hour (highlighted with a red cross). Noticeably, quick root emergence did not necessarily lead to the longest radicle length as the medium group’s radicle length was slightly longer than those in the quick group.

Moreover, we studied radicle growth rates of the 21 genotypes, demonstrating varied patterns (Fig. 3C, right): (i) growth rates did not increase in a gradual pattern but instead fluctuated throughout the 80-hour period; (ii) timings of the highest growth rates of the 3 groups differed, with the quick group (teal) peaking at around the 50^th^ hour (denoted with a red cross), followed by around the 54^th^ and 70^th^ hours for the medium and slow groups, respectively; (iii) all groups experienced declines after the peak, with a sharp decline observable from the quick group; (iv) the slow group (dark red) exhibited an early initial growth (denoted with a blue cross), the smallest peak, and a relatively gradual increase trend compared with the other groups; and (v) by finding the first derivative of the growth rate curve, we were able to locate the time point when radicle growth was changing at the quickest rate (indicated with a dark teal cross), suggesting the most active phenotypic changes of radicle length. For the above curves (Fig. 3C), shaded regions were provided to represent the confidence interval (75%).

### The uniformity scoring of vigor-related features

Building on the measures of growth patterns (i.e., germination speed), we further studied how to derive uniformity of the seed lots during the germination procedure. For the 2 radicle emergence stages (i.e., 2 mm for germination and 10 mm for root system establishment), we employed raincloud plots (the x-axis shows the 3 growth groups) to present the distribution of time points (y-axis) when seed lots entered germination and root establishment (Fig. 3D): (i) the quick group (teal) had a narrow and concentrated distribution, with most values clustered around 30 to 40 hours; (ii) the medium group (orange) had a broader and more stretched distribution, with a central tendency around 30 to 50 hours; and (iii) the slow group’s (dark red) data distribution was the widest and most spreading, with values covered across the y-axis and some seeds not reaching target radicle lengths (i.e., 2 and 10 mm, indicated with green arrows). Because the 3 groups had dissimilar ranges and centered values when the radicle reached the 2 lengths, we therefore computed the ratio of the peak value (when most of the seeds reached 2 and 10 mm) and coverage of 75% of the data (highlighted with purple rectangles in Fig. 3D), resulting in a uniformity score (0–1) measuring how similar a given germination speed group elongated during different phases. This enabled us to effectively differentiate the 3 groups in terms of the RE uniformity, showing that genotypes belonging to the quick group had higher uniformity scores (i.e., 0.361 and 0.346 at the 2 phases) compared to the genotypes in the medium (around 30% less than the quick group) and slow (over 80% less) groups.

Similarly, using the time points when chloroplast biogenesis occurred (seedling turned green; Supplementary Table S6), we applied affinity propagation clustering [45] to classify the genotypes without predefining cluster number, followed by the analysis of their data distribution and uniformity (Fig. 3E). Three categories were identified. While the quick group (gray) exhibited the broadest distribution and the slow group (light red) the narrowest, genotypes in the quick group had a higher uniformity score (0.235) compared to those in the medium (light blue; around 60% less) and slow (over 70%) groups. Additionally, using the raincloud plots (Fig. 3D, E), we identified seeds that did not reach a certain phase. For example, 6 (of 129) and 19 (of 116) seeds in the slow group did not reach 2-mm and 10-mm radicle lengths, whereas most seeds in the quick and medium groups established the root system successfully. As for the chloroplast biogenesis, the slow group had 42 (44.7%) seeds that did not reach this phase during the 80-hour monitoring period compared with only 3 seeds (0.9%) in the quick group and 6 seeds (9.2%) in the medium group.

### A comprehensive assessment of seed vigor

**Figure 4:**
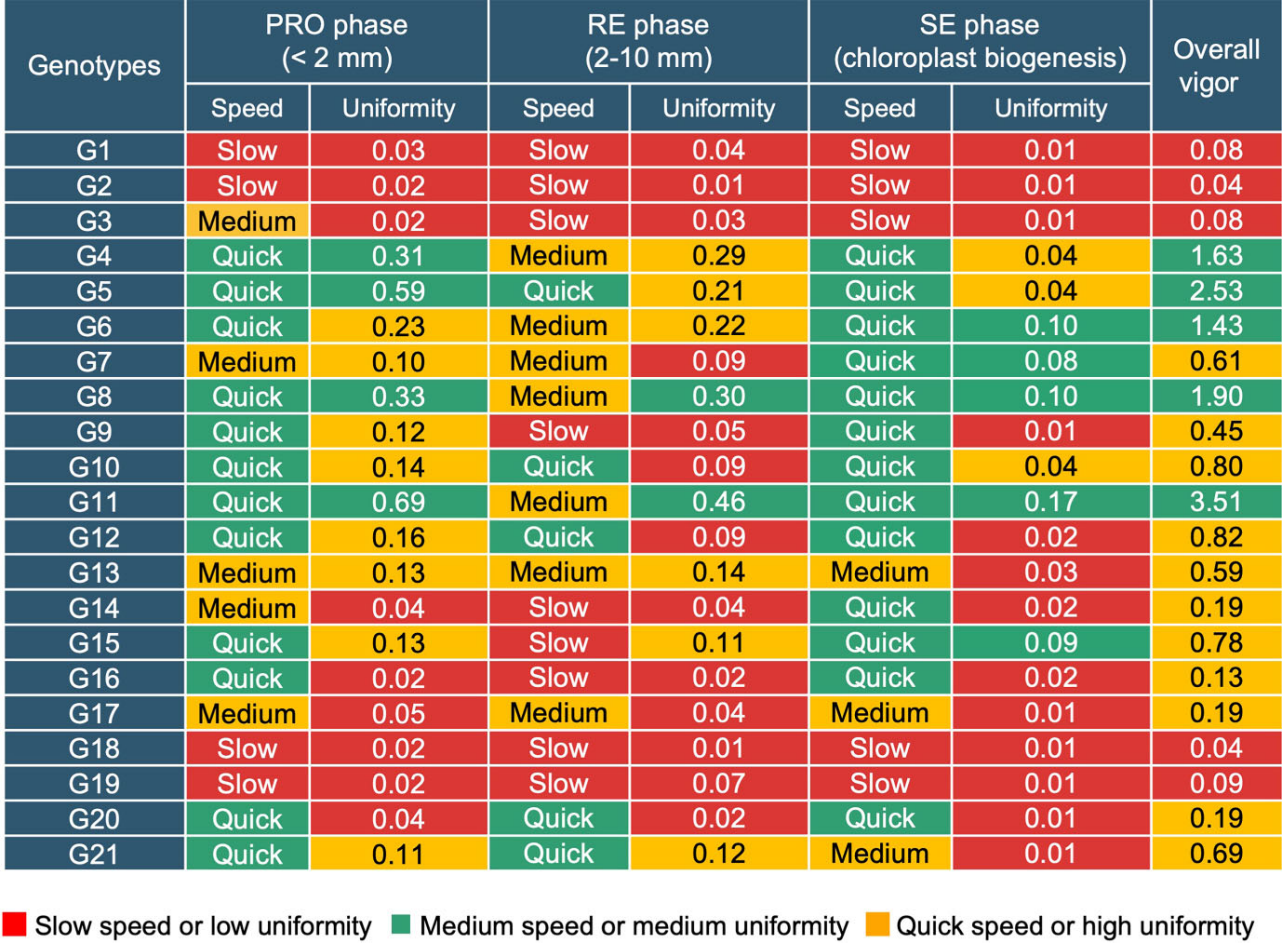
A comprehensive matrix provides an overview of 21 wheat genotypes’ germination speed groups (pseudo-colored) and uniformity scoring (with 2 decimal points) at 3 key phases, followed by the computation of overall seed vigor scores based on measures of phase-based speed and uniformity for the 21 genotypes.

Traditional seed vigor assessment often missed the dynamic nature of this complex trait as its performance could vary throughout the germination procedure. Hence, to facilitate an efficient and reproducible method to assess seed vigor, we created a comprehensive matrix to incorporate germination speed and uniformity at different germination phases into the assessment of seed vigor (Fig. 4). Using the phase-based classification of growth patterns (i.e., speed) and associated uniformity scores computed for the 21 genotypes (Supplementary Tables S7 and S8), we categorized the genotypes into 3 groups during the PRO, RE, and SE phases using the affinity propagation method [45]. Then, we set the quick group as 3 points, medium as 2 points, and slow as 1 point and multiplied them with their uniformity scores (i.e., speed × uniformity), which resulted in 3 overall vigour groups: (i) 5 low-vigor genotypes (G1, G2, G3, G18, and G19), (ii) 11 medium-vigor genotypes (G7, G9, G10, G12, G13, G14, G15, G16, G17, G20, and G21), and (iii) 5 high-vigor genotypes (G4, G5, G6, G8, and G11). We compared the above vigor results with traditionally assessed vigor groups (i.e., 5 low-vigor genotypes and 16 high-vigor genotypes; Supplementary Table S9) and found that the manually scored low-vigour (*n* = 5) and high-vigour (*n* = 5) genotypes matched with the SeedGerm-VIG–derived lines, while the medium-vigor group could not be assessed as manual scoring could not identify genotypes with this feature.

## Discussion

As a critical and complex agronomic trait in cereals, seed vigor demonstrates plant seeds’ ability to germinate, establish seedlings, and sustain early growth under varied growing conditions [7]. As seed vigor is governed by multiple genetic, biochemical, and physiological factors [14], it is difficult to conduct a comprehensive study through traditional approaches. Still, because high-vigor seeds often lead to uniform crop establishment and thus reduced risks of crop performance (e.g., poor establishment and seedling mortality) under field conditions, breeders and researchers are keen to study and incorporate this trait in the breeding programs to sustain crop production [46]. Moreover, seed vigor is also treated as a vital target for developing climate-resilient crops, providing plants with a competitive edge against external stimuli at early developmental phases [47]. As previous studies on seed vigor often focused on germination-related traits measured at specific time points, which missed the dynamic and complex nature of seed vigor [26], we therefore developed the SeedGerm-VIG methodology, an open and automatic analytic pipeline for quantifying seed-, root-, and seedling-related traits and their phenotypic changes during key germination phases, through which we could empower the research community with a new toolkit to assess seed vigor in a comprehensive and dynamic approach. We trust that our toolkit has made advances in a number of areas for seed science research.

### The SeedGerm-VIG pipeline for assessing vigor-related traits

Due to recent advances in vision-based DL techniques, DL solutions have been widely applied to plant phenotyping-related studies [48]. Using the SeedGerm platform for time-lapse imaging, we integrated 2 trained DL models (i.e., YOLOv8x and U-Net) into the SeedGerm-VIG pipeline to analyze time-series images collected in dissimilar experimental settings (e.g., the number of seeds, seed treatments, and germination papers), which outperformed several classical and latest DL models (Tables 2 and 3). This enabled us to establish an automated approach to quantify seed-, root-, and seedling-related traits. For example, the YOLOv8x-Germ model was trained to perform the seed-level identification of key germination phases (i.e., IMB, PRO, RE, and SE) for every seed in a seed lot (Fig. 2B), which helped us identify seed-level germination progress and facilitate the segmentation of seed-level ROIs for the following trait analysis. With limited training datasets (3,053 seeds), the YOLOv8x-Germ model achieved high-accuracy predictions of the IMB (84.7%), SE (89.5%), PRO (77.0%), and RE (84.1%) phases based on 1,207 seeds (Supplementary Figs. S1 and S2), which can be easily improved when more training datasets are made available through our openly accessible pipeline.

**Table 2:** Performance analysis of different DL models used for seed detection

Model	mIoU (%)	mPA (%)	Accuracy (%)
U-Net	88.96	92.99	99.10
PSP-Net	82.30	88.00	98.34
HR-Net	83.87	89.60	98.49
DeepLabV3+	80.26	88.07	98.33

**Table 3:** Performance analysis of different DL models trained for identifying germination phases

Model	Precision (%)	Recall (%)	mAP50 (%)	F1 (%)
YOLOv8	88.10	94.30	95.30	91.11
SSD	82.20	56.75	67.70	61.75
Faster-RCNN	63.38	41.98	52.14	50.25
RT-DETR	72.10	79.30	76.60	75.10

Additionally, within the seed-level ROIs, we used an optimized U-Net model to generate masks of seeds with or without roots and seedlings (Fig. 2C), leading to the analysis of seed-level morphological features, root (both radicle and lateral roots) length and tips, and seedling size. According to the correlation analyses (Supplementary Fig. S4), most of the computationally derived traits were significantly correlated with manual scoring, demonstrating the reliability of the pipeline. Notably, by combining trait analysis with graph-based root tip tracking, we further developed dynamic measures of vigor-related traits by quantifying cumulative root length based on tracking the emergence of roots on an hourly basis (Supplementary Fig. S9), through which radicle growth profiles were produced to illustrate the 21 genotypes and their germination speed groups (Supplementary Fig. S8). This also led to the curves of radicle growth rates, providing insights into radicle growth patterns during germination, from 0 to 80 hours.

In particular, we employed a range of computational traits to collectively describe seed vigor, including (i) by using seed size during IMB to assess the speed of imbibition (Supplementary Fig. S10), (ii) by quantifying radicle and lateral root emergence (Supplementary Fig. S11) to quantify time points and duration of PRO and RE phases, and (iii) by monitoring the presence of greenness on seedlings to obtain the timing of chloroplast biogenesis. Equipping with the above measures, we were able to dissect seed vigor, a highly complex and variable trait, into vigor-related subtraits to study the trait comprehensively. Compared with our previously published *SeedGerm* platform [32] that was developed for seed-lot level germination analysis, our study partially inherited its hardware design and seed-lot level source codes for automated image processing. Still, for assessing temporal seed-level analysis, instead of traditional ML models, the SeedGerm-VIG pipeline incorporated DL models and graph-based computer vision algorithms to improve the generalization and accuracy, enabling seed-level trait analysis and vigor-related feature assessment in acquired image series.

### The assessment of seed vigor using germination speed and uniformity

In the context of crop improvement, seed vigor can help us select genotypes that are more resilient under different environment and aging conditions [7]. How to assess vigor-related features when seeds are interacting with external stimuli can provide us with insights into the selection of varieties with better crop establishment, productivity, and adaptability in diverse agricultural settings [46]. In our study, besides quantifying timing and speed to evaluate seed performance over time, we also explored the calculation of uniformity, which is another favorite high-vigor feature advised by ISTA [49]. We used raincloud plots to exhibit the data distribution when seeds entered the PRO, RE, and SE phases. While the time points of seeds entering a key phase can be used to assess the germination speed and hence the cumulative germination rates, the raincloud plots also assisted us to examine the data distribution of how many seeds entered a given phase over time. For example, using 75% of seeds when they entered a germination phase as “length” and the peak value of the data distribution as “width” (Fig. 3D, left), we computed the ratio of the width and the length (i.e., W/L), which was also proven to be reliable using heritability, coefficient of variation, and interquartile range (Supplementary Tables S2–S4, S10, S11), deriving a metric (0–1) to represent the uniformity of radicle emergence and chloroplast biogenesis. The metric could effectively distinguish dissimilar uniformity patterns for the 21 wheat genotypes, as well as the 3 germination speed groups. Finally, we proposed a seed vigor matrix to incorporate germination speed and uniformity at the 3 phases into the identification of overall seed vigor, resulting in 5 high-vigor, 11 medium-vigor, and 5 low-vigor genotypes, which largely correlated with the manual assessment following the ISTA guideline. To our knowledge, we originated this method to advance automated seed vigor assessment, integrating germination speed and uniformity to identify high-performing seed lots quantitatively.

### The application of SeedGerm-VIG for other cereal crops

**Figure 5:**
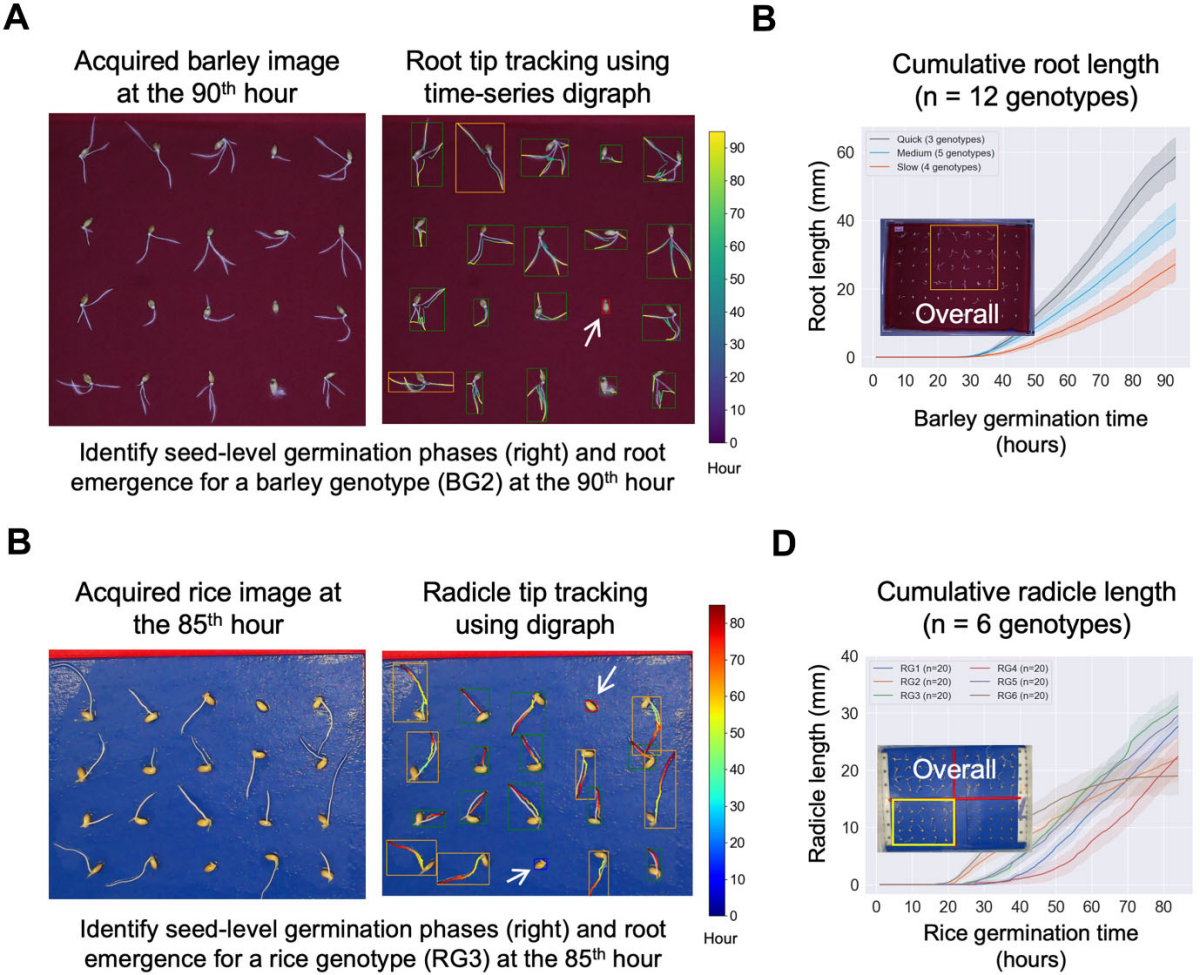
The application of the SeedGerm-VIG pipeline to perform seed-level analysis of root (barley) and radicle (rice) emergence for 12 commercial barley and 6 rice genotypes. (a) Barley seeds germination recorded on dark red germination papers and seed-level root emergence identified by the SeedGerm-VIG pipeline for a barley genotype (BG2) at the 90^th^ hour, with seed-level bounding boxes showing seed germination phases (one seed still at protrusion was pointed with a white arrow) and root skeletons pseudo-coloured according to time (0-90 hours). (b) Root emergence curves produced to demonstrate germination speed and profiles for twelve barley commercial lines (15 seeds sampled per line) over 93 hours. (c) Rice seeds germination recorded on light blue germination papers and seed-level radicle emergence identified by the SeedGerm-VIG pipeline for a rice genotype (RG3) at the 85^th^ hour, with seed-level bounding boxes showing germination phases (one seed was still at imbibition and one seed was at protrusion) and radicle skeletons pseudo-coloured according to time (0-85 hours). (d) Radicle emergence curves produced to show germination speed and profiles for six rice commercial lines (15 seeds sampled per line) over 85 hours.

The SeedGerm-VIG pipeline enabled us to perform germination- and vigor-related trait analysis across the germination procedure, facilitating us to examine desired characteristics such as rapid germination, efficient establishment of root and seedling systems, and the smooth transition from root establishment to photosynthetic autotrophy under varied growing conditions (e.g., cold stratification and different temperatures). To study the generalization of the proposed method, we further applied the SeedGerm-VIG pipeline to test other cereal crops such as barley and rice through transfer learning [50], so that the pretrained DL models could be quickly fine-tuned with significantly reduced epochs and smaller training sets, indicating the potential use of DL techniques for multispecies studies. For barley, we carried out experiments with dark red germination papers for 12 commercial lines (i.e., barley genotype, BG; 20 seeds per line) for 90 to 95 hours (Fig. 5A, left).

For example, the YOLO8s-Germ identified germination phases at the 90^th^ hour with colored bounding boxes, highlighting an ungerminated seed (column 4, row 3; enclosed with a red box), which was still at the IMB phase (Fig. 5A, right). Then, the graph-based root tracking algorithm was applied to assess seed-level barley roots, generating pseudo-colored root skeletons that represented root emergence time (Fig. 5A, right). Similar to wheat experiments, we computed cumulative root length of the 12 genotypes using 15 sampled seeds, clustering 3 speed groups for the barley lines (Fig. 5B; Supplementary Table S12). For rice, SeedGerm-VIG was applied to process 6 commercial rice genotypes (RGs) trialed on light blue germination papers, identifying seed-level germination status at the 85^th^ hour (with 1 seed ungerminated and 1 seed not reaching the PRO phase, both of which were highlighted with colored bounding boxes), with tracked rice radicles and thus RE speed groups (Fig. 5B, D).

Additionally, we performed correlation analyses between computationally derived traits and manually scored radicle length for 12 barley (*R*^2^ ≥ 0.793; *P* < 0.001) and 6 rice genotypes (*R*^2^ = 0.815; *P* < 0.001), demonstrating the reliability and generalizability of the SeedGerm-VIG pipeline for other cereal plants (Supplementary Figs. S12 and S13). The uniformity scores also were computed, which closely corresponded to the germination patterns across all 3 speed groups for barley when the radicles reached either 2 or 10 mm (Supplementary Fig. S12b), confirming the practical value of the vigor index.

### Limitations and future developments

Building upon the ISTA guidelines, this study advanced the assessment of seed germination and vigor from seed lots to the seed level, which empowered us to study seed-level performance during the germination. Still, we have encountered several limits for the SeedGerm-VIG pipeline when suboptimal imaging conditions, root occlusions, and excessive seed or root movements were witnessed (Supplementary Note S2). Some cases could be improved in future studies, including (i) image quality, which could be affected at early phases due to reflection on the germination paper surface as too much water was applied, which could be mitigated by controlling water applications at different time points; (ii) complex root intersection, which could lead to low accuracies of root emergence measures during RE and SE phases, which might be rectified by more labeled training data, cutting-edge DL models, and a focus on radicles with more frequent overhead imaging (e.g., 10 minutes per shot when radicle tip changes are limited); (iii) seed and root movement, which complicated seed positions and root tip tracking (see the algorithmic steps in Supplementary Note S1), with a consideration of how to prevent seeds from excessive movement (e.g., 3D printed seed holders); (iv) the generalization of the solution, which can be continuously improved with more wheat, barley, and rice genotypes included in the training dataset and thus the refined DL models; and (v) the detailed requirements outlined in ISTA’s guidelines (e.g., distinguishing between normal and abnormal seedlings), which will be considered as they can improve the practicality of our pipeline. Additionally, due to the limitation of 2D photography, low-cost devices such as smartphones mounted on turntables can be considered to reconstruct 3D color point clouds [51], based on which seed germination and vigor can be assessed more comprehensively. Notably, due to the open-source nature of our work, we believe that a community-driven effort could help verify and improve the performance and accuracy of the seed vigor assessment based upon the SeedGerm-VIG pipeline and its easy-to-use GUI.

Besides the above limits, it is valuable to point out that the study presented here is likely opening up new opportunities for us to reveal the genetics of seed vigor, which involve multiple genes and regulatory networks that influence the physiological and biochemical processes. Rapid germination, uniform root and seedling growth, stress tolerance, and even seed longevity are controlled by the interactions of genetic factors and external stimuli. Static and dynamic subtraits presented in this study can be used to gain an in-depth understanding of the genetic basis of seed vigor, including (i) genetic components of seed vigor that regulate seed metabolism, seed dormancy and germination, biotic and abiotic stresses, and seed coat and structural features; (ii) genetic mapping to identify genetic regions associated with seed vigor using static and dynamic traits, which can identify loci that regulate desired vigor-related features, stress tolerance, and seed quality; and (iii) crop breeding for seed vigor, which enables breeders to incorporate favorable alleles into new crop varieties through marker-assisted selection (MAS) and genomic selection in climate-resilient breeding programs [52], ensuring better crop establishment, productivity, and resilience to climate change.

## Materials and Methods

### Plant materials and seed germination experiments

To assess seed vigor in wheat with phenotypic variations, we selected 21 commercial wheat genotypes. These varieties were chosen to capture a broad range of developmental paces during germination (Fig. 1D). Seeds were produced in prebreeding field trials conducted in Cambridgeshire, United Kingdom (52°23′85″N, 9°60′99″E), and were used within 6 months of harvest. Before the experiments, seed lots were stored at 10°C and 10% to 15% relative humidity to maintain viability. To evaluate germination speed, some seed lots were treated with cold stratification at 5°C for 24 to 72 hours in sealed plastic bags (Supplementary Table S1).

### Time-lapse imaging with different SeedGerm settings

Germination experiments were carried out in 2022 and 2023 in the United Kingdom. Two types of low-cost SeedGerm devices were built for time-lapse imaging: (i) a small translucent plastic germination box (dimensions: 31 × 39 × 48 cm) mounted with fixed second-hand Android smartphones (equipped with Sony IMX278 or IMX319 image sensors; a maximum of 3,840 × 2,160 pixels per image, roughly 5,000 pixels per seed), and (ii) a big germination box (dimensions: 31 × 44 × 71 cm) mounted with 2 types of *Raspberry Pi* cameras (i.e., Sony IMX477 or IMX219, with a maximum of 3,280 × 2,464 pixels per image; roughly 5,500 pixels per seed). Different RGB sensors acquired HD images with different resolutions for a flexible hardware design (see image sensors in Supplementary Table S1). Germination experiments were carried out on blue (Bärenstein) and black (Crown Supplies) seed testing paper under an ambient temperature of 20°C to 22°C for 4 to 7 days depending on the duration of the cold treatments (i.e., 24, 48, and 72 hours). Time-lapse imaging was conducted at regular intervals of 1 hour, covering from imbibition (Fig. 1D, left) to seedling emergence, after chloroplast biogenesis when at least 10-mm green seedlings were visible (Fig. 1D, right).

### Data annotation and augmentation

LabelMe [41] was used for data annotation. For the phase detection task of each seed during germination, a total of 48 images were labeled, covering the 4 germination phases, including 29 images of blue background and 19 images of black background. Due to the resolution differences and growth directions, augmentation methods (e.g., adding noise and flipping) were chosen to mimic imaging-related problems. After augmentation, a total of 151 images (which is called “SeedVig-phase”) were randomly divided into training, testing, and validation sets in a 7:2:1 ratio. For the mask prediction task, we labeled the seeds without roots and seedling, seeds with roots and seedling, and seedlings at early, middle, and late time points in the image series, respectively. A total of 32 images for seeds without roots and seedling, 36 for seeds with roots and seedlings, and 38 for seedlings were annotated. Due to the luminance change in different germination experiments, brightness adjustment was applied for image augmentation, and finally 96, 108, and 114 images were generated for seeds without roots and seedling, seeds with roots and seedling, and seedlings, respectively. The images (called “SeedVig-traits”) were divided randomly into 70% (for training) and 30% (for testing).

### DL model selection and evaluation metrics

To detect seed objects using DL-powered models efficiently and accurately, we trained 4 DL models using the “SeedVig-traits” dataset with roots and seedlings, including U-Net [40], PSP-Net [53], HR-Net [54], and DeepLabV3+ [55]. When testing these DL models, every model was trained for 300 epochs, and their performance was evaluated through metrics such as mean intersection over union (mIoU), mean pixel accuracy (mPA), and accuracy. Through the evaluation, the U-Net model was selected as the architecture to train a DL model for seed objects segmentation (Table 2).

Similarly, we trained 4 DL models using the “SeedVig-phase” dataset to identify germination phases accurately, including SSD [56], Faster-RCNN [57], YOLOv8 [39], and RE-DETR [58]. When testing these DL models, every model was trained for 100 epochs, and their performance was evaluated through metrics such as precision, recall, mAP50 (mean average precision at IoU = 0.5), and F1 score. Through the evaluation, the YOLOv8 model was selected as the architecture for the identification of seed germination phases (Table 3).

### The SeedGerm-VIG pipeline for identifying germination phases

Images of blue germination paper were cropped according to ROIs. For images of black germination paper, rotation or translation was applied to correct slight camera shifts at the start of the image series. After establishing the training set, the standard YOLOv8x model [39] was trained for 2,000 epochs, which was used to identify seed-level germination phases in the experiment. A seed lot’s germination phase was defined when 75% seeds of the seed lot entered a specific phase (Fig. 2B).

### The SeedGerm-VIG pipeline for seed-level trait analysis

After establishing the training set, we followed the previously reported approach [40] and used the pretrained VGG-16 [59] architecture as the backbone (encoding part) of the standard U-Net model [60]. 3 U-Net models were trained for 1,000, 400, and 400 epochs to predict masks of seed coat, overall, and seedling of each seed, respectively. The seed morphologies, including seed area, seed length, seed width, seed perimeter, seed W/L ratio, and seed roundness, were quantified through a previously described method [32], while seed colors were quantified by the mean values of R, G, and B channels in the RGB color space of the seed objects (Supplementary Table S13). The change rates of morphological features were quantified based on the obtained static phenotypes (i.e., values at different time points).

Using a root tip tracking algorithm, root tips’ positional changes were recorded as the corresponding root change rates, and cumulative root lengths were quantified by reconstructing the lines based on the positional changes of root tips across image series (Supplementary Fig. S6). Seedling emergence was recorded when the seedling mask first overlapped with the mask of seeds without roots and seedlings within a ROI (i.e., bounding box). At the 80^th^ hour, the regions corresponding to the seedling mask were converted to excess green (ExG) values [61], and the chloroplast biogenesis threshold (greenness value) was computed based on the mean of ExG values [62]. If the ExG value of any pixel in the seedling mask across image series exceeded the threshold, the time point was recorded as chloroplast biogenesis. The duration from coleoptile emergence to chloroplast biogenesis was quantified based on the time points.

### Graph-based root emergence tracking at the seed level

Based on the ROIs obtained, the predicted masks of seeds without roots and seedling and seeds with roots and seedling were used for root tip tracking. After extracting the skeletons (Fig. 3A), the root tips were determined by removing skeletons that overlapped with the seed coat and seedling. Then, the root tips were numbered according to their emergence order, with the positional changes of root tips quantified based on their coordinates. As germination progresses, roots often intersect, complicating tracking efforts. To address this issue, we developed algorithms to segment 3- and 4-vertex cliques [63]. After extracting skeletons (Supplementary Fig. S7), branch point (red) or equivalent branch point (i.e., the midpoint of 2 branch points; blue) was identified, alongside root tips (green) within the local region. Vectors were then constructed from these (equivalent) branch point to the root tips, so that the intersected skeletons could be segmented based on the vector angle, with preference given to angle closer to 180°. Finally, the 2D skeletons were assembled for later analysis.

Building on root tip coordinates and intersection segmentation algorithm, this study utilized a temporal graph [64] to track root tips. Although the SmartRoot toolkit [65] was reported to use a 90° scanning range to search for intersection roots from a side perspective, the 60° scanning setting used in this study for identifying intersection roots from overhead images proved more useful in tracking root tips in complex root emergence images. Using seed 2 of G7 (row 1, column 2) as an exemplar (Fig. 3A), each root was treated as a temporal graph where the initial vertex ${V}_0$ and edge ${E}_0$ were both $\emptyset$. The primary root (radicle) emerged at the 40th hour (${t}_e$), where *V_te_s_* denoted the skeleton point connecting to the seed coat and the root tip was taken as *V_t_*, with the skeleton connecting the 2 nodes defined as ${E}_t$. To continuously track root tips of different roots, routes connecting *C*t (current root tips) to *V_t_*  _− 1_ were taken into consideration, along with their angles. For instance, at time point *t +* 1, iterating the root tips in *C*t, when root tip *P* met two requirements: (i) the route connecting *P* to *V_t_* had the minimum weight (where skeleton points weighed 0 and others weighed 1), and (ii) the angle between $\overrightarrow{\mathop {{V}_{t - 1}\ {V}_t}}$ and $\overrightarrow{{V}_{t}P}$ was no more than 60°, the *G_t_*  _+ 1_ was updated accordingly. Through this process, the radicle growth process could be tracked and quantified (Fig. 3A). To avoid tracking errors, after radicle intersections, newly emerged radicles were not tracked. Formulas used to perform the tracking are listed as follows:


\begin{eqnarray*}
{G}_t = \left\{ {\begin{array}{@{}*{2}{l}@{}} {({V}_0,{E}_0),}& \quad {{\mathrm{if }}t < {t}_e - 1}\\ {({V}_{{t}_e\_s},{E}_0),}& \quad {{\mathrm{if }}t = {t}_e - 1}\\ {({V}_t,{E}_t),}& \quad {{\mathrm{if }}t >{t}_e - 1} \end{array}} \right.
\end{eqnarray*}

\begin{eqnarray*}
\left\{ {\begin{array}{@{}*{1}{l}@{}} {{V}_{t + 1} = {V}_t \cup \{ P\} }\\ {{E}_{t + 1} = {E}_t \cup L({V}_t,P)} \end{array}} \right.
\end{eqnarray*}

where at time point *t* + 1, *P* should meet $P \in {C}_{t + 1}$, when $\| {{V}_t - P} \| = {\min }_{{P}_l \in {C}_{t + 1}}\| {{V}_t - {P}_l} \|$ and $cos( {\overrightarrow {{V}_{t - 1}\ {V}_t} ,\overrightarrow {{V}_{t\ }P} } ) \ge 0.5$.

### Statistical analysis and manual scoring

After trait analysis, the scales provided in Supplementary Table S1 were used to convert the pixel units into millimeters. To verify the performance of the SeedGerm-VIG pipeline, a total of 84 images (4 images per genotype), covering the early, middle, and late stages of seed germination experiments, were randomly selected for manual scoring. During the IMB phase, seed length, seed width, seed perimeter, and seed area were measured manually using ImageJ [66]. In the subsequent phases (i.e., PRO, SE, and RE), the lengths of roots and seedlings were assessed manually. Similarly, a total of 18 images of rice and barley were selected for manual measurement of root lengths. To verify the seed vigor of different wheat genotypes, the seed germination images at the 80^th^ hour were used for assessment. Clustering analysis was performed using the “Scikit-learn” library [67], while correlation analysis utilized the Pearson correlation coefficient and *P* value after removing the outliers.

To our knowledge, we could not find any existing toolkits that could be used to quantify the emergence of radicles and seedlings from overhead imagery over time. Hence, we chose 2 representative research tools, such as *SeedExtractor* [29] and *SeedGerm* [32], to evaluate seed-level morphological and color traits between the SeedGerm-VIG pipeline and the 2 software packages. The correlation analyses indicated a strong correlation (correlation coefficient, *r* > 0.79) for the morphological traits (e.g., seed length, width, and area) measured between the SeedGerm-VIG and *SeedGerm*, as well as a significant correlation (*r* > 0.91) for RGB color features measured by the SeedGerm-VIG and *SeedExtractor* (Supplementary Table S13). The above suggested that the SeedGerm-VIG pipeline was able to provide reliable analyses of seed size and color features compatible with results produced by methods previously reported.

### Software implementation

When training the YOlOv8x-Germ and U-Net models, a Windows 10 workstation (16 GB memory, Nvidia GTX 1660Ti GPU, and Intel Core i7-10700F CPU) was used, along with TensorFlow (V2.2) framework [68] and Python (V3.7) for the model implementation. We applied key open scientific development libraries in this study, including the scientific data processing library SciPy [69] and the image-processing library Scikit-Image [70]. All figures, except raincloud plots, were plotted using the Python libraries “matplotlib” and “seaborn” [71,72]. Line plots with confidence intervals were created by a parametric method (i.e., confidence intervals = 75%) using the “seaborn.relplot” function with parameters “kind = 'line', errorbar = ('ci', 75)”. Raincloud plots were generated using the R packages “ggplot2” and “ggdist” [73, 74]. To facilitate a broader community to access our work, we used source code and DL models of the SeedGerm-VIG together with executable Jupyter notebooks [75]. When developing the GUI of the pipeline, widget enabled by Jupyter notebook was used together with testing data to assist nonexpert users to execute the SeedGerm-VIG pipeline (Supplementary Fig. S14; Open Access).

## Availability of Source Code and Requirements

Project name: SeedGerm-VIG

Project homepage: https://github.com/The-Zhou-Lab/SeedGerm-VIG/

License: MIT license

SciCrunch RRID:SCR_027483

System requirements:

- Operating system: Windows 10/11
- Programming language: Python 3+
- Package management: see the project homepage
- Hardware requirements: NVIDIA GPU with memory ≥4 GB and system RAM ≥4 GB (minimum)

## Abbreviations

CV: computer vision; DL: deep learning; ExG: excess green; GI: germination index; GP: germination potential; GS: growth stage; GUI: graphic user interface; HD: high definition; IMB: imbibition; ISTA: International Seed Testing Association; mAP50: mean average precision at IoU = 0.5; MAS: marker-assisted selection; MGT: mean germination time; mIoU: mean intersection over union; ML: machine learning; mPA: mean pixel accuracy; PRO: protrusion; RE: radicle emergence; RG: rice genotype; RGB: red, green, and blue; ROI: region of interest; R1: root 1 (i.e., radicle); R2: root 2; R3: root 3; SE: seedling establishment; T_50_: time of 50% cumulative germination; W/L: width and length ratio.

## Data Availability

Source code, trained learning models, and algorithms of SeedGerm-VIG are distributed under the MIT license (Creative Commons Attribution 4.0 international license), permitting academic use, distribution, and reproduction in any medium. Unless otherwise stated, the Creative Commons Public Domain Dedication waiver applies to the data and results made available here [76]. The SeedVig-phase and SeedVig-traits training sets, as well as the seed germination image series (i.e., wheat, barley, and rice), are under the CC0 public waiver and are available from the BioImage Archive accession S-BIAD1852 [77]. Test data supporting the results in the article are available at the laboratory’s GitHub repository [78]. Other source code, data, and user guides are are available in the *GigaScience* repository, GigaDB [79].

## Additional Files

**Supplementary Table S1**. Experimental settings for 21 commercial wheat varieties for germination.

**Supplementary Table S2**. Descriptive statistics and broad-sense heritability of time points when radicles reached 2 mm.

**Supplementary Table S3**. Descriptive statistics and broad-sense heritability of time points when radicles reached 10 mm.

**Supplementary Table S4**. Descriptive statistics and broad-sense heritability of time points when chloroplast biogenesis was detected.

**Supplementary Table S5**. Three clusters of 21 wheat genotypes using the agglomerative clustering algorithm based on radicle growth rates, from 2 to 10 mm.

**Supplementary Table S6**. Three clusters of 21 genotypes using the affinity propagation algorithm based on time points of chloroplast biogenesis.

**Supplementary Table S7**. Three clusters of 21 genotypes using the k-means algorithm based on time points of key germination phases at the seed-lot level.

**Supplementary Table S8**. Three clusters of 21 genotypes using the affinity propagation algorithm based on time points of radicle emergence and seedling emergence at the seed-lot level.

**Supplementary Table S9**. Manual assessments of the 21 wheat genotypes for germination speed.

**Supplementary Table S10**. Coefficient of variation of the 75% data from the 3 germination speed groups based on time points of key germination phases and chloroplast biogenesis.

**Supplementary Table S11**. Interquartile range of the data from the 3 germination speed groups based on time points of key germination phases and chloroplast biogenesis.

**Supplementary Table S12**. Three clusters of 12 barley genotypes using the affinity propagation algorithm based on radicle growth rates, from 2 to 10 mm.

**Supplementary Table S13**. Correlation (*R*) of the 3 automated analytic approaches when measuring different seed parameters.

**Supplementary Note S1**. Algorithmic steps to correct seed positions and root tips tracking.

**Supplementary Note S2**. Exceptional cases when the SeedGerm-VIG made mistakes.

**Supplementary Fig. S1**. A high-quality seed germination training set for deep learning modeling.

**Supplementary Fig. S2**. Confusion matrices for evaluating the accuracy of identifying key germination phases.

**Supplementary Fig. S3**. Profile curves of wheat genotypes reaching a germination phase (15 seeds sampled from every genotype).

**Supplementary Fig. S4**. Correlation analysis between manual and computational measures of seed, root, and seedling traits across 21 wheat genotypes.

**Supplementary Fig. S5**. Correlation analysis between traditional and SeedGerm-VIG–derived traits across 21 wheat genotypes.

**Supplementary Fig. S6**. Positional changes (30–65 hours) of root tips for a given seed (row 3, column 1) from the G4 seed lot.

**Supplementary Fig. S7**. The algorithmic steps to identify roots from images with intersected roots.

**Supplementary Fig. S8**. Profile curves of radicle growth for 21 wheat genotypes during germination.

**Supplementary Fig. S9**. Profile curves of the first 3 roots for 21 wheat genotypes.

**Supplementary Fig. S10**. Raincloud plots of seed area and seed width changes during the imbibition (IMB) phase for 3 germination speed groups.

**Supplementary Fig. S11**. Root analysis of the first 3 roots in the quick, medium, and slow germination speed groups.

**Supplementary Fig. S12**. Root-based germination analysis for 12 barley genotypes.

**Supplementary Fig. S13**. Radicle emergence analysis for 6 rice genotypes.

**Supplementary Fig. S14**. The graphic user interfaces (GUIs) developed using widget in Jupyter notebooks to automate tasks in the SeedGerm-VIG pipeline.

## Supplementary Material

giaf129_Supplementary_Material

giaf129_Authors_Response_To_Reviewer_Comments_Original_Submission

giaf129_GIGA-D-25-00131_Original_Submission

giaf129_GIGA-D-25-00131_Revision_1

giaf129_Reviewer_1_Report_Original_Submission Michael Pound -- 5/11/2025

giaf129_Reviewer_1_Report_Revision_1 Michael Pound -- 9/24/2025

giaf129_Reviewer_2_Report_Original_Submission Venkat Margapuri -- 5/22/2025

giaf129_Reviewer_3_Report_Original_Submission Joseph Oddy -- 5/27/2025
