# Supplementary material for: SeedGerm-VIG: an open and comprehensive pipeline to quantify seed vigor in wheat and other cereal crops using deep learning–powered dynamic phenotypic analysis
Source: Gigascience. 2025 Oct 16;14:giaf129. doi: 10.1093/gigascience/giaf129 (PMC12648739; doi:10.1093/gigascience/giaf129)

**Supplementary Material**

## **Title:** SeedGerm-VIG: an open and comprehensive pipeline to quantify seed vigour in wheat and other cereal crops using deep learning powered dynamic phenotypic analysis

**Authors:** Jie Dai^+^, Zhenjie Wen^+^, Mujahid Ali^+^, Jinlong Huang, Shuchen Liu, Jianhua Zhao, Felipe Pinheiro, Changcai Yang, Bin Wang, Lingzhen Ye, Xueying Guan, Ji Zhou

**Supplementary Table S1.** Experimental settings for 21 commercial wheat genotypes for germination.

| **Wheat genotype ID** | **Treatment** | **Image device** | **Image sensor** | **Max image resolution** | **Seed**  **number** | **Scale**  **(pixel/mm)** |
| --- | --- | --- | --- | --- | --- | --- |
| G1 | / | *Raspberry Pi* | Sony IMX477 | 2130×1130 | 18 | 8.95 |
| G2 | / | *Raspberry Pi* | Sony IMX477 | 2130×1130 | 18 | 8.95 |
| G3 | / | *Raspberry Pi* | Sony IMX477 | 2130×1130 | 18 | 8.95 |
| G4 | cold stratification | *Raspberry Pi* | Sony IMX219 | 3280×2464 | 25 | 17.65 |
| G5 | cold stratification | *Raspberry Pi* | Sony IMX219 | 3280×2464 | 25 | 17.65 |
| G6 | cold stratification | Second-hand smartphone (SS) | Sony IMX278 | 3264×1840 | 25 | 15.88 |
| G7 | cold stratification | *Raspberry Pi* | Sony IMX219 | 3280×2464 | 25 | 17.65 |
| G8 | cold stratification | *Raspberry Pi* | Sony IMX219 | 3280×2464 | 25 | 17.65 |
| G9 | cold stratification | *Raspberry Pi* | Sony IMX219 | 3280×2464 | 25 | 17.65 |
| G10 | cold stratification | SS | Sony IMX278 | 3264×1840 | 25 | 15.88 |
| G11 | cold stratification | SS | Sony IMX278 | 3264×1840 | 25 | 15.88 |
| G12 | cold stratification | SS | Sony IMX278 | 3264×1840 | 25 | 15.88 |
| G13 | cold stratification | SS | Sony IMX278 | 3264×1840 | 25 | 15.88 |
| G14 | cold stratification | *Raspberry Pi* | Sony IMX219 | 3280×2464 | 30 | 17.65 |
| G15 | cold stratification | *Raspberry Pi* | Sony IMX219 | 3840×2160 | 30 | 17.65 |
| G16 | cold stratification | SS | Sony IMX319 | 3280×2464 | 30 | 19.41 |
| G17 | / | *Raspberry Pi* | Sony IMX708 | 4608×4608 | 20 | 11.65 |
| G18 | / | *Raspberry Pi* | Sony IMX708 | 4608×4608 | 20 | 11.90 |
| G19 | / | *Raspberry Pi* | Sony IMX708 | 4608×4608 | 20 | 11.87 |
| G20 | cold stratification | *Raspberry Pi* | Sony IMX708 | 4608×3456 | 20 | 8.71 |
| G21 | cold stratification | *Raspberry Pi* | Sony IMX708 | 4608×4608 | 20 | 12.02 |

**Supplementary Table S2.** Descriptive statistics and broad-sense heritability of timepoints when radicles reached 2 mm.

| **Genotype** | **Mean (h)** | **Std (h)** | **Min (h)** | **Max (h)** | **h^2^** |
| --- | --- | --- | --- | --- | --- |
| G1 | 59.80 | 9.78 | 44 | 76 | 0.1242 |
| G2 | 60.20 | 15.32 | 36 | 100 |  |
| G3 | 45.47 | 19.40 | 8 | 100 |  |
| G4 | 28.40 | 3.11 | 25 | 36 |  |
| G5 | 33.07 | 6.35 | 28 | 55 |  |
| G6 | 32.27 | 3.33 | 26 | 37 |  |
| G7 | 45.20 | 4.69 | 39 | 54 |  |
| G8 | 33.20 | 3.30 | 26 | 39 |  |
| G9 | 33.20 | 4.71 | 27 | 43 |  |
| G10 | 32.47 | 5.14 | 20 | 40 |  |
| G11 | 32.27 | 2.63 | 30 | 38 |  |
| G12 | 39.93 | 4.22 | 34 | 48 |  |
| G13 | 44.13 | 3.96 | 37 | 50 |  |
| G14 | 37.93 | 9.00 | 22 | 58 |  |
| G15 | 23.07 | 4.40 | 15 | 30 |  |
| G16 | 28.33 | 11.97 | 7 | 51 |  |
| G17 | 51.93 | 8.03 | 40 | 67 |  |
| G18 | 60.40 | 10.99 | 40 | 73 |  |
| G19 | 75.40 | 16.25 | 59 | 100 |  |
| G20 | 36.53 | 8.44 | 26 | 56 |  |
| G21 | 34.13 | 4.73 | 28 | 45 |  |
| Quick | 35.23 | 6.43 | 20 | 56 | 0.9495 |
| Medium | 38.20 | 9.27 | 25 | 67 |  |
| Slow | 47.09 | 20.59 | 7 | 100 |  |

**Supplementary Table S3.** Descriptive statistics and broad-sense heritability of timepoints when radicles reached 10 mm.

| **Genotype** | **Mean (h)** | **Std (h)** | **Min (h)** | **Max (h)** | **h^2^** |
| --- | --- | --- | --- | --- | --- |
| G1 | 73.73 | 13.56 | 52 | 100 | 0.4701 |
| G2 | 72.47 | 16.30 | 48 | 100 |  |
| G3 | 56.20 | 17.55 | 15 | 100 |  |
| G4 | 43.00 | 3.59 | 37 | 51 |  |
| G5 | 45.13 | 6.23 | 38 | 65 |  |
| G6 | 45.80 | 4.13 | 38 | 54 |  |
| G7 | 58.60 | 5.07 | 52 | 67 |  |
| G8 | 47.33 | 3.56 | 38 | 52 |  |
| G9 | 51.00 | 6.92 | 42 | 65 |  |
| G10 | 46.07 | 5.48 | 36 | 57 |  |
| G11 | 47.07 | 3.08 | 42 | 53 |  |
| G12 | 51.80 | 5.09 | 45 | 61 |  |
| G13 | 58.27 | 5.28 | 46 | 68 |  |
| G14 | 52.60 | 8.48 | 36 | 71 |  |
| G15 | 38.53 | 4.67 | 31 | 46 |  |
| G16 | 41.40 | 11.27 | 25 | 64 |  |
| G17 | 64.60 | 8.03 | 53 | 80 |  |
| G18 | 78.67 | 17.42 | 54 | 100 |  |
| G19 | 88.87 | 12.40 | 73 | 100 |  |
| G20 | 53.33 | 20.72 | 34 | 100 |  |
| G21 | 45.87 | 4.70 | 36 | 52 |  |
| Quick | 48.44 | 10.73 | 34 | 100 | 0.9534 |
| Medium | 52.10 | 9.03 | 37 | 80 |  |
| Slow | 61.50 | 20.75 | 15 | 100 |  |

**Supplementary Table S4.** Descriptive statistics and broad-sense heritability of timepoints when chloroplast biogenesis was detected.

| **Genotype** | **Mean (h)** | **Std (h)** | **Min (h)** | **Max (h)** | **h^2^** |
| --- | --- | --- | --- | --- | --- |
| G1 | 87.27 | 19.20 | 48 | 100 | 0.3165 |
| G2 | 84.07 | 18.43 | 51 | 100 |  |
| G3 | 81.33 | 22.00 | 49 | 100 |  |
| G4 | 46.20 | 9.03 | 33 | 64 |  |
| G5 | 47.67 | 8.85 | 36 | 68 |  |
| G6 | 48.13 | 6.23 | 42 | 65 |  |
| G7 | 54.07 | 7.44 | 38 | 67 |  |
| G8 | 50.27 | 6.23 | 35 | 59 |  |
| G9 | 49.27 | 17.78 | 27 | 100 |  |
| G10 | 49.60 | 8.24 | 35 | 61 |  |
| G11 | 51.87 | 14.52 | 39 | 100 |  |
| G12 | 54.40 | 15.79 | 39 | 100 |  |
| G13 | 62.27 | 12.90 | 48 | 100 |  |
| G14 | 48.60 | 12.84 | 25 | 75 |  |
| G15 | 48.60 | 7.63 | 39 | 66 |  |
| G16 | 51.67 | 11.34 | 35 | 77 |  |
| G17 | 65.87 | 16.66 | 44 | 100 |  |
| G18 | 86.20 | 16.81 | 49 | 100 |  |
| G19 | 82.27 | 23.46 | 19 | 100 |  |
| G20 | 51.53 | 13.26 | 31 | 73 |  |
| G21 | 64.80 | 21.69 | 36 | 100 |  |
| Quick | 50.14 | 11.21 | 25 | 100 | 0.9934 |
| Medium | 64.31 | 17.13 | 36 | 100 |  |
| Slow | 84.23 | 19.70 | 19 | 100 |  |

**Supplementary Table S5.** Three clusters of 21 wheat genotypes using the agglomerative clustering algorithm based on radicle growth rates, from 2 mm to 10 mm.

| **Genotypes** | **Growth rate (2-10 mm; mm/h)** | **Group** |
| --- | --- | --- |
| G1 | 0.480228 | Slow |
| G2 | 0.501862 | Slow |
| G3 | 0.446927 | Slow |
| G4 | 0.547526 | Medium |
| G5 | 0.609675 | Quick |
| G6 | 0.579859 | Medium |
| G7 | 0.581238 | Medium |
| G8 | 0.557492 | Medium |
| G9 | 0.482222 | Slow |
| G10 | 0.598448 | Quick |
| G11 | 0.545679 | Medium |
| G12 | 0.696091 | Quick |
| G13 | 0.567866 | Medium |
| G14 | 0.459111 | Slow |
| G15 | 0.507167 | Slow |
| G16 | 0.456386 | Slow |
| G17 | 0.573064 | Medium |
| G18 | 0.469270 | Slow |
| G19 | 0.422526 | Slow |
| G20 | 0.645294 | Quick |
| G21 | 0.617774 | Quick |

**Supplementary Table S6.** Three clusters of 21 genotypes using the affinity propagation algorithm based on timepoints of chloroplast biogenesis.

| **Genotypes** | **Chloroplast biogenesis (hr.)** | **Group** |
| --- | --- | --- |
| G1 | 80.6 | Slow |
| G2 | 78.7 | Slow |
| G3 | 76.0 | Slow |
| G4 | 46.2 | Quick |
| G5 | 47.7 | Quick |
| G6 | 48.1 | Quick |
| G7 | 54.1 | Quick |
| G8 | 50.3 | Quick |
| G9 | 48.6 | Quick |
| G10 | 49.6 | Quick |
| G11 | 51.2 | Quick |
| G12 | 53.7 | Quick |
| G13 | 61.6 | Medium |
| G14 | 48.6 | Quick |
| G15 | 48.6 | Quick |
| G16 | 51.7 | Quick |
| G17 | 64.5 | Medium |
| G18 | 80.9 | Slow |
| G19 | 76.9 | Slow |
| G20 | 51.5 | Quick |
| G21 | 62.8 | Medium |

**Supplementary Table S7.** Three clusters of 21 genotypes using the K-means algorithm based on timepoints of key germination phases at the seed-lot level.

| **Genotypes** | **PRO (hr.)** | **RE (hr.)** | **SE (hr.)** | **Group** |
| --- | --- | --- | --- | --- |
| G1 | 47 | 67 | 85 | Slow |
| G2 | 51 | 62 | 78 | Slow |
| G3 | 42 | 50 | 66 | Medium |
| G4 | 26 | 35 | 51 | Quick |
| G5 | 29 | 37 | 49 | Quick |
| G6 | 1 | 38 | 48 | Quick^a^ |
| G7 | 41 | 52 | 63 | Medium |
| G8 | 28 | 36 | 54 | Quick |
| G9 | 29 | 36 | 57 | Quick |
| G10 | 25 | 38 | 49 | Quick^a^ |
| G11 | 28 | 37 | 50 | Quick |
| G12 | 38 | 39 | 54 | Quick |
| G13 | 22 | 53 | 61 | Medium^a^ |
| G14 | 38 | 46 | 71 | Median |
| G15 | 23 | 29 | 50 | Quick |
| G16 | 18 | 39 | 51 | Quick^a^ |
| G17 | 47 | 55 | 71 | Medium |
| G18 | 61 | 69 | 76 | Slow |
| G19 | 66 | 78 | 85 | Slow |
| G20 | 31 | 33 | 47 | Quick |
| G21 | 28 | 36 | 45 | Quick |

^a^ Predicted by K-means after model construction because the PRO timepoints were affected by the illuminance.

**Supplementary Table S8.** Three clusters of 21 genotypes using the affinity propagation algorithm based on timepoints of radicle emergence and seedling emergence at the seed-lot level.

| **Genotypes** | **RE (hr.)** | **SE (hr.)** | **Group** |
| --- | --- | --- | --- |
| G1 | 67 | 85 | Slow |
| G2 | 62 | 78 | Slow |
| G3 | 50 | 66 | Medium |
| G4 | 35 | 51 | Quick |
| G5 | 37 | 49 | Quick |
| G6 | 38 | 48 | Quick |
| G7 | 52 | 63 | Medium |
| G8 | 36 | 54 | Quick |
| G9 | 36 | 57 | Quick |
| G10 | 38 | 49 | Quick |
| G11 | 37 | 50 | Quick |
| G12 | 39 | 54 | Quick |
| G13 | 53 | 61 | Medium |
| G14 | 46 | 71 | Medium |
| G15 | 29 | 50 | Quick |
| G16 | 39 | 51 | Quick |
| G17 | 55 | 71 | Medium |
| G18 | 69 | 76 | Slow |
| G19 | 78 | 85 | Slow |
| G20 | 33 | 47 | Quick |
| G21 | 36 | 45 | Quick |

**Supplementary Table S9.** Manual assessments of the 21 wheat genotypes for germination speed.

| **Genotypes** | **Group** |
| --- | --- |
| G1 | Slow |
| G2 | Slow |
| G3 | Slow |
| G4 | Quick |
| G5 | Quick |
| G6 | Quick |
| G7 | Quick |
| G8 | Quick |
| G9 | Quick |
| G10 | Quick |
| G11 | Quick |
| G12 | Quick |
| G13 | Quick |
| G14 | Quick |
| G15 | Quick |
| G16 | Quick |
| G17 | Quick |
| G18 | Slow |
| G19 | Slow |
| G20 | Quick |
| G21 | Quick |

**Supplementary Table S10.** Coefficient of variation of the 75% data from the three germination speed groups based on timepoints of key germination phases and chloroplast biogenesis.

| **Type** | **Quick** | **Medium** | **Slow** |
| --- | --- | --- | --- |
| Timepoints when radicles reached 2 mm | 10.19 | 17.20 | 29.45 |
| Timepoints when radicles reached 10 mm | 7.77 | 12.04 | 18.76 |
| Timepoints of chloroplast biogenesis | 11.30 | 13.14 | 11.86 |

**Supplementary Table S11.** Interquartile range of the data from the three germination speed groups based on timepoints of key germination phases and chloroplast biogenesis.

| **Type** | **Quick** | **Medium** | **Slow** |
| --- | --- | --- | --- |
| Timepoints when radicles reached 2 mm | 14.0 | 7.5 | 31.5 |
| Timepoints when radicles reached 10 mm | 12.0 | 8.0 | 29.0 |
| Timepoints of chloroplast biogenesis | 18.0 | 11.5 | 31.0 |

**Supplementary Table S12.** Three clusters of 12 barley genotypes using the affinity propagation algorithm based on radicle growth rates, from 2 mm to 10 mm.

| **Genotypes** | **Growth rate (2-10 mm; mm/h)** | **Group** |
| --- | --- | --- |
| BG1 | 0.251829 | Slow |
| BG2 | 0.297778 | Slow |
| BG3 | 0.377966 | Medium |
| BG4 | 0.340633 | Medium |
| BG5 | 0.366930 | Medium |
| BG6 | 0.414906 | Medium |
| BG7 | 0.472306 | Quick |
| BG8 | 0.282509 | Slow |
| BG9 | 0.536722 | Quick |
| BG10 | 0.614303 | Quick |
| BG11 | 0.190823 | Slow |
| BG12 | 0.399567 | Medium |

**Supplementary Table S13.** Correlation (*R*) of the three automated analytic approaches when measuring different seed parameters.

| **Trait** | **SeedExtractor vs SeedGerm** | **SeedExtractor vs SeedGerm-VIG** | **SeedGerm vs SeedGerm-VIG** |
| --- | --- | --- | --- |
| Length | 0.7801 | 0.6590 | 0.8690 |
| Width | 0.7731 | 0.6073 | 0.8163 |
| Area | 0.7825 | 0.5863 | 0.8583 |
| Perimeter | 0.7319 | 0.5299 | 0.7990 |
| R channel | / | 0.6719 | / |
| G channel | / | 0.9117 | / |
| B channel | / | 0.9599 | / |

**Supplementary Note S1.** Algorithmic steps to correct seed positions and root tips tracking.

Due to missed or overlapping detections in YOLOv8x results, a correction algorithm was developed. First, seed-level masks without roots and seedling (hereafter as ‘seed’) and seeds with roots and seedling (hereafter as ‘overall’) predicted by the trained U-net model and the bounding boxes detected by the YOLOv8x model were obtained for each image. Then, the seed objects were numbered according to the sorted centroid coordinates of seeds in the first image. The bounding boxes (confidence level ≥ 0.5) were assigned to each seed object based on the overlap between seed region and bounding box region. The seed objects were continuously tracked based on the intersection of seed masks in two consecutive images. The masks of overall were then used to determine whether seed object overlap based on the number of overlapping seed objects within connected regions.

When no seed objects intersected, the bounding box of each numbered seed object was updated accordingly. If multiple bounding boxes overlapped with the same seed object, only the bounding box with the highest confidence level was retained. The bounding box of one seed object was updated if the pixel area of the overall mask within the bounding box accounted for at least 85% of the total pixel area of the mask.

When seed objects intersected, after updating the bounding box of non-intersected seed objects, among the remaining bounding boxes, only bounding boxes that overlap with the seed mask by at least 85% were retained. If there was only one seed mask within the bounding box, bounding box with the highest overlap rate (calculated with previous bounding box) was selected. If there were still seed objects whose bounding boxes had not been updated, bounding box updating operations were repeated without the restriction of the number of seed masks within them. Seed objects whose bounding boxes weren’t updated retained their previous bounding boxes.

**Supplementary Note S2.** Exceptional cases that the SeedGerm-VIG made mistakes.

When seeds or roots move, the quantification of lateral root length, especially for the second and third roots (not radicles), could be inaccurate (R² = ~0.4; see **Fig. S4**). As shown in the figure below, suboptimal imaging conditions (e.g., water reflection). extensive seed or root movements, and complex or occluded root systems were likely leading to: (1) impossible to track root tips (row 1; red-coloured outlines showing seed area in the two consecutive images); (2) wrongly identified root tips (row 2); (3) wrongly segmented masks (row 3; although removable through image processing); (4) incomplete seed area (row 4).


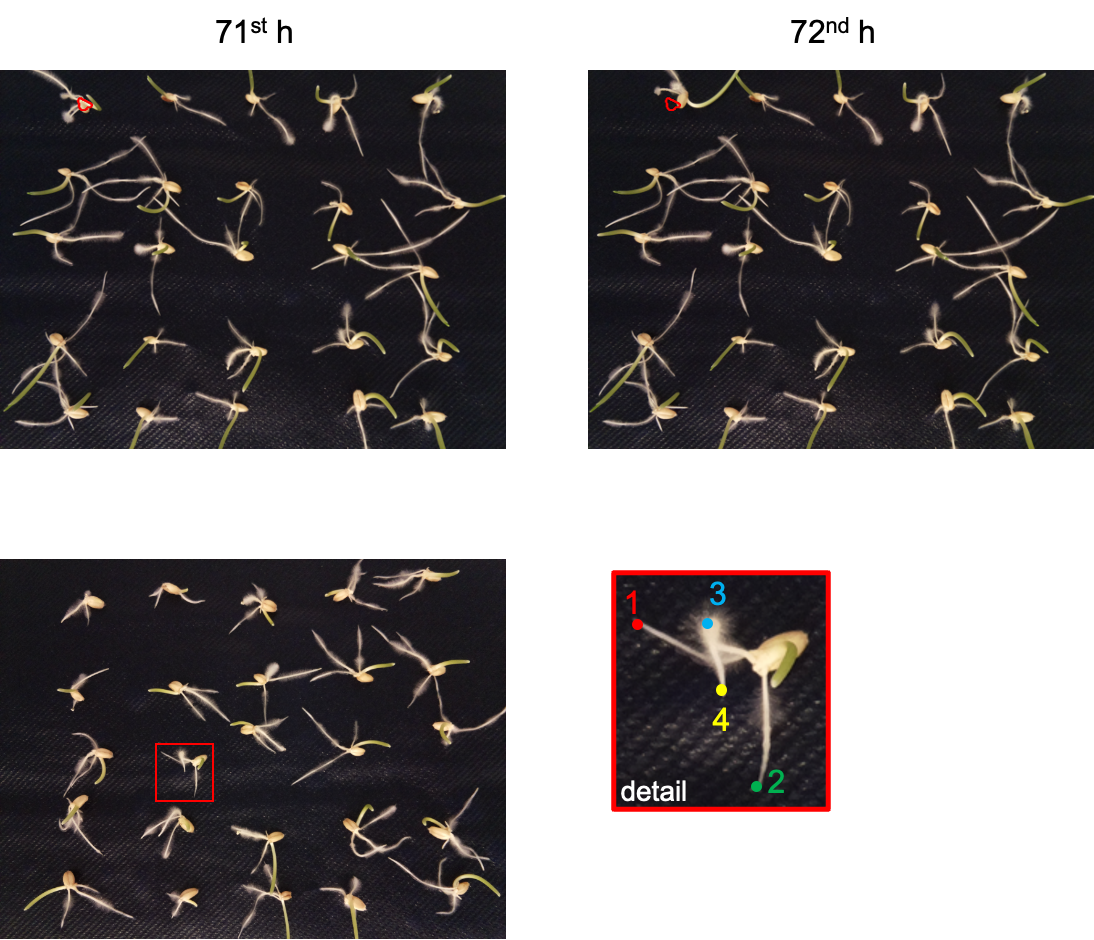


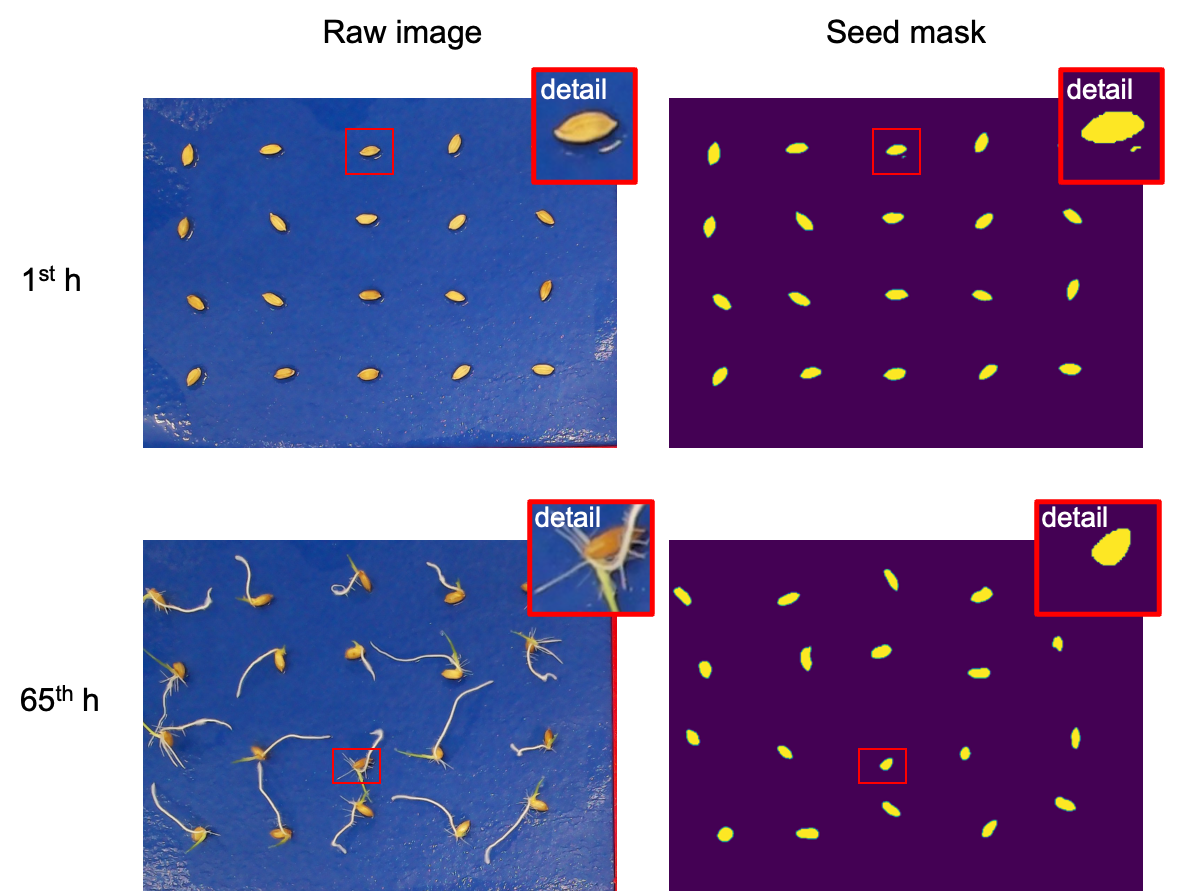


**Supplementary Figure S1.** A high-quality seed germination training set for deep learning modelling.


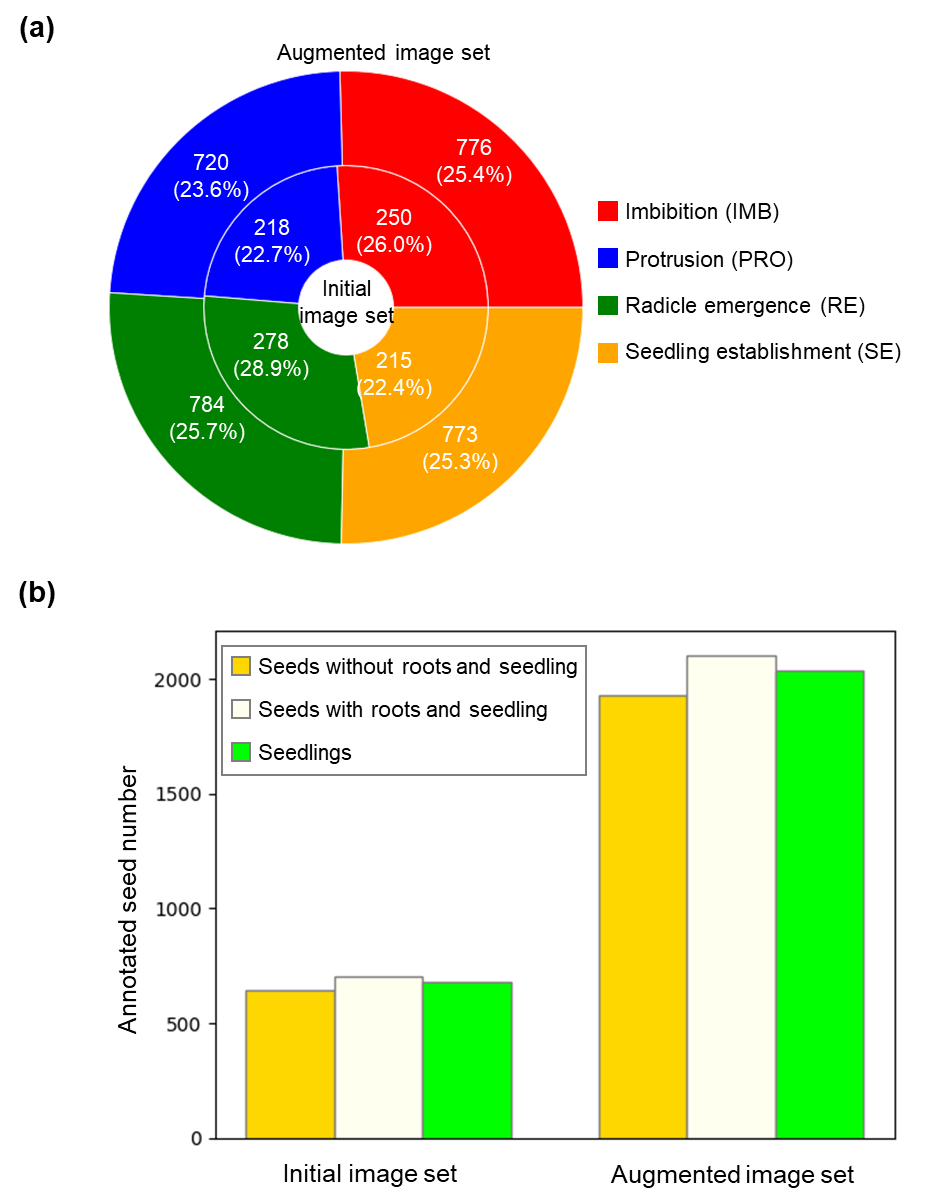


(a) The ‘SeedVig-phase’ dataset for the YOLOv8x model with inner region indicating the initial image set while outer region the augmented image set. (b) The ‘SeedVig-traits’ dataset for the U-net architecture.

**Supplementary Figure S2.** Confusion matrixes for evaluating the accuracy of identifying key germination phases.


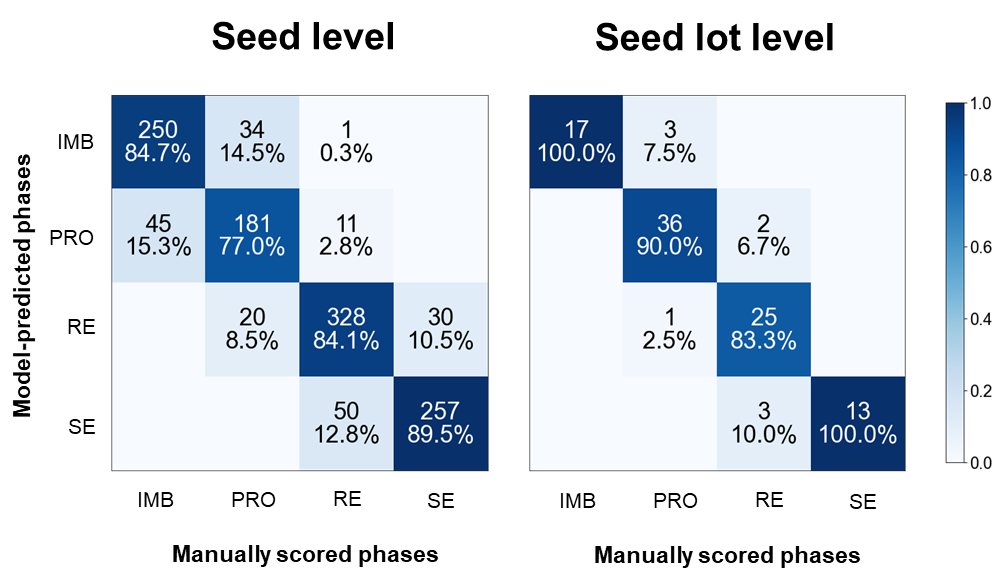


**Supplementary Figure S3.** Profile curves of wheat genotypes reaching a germination phase (15 seeds sampled from every genotype).


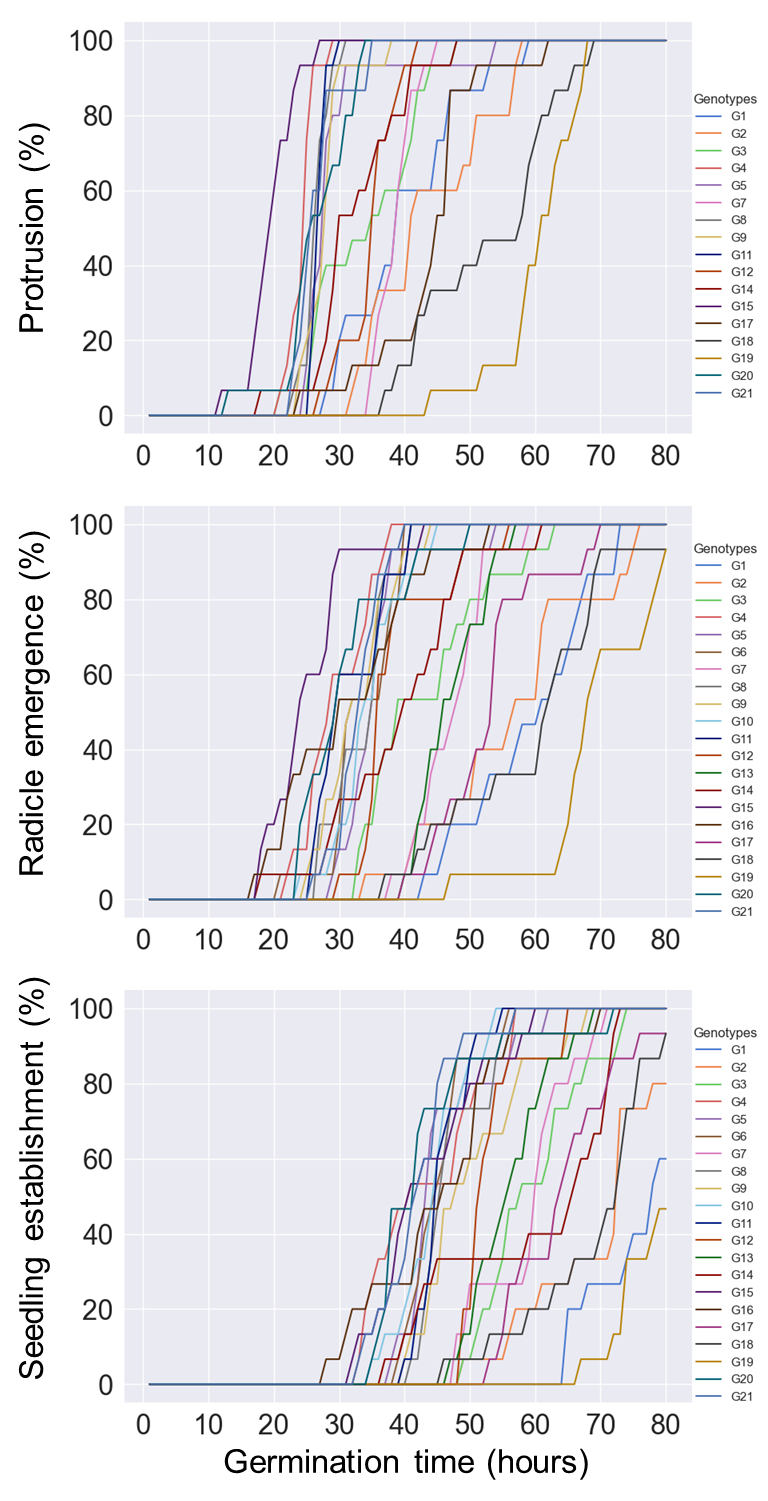


**Supplementary Figure S4.** Correlation analysis between manual and computational measures of seed, root, and seedling traits across 21 wheat genotypes.


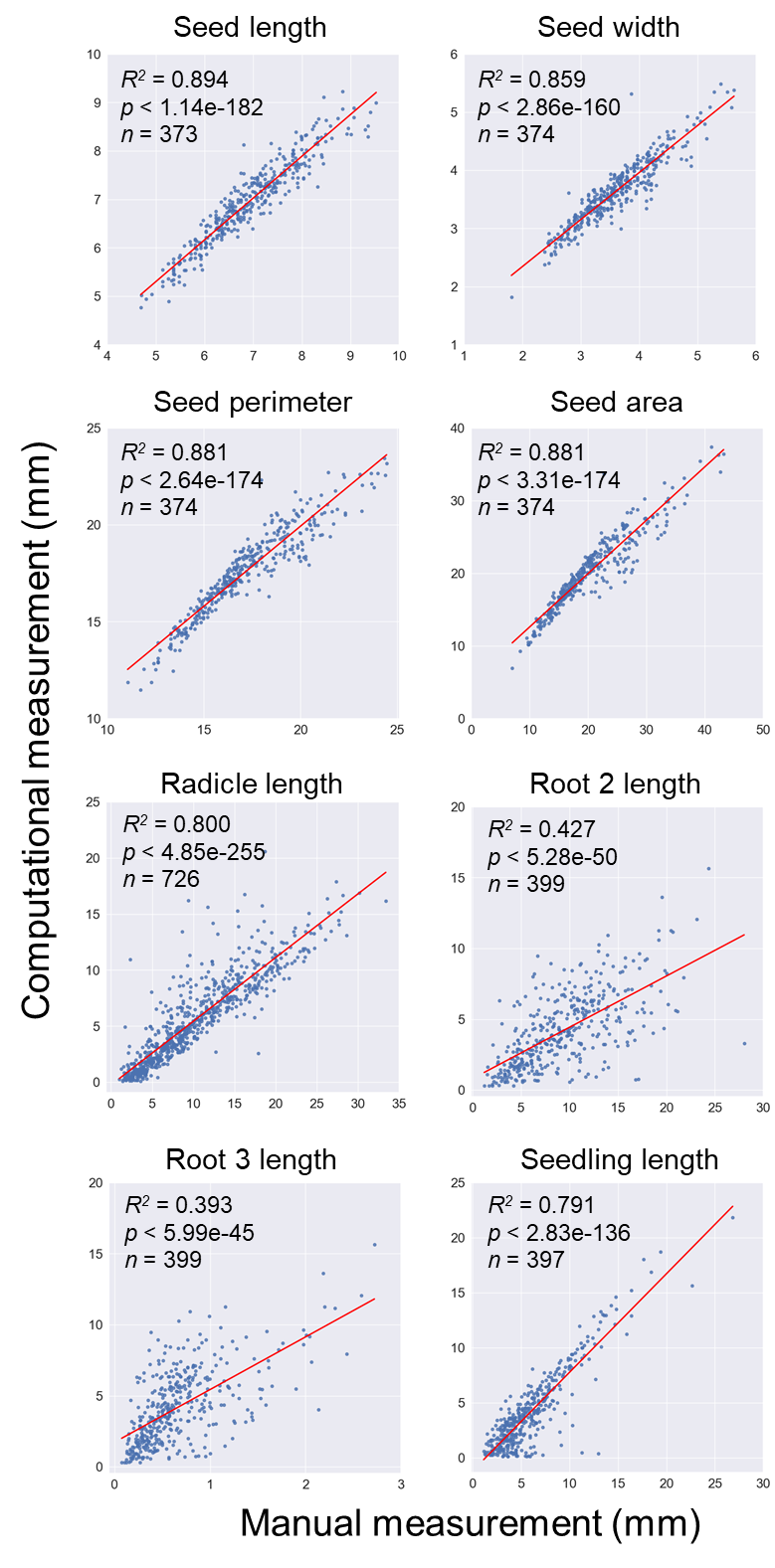


**Note**: the red lines represent the line of best fit, along the *R^2^* value and *p*-value indicating the strength and significance of the correlations, while *n* representing the number of observations.

**Supplementary Figure S5.** Correlation analysis between traditional and SeedGerm-VIG derived traits across 21 wheat genotypes.


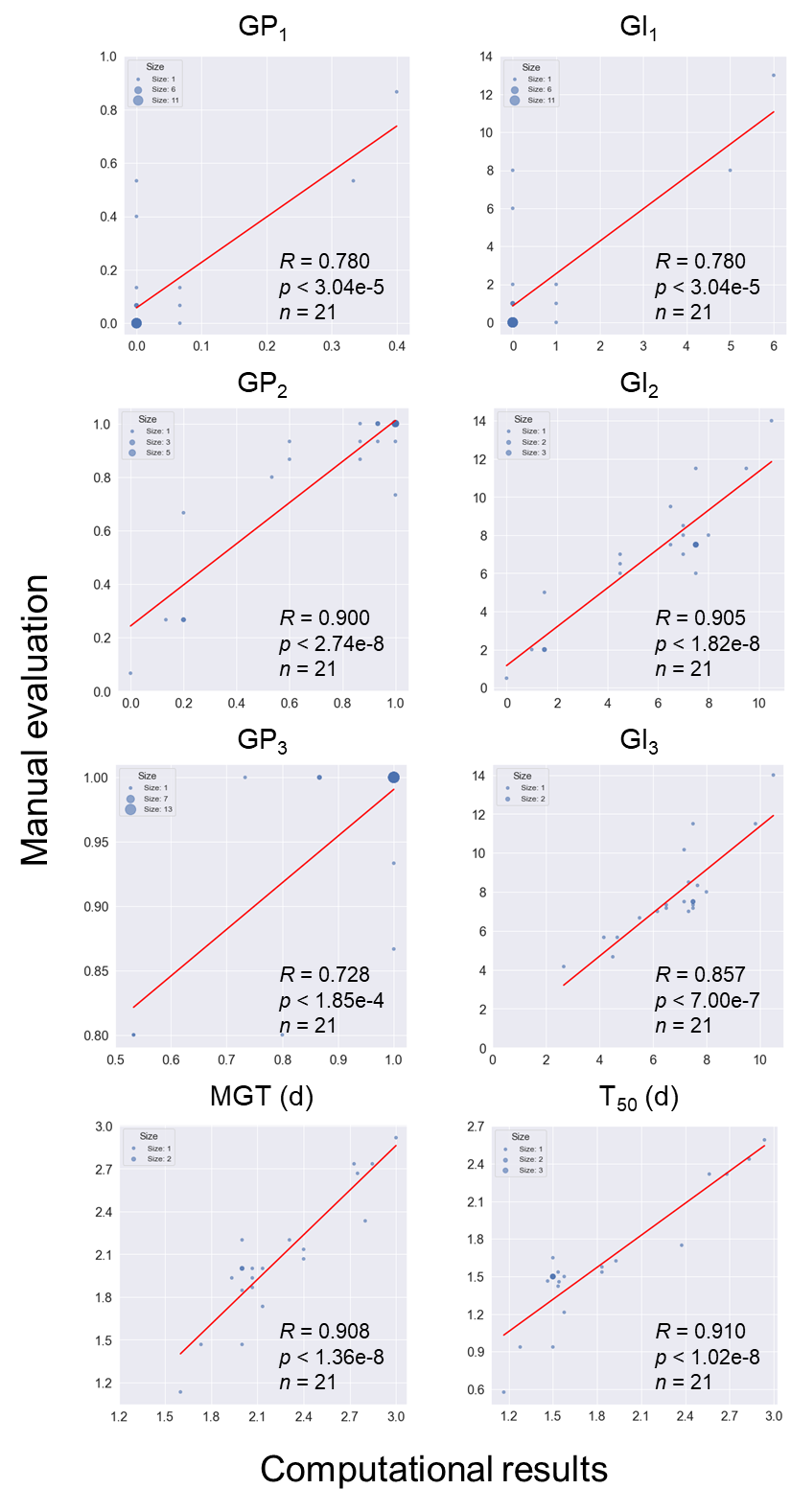


**Supplementary Figure S6.** Positional changes (30-65 hours) of root tips for a given seed (Row 3, Column 1) from the G4 seed lot.


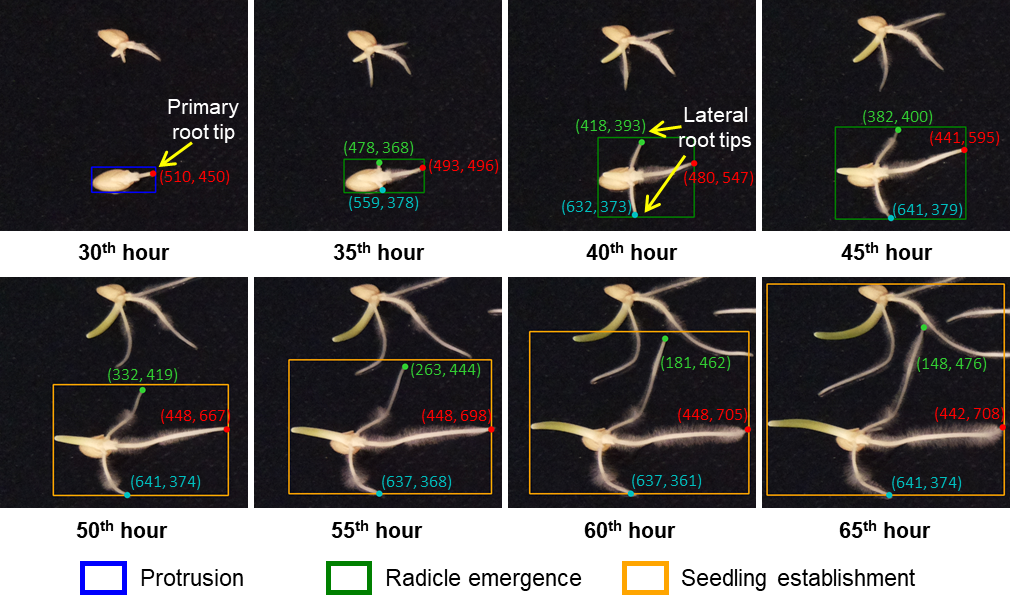


Coordinates of radicle tips were displayed for each timepoint to quantify positional changes.

**Supplementary Figure S7.** The algorithmic steps to identify roots from images with intersected roots.


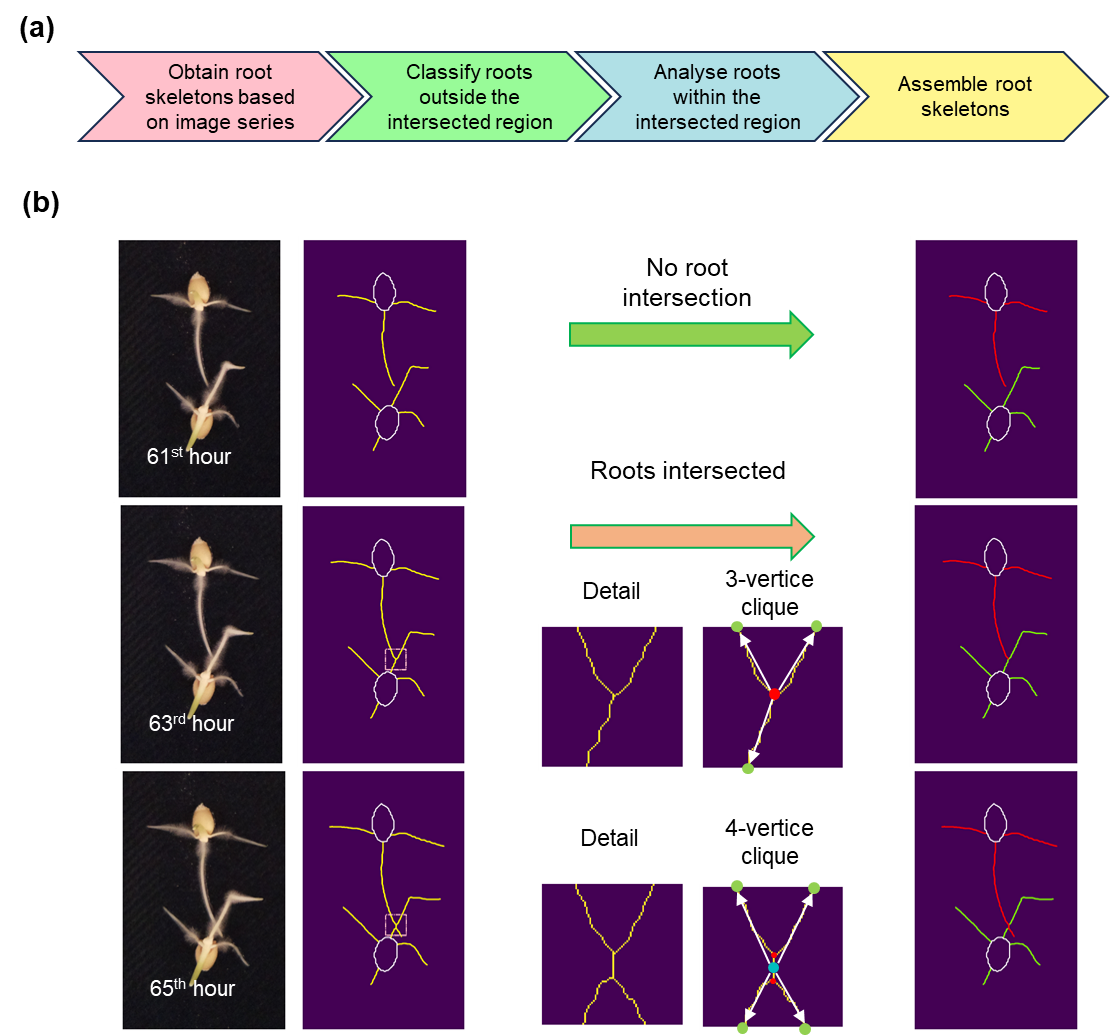


(a) A general workflow for segmenting intersected roots. (b) Progression of root segmentation for selected seeds in 3-vertice and 4-vertice cliques based on vectors from 61^st^ hour to 65^th^ hour.

**Supplementary Figure S8.** Profile curves of radicle growth for 21 wheat genotypes during germination.


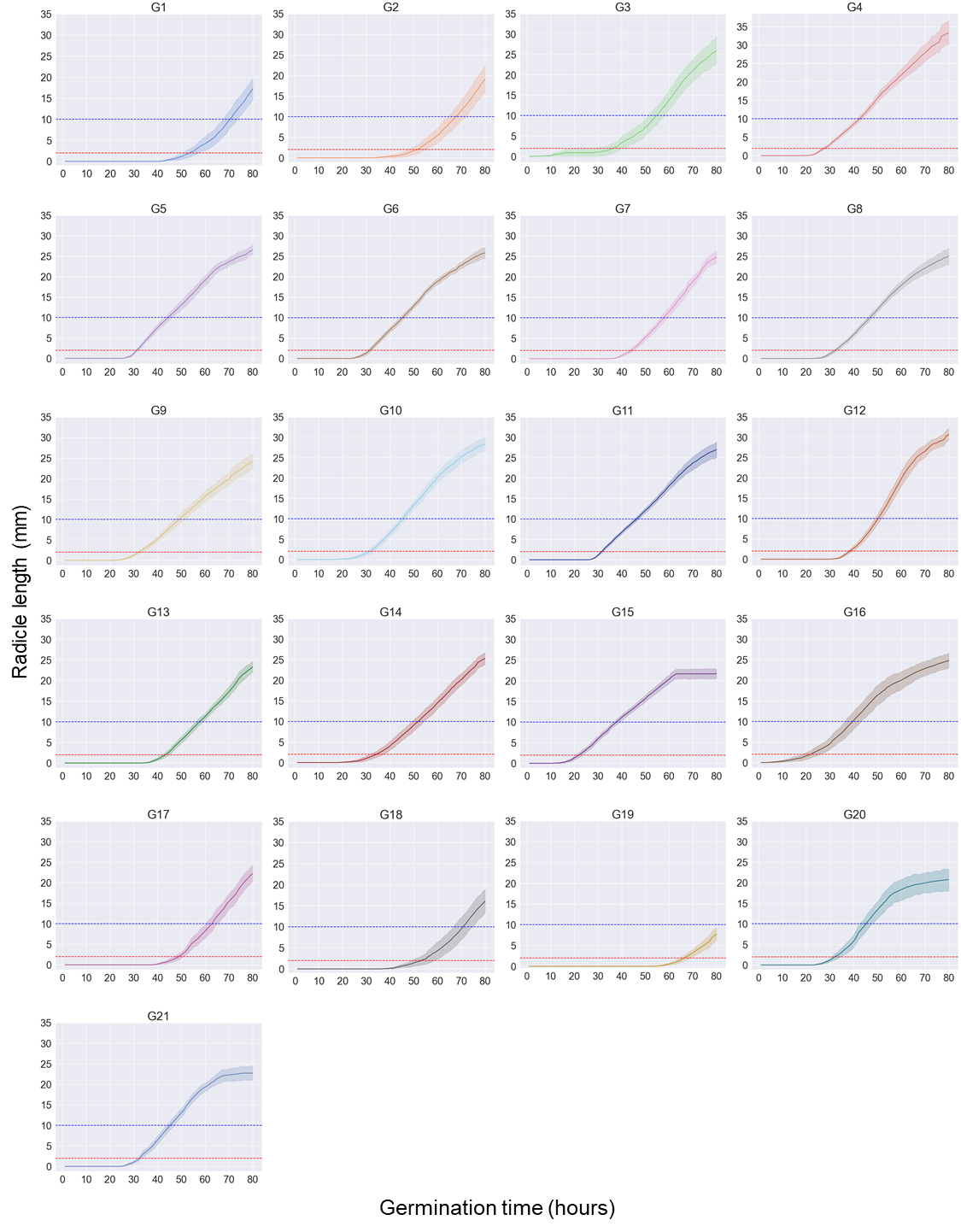


Red dotted line: 2 mm, RE criterion of ISTA; Blue dotted line: 10 mm, root establishment criterion for treatment or stress response.

**Supplementary Figure S9.** Profile curves of first three roots for 21 wheat genotypes.


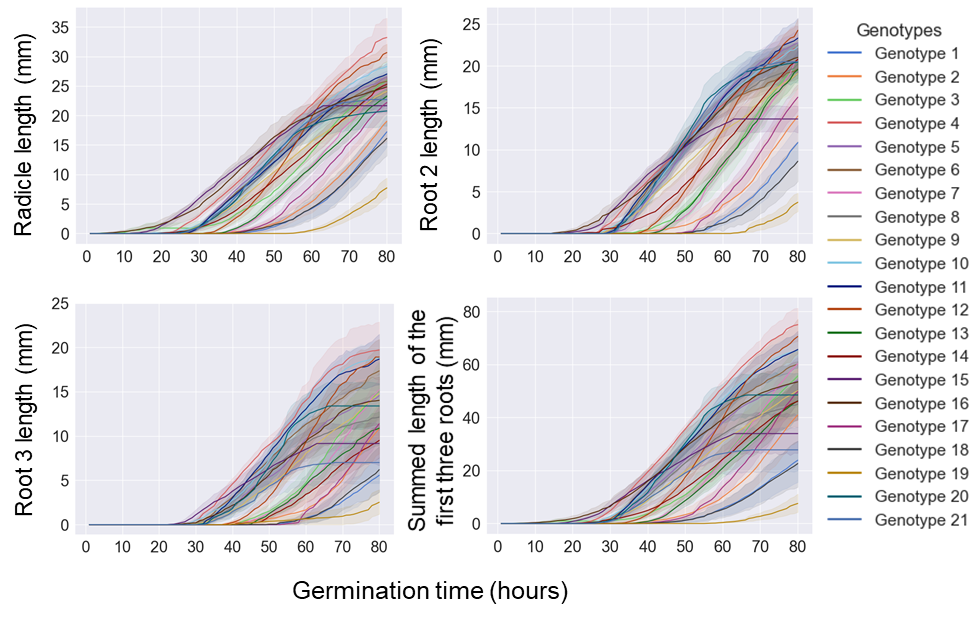


**Supplementary Figure S10.** Raincloud plots of seed area and seed width changes during the imbibition (IMB) phase for three germination speed groups.


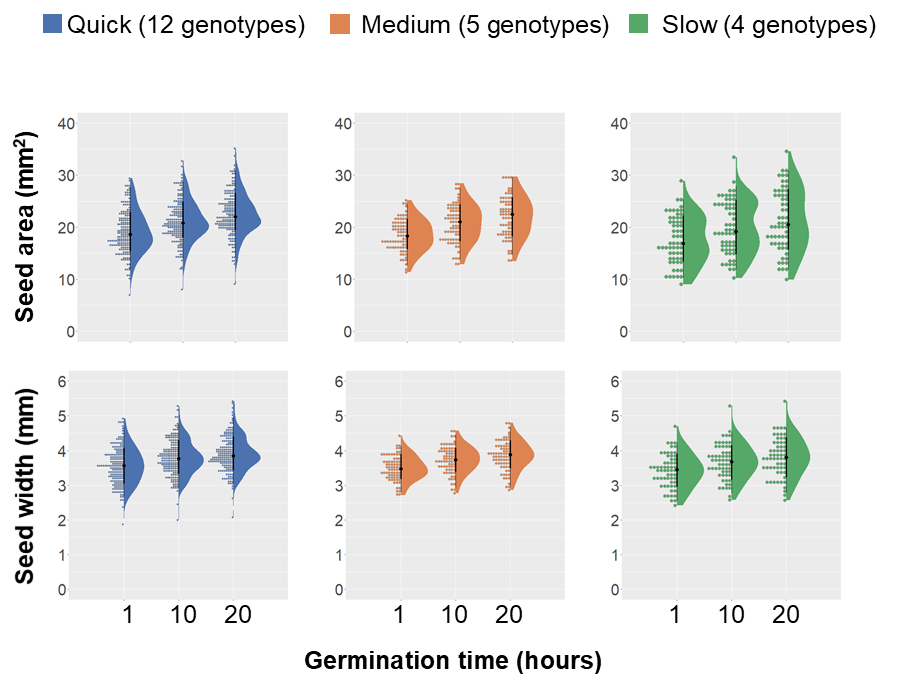


Note: three groups of wheat genotypes were categorized by germination speed, which are explained in **Table S4**.

**Supplementary Figure S11.** Root analysis of the first three roots in the Quick, Medium, and Slow germination speed groups.


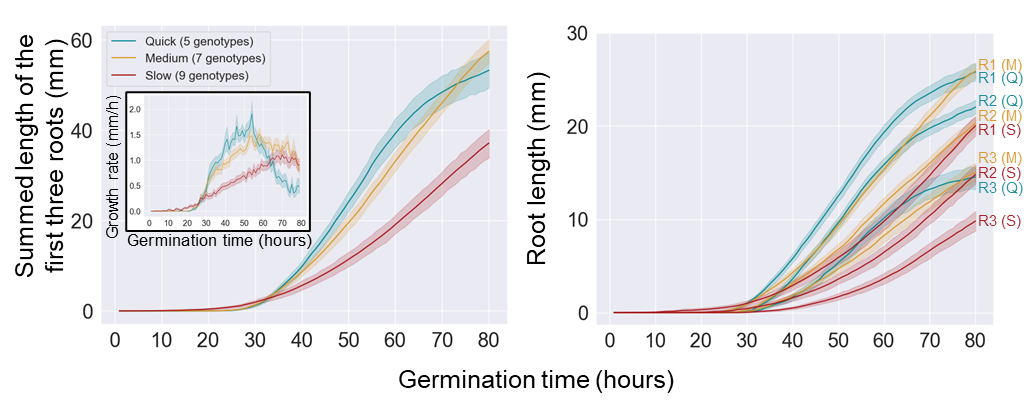


(**Left**) Profile curves showing the summed length of the first three roots for each group, with an inset detailing their growth rate changes over time. (**Right**) Individual profile curves of the first three roots across the three groups, where R1 represented the radicle (primary root), and R2 and R3 represented lateral roots.

**Supplementary Figure S12.** Root-based germination analysis for twelve barley genotypes.


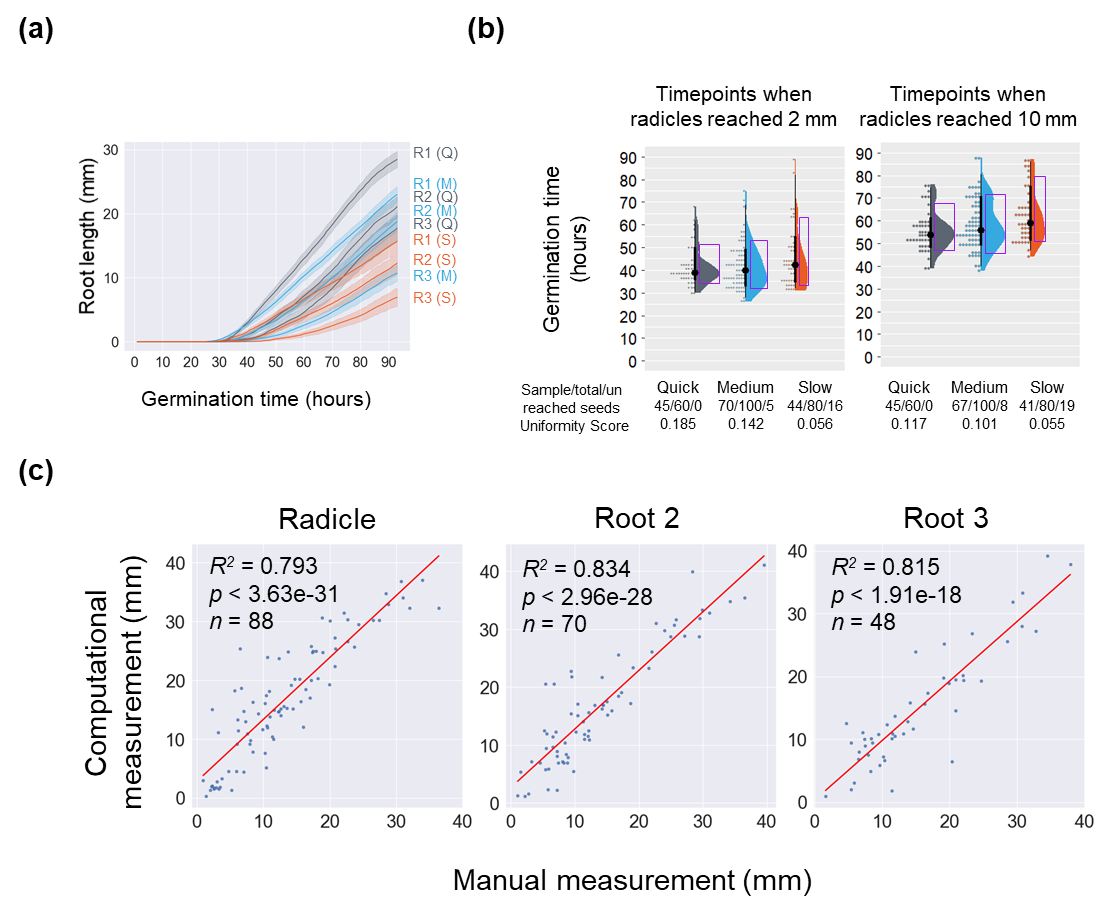


(**a**) Profile curves of each root length over time for twelve barley genotypes which were classified into Quick, Medium, and Slow Speed groups. (**b**) Raincloud plot showing how uniform radicles in the three Speed groups reached 2 mm and 10 mm. Sampled, total and unreached seeds were listed under each group. Uniformity scores were provided based on 75% of the data. (**c**) Correlations between manual and computational measurements of radicles, Root 2 and Root 3.

**Supplementary Figure S13.** Radicle emergence analysis for six rice genotypes.


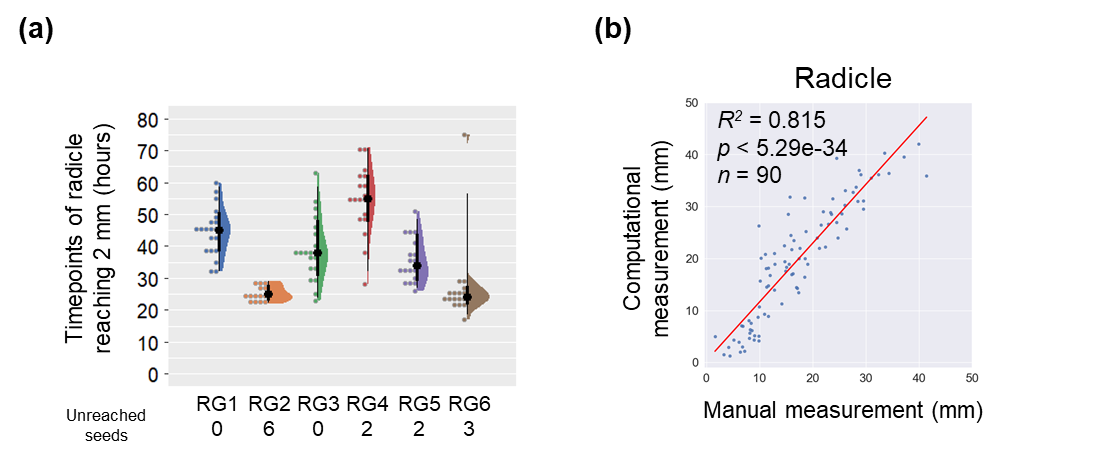


(**a**) Raincloud plot showing timepoints when radicle lengths reached 2 mm for three rice genotypes. Unreached seeds number was listed under indicated genotypes. (**b**) Correlations between manual and computational measurements of radicle lengths.

**Supplementary Figure S14.** The graphic user interfaces developed using widget in Jupyter notebooks to automate tasks in the SeedGerm-VIG pipeline.


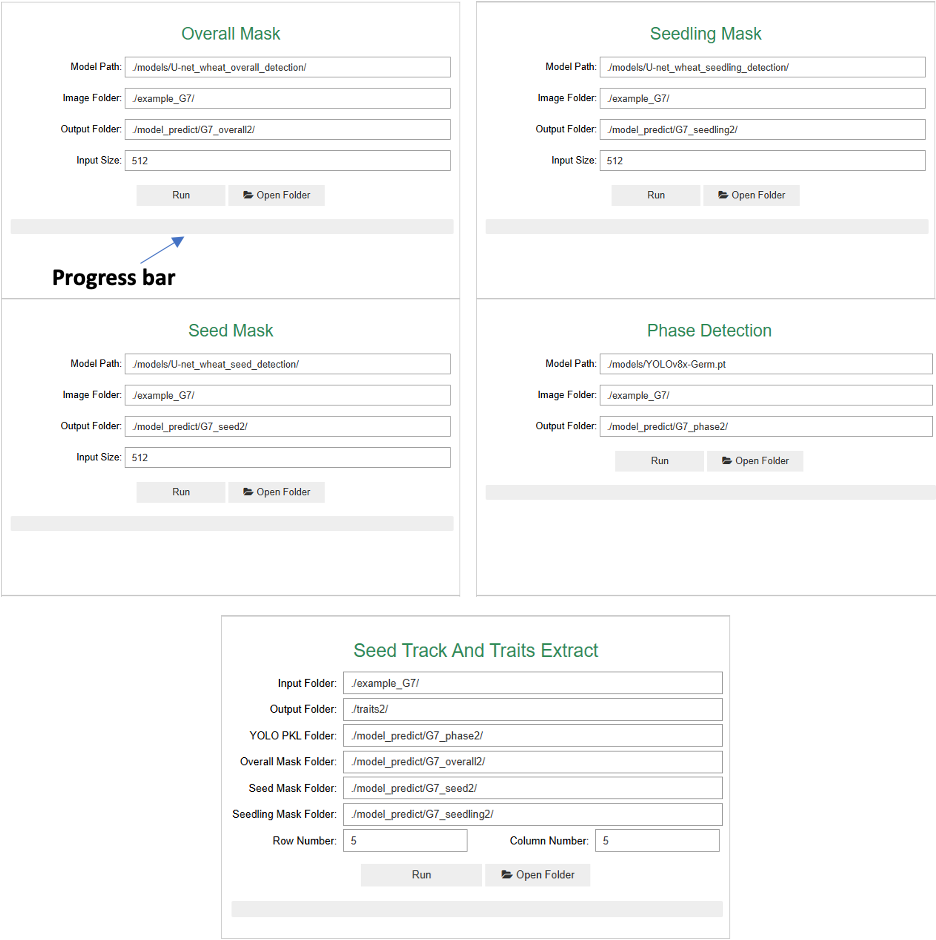

Supplement: giaf129_Supplementary_Material [file giaf129_supplementary_material.docx]
