# Supplementary material for: SeedGerm-VIG: an open and comprehensive pipeline to quantify seed vigor in wheat and other cereal crops using deep learning–powered dynamic phenotypic analysis
Source: Gigascience. 2025 Oct 16;14:giaf129. doi: 10.1093/gigascience/giaf129 (PMC12648739; doi:10.1093/gigascience/giaf129)
Supplement: giaf129_GIGA-D-25-00131_Revision_1 [file giaf129_giga-d-25-00131_revision_1.pdf]

GigaScience

SeedGerm-VIG: an open and comprehensive pipeline to quantify seed vigour in wheat and other cereal crops using deep learning powered dynamic phenotypic analysis

--Manuscript Draft--

**Manuscript Number:**	GIGA-D-25-00131R1
**Full Title:**	SeedGerm-VIG: an open and comprehensive pipeline to quantify seed vigour in wheat and other cereal crops using deep learning powered dynamic phenotypic analysis
**Article Type:**	Research
**Funding Information:**	National Natural Science Foundation of China (32070400) — Prof Ji Zhou<br>National Natural Science Foundation of China (U24A20402) — Not applicable<br>Gray Foundation (G118688) — Prof Ji Zhou<br>Biotechnology and Biological Sciences Research Council (BB/Y513969/1) — Prof Ji Zhou<br>Biotechnology and Biological Sciences Research Council (BB/Y514081/1) — Not applicable<br>Biotechnology and Biological Sciences Research Council (BB/X019683/1) — Not applicable
**Abstract:**	As one of the most important cereal crops, wheat (Triticum aestivum L.) production and grain quality are essential to many nations in the world. Early developmental phases such as seed germination and seedling establishment are key to wheat's growth and development as they impact directly on crop's early performance and yield potential. Hence, it is critical to develop varieties with favourable early growth characteristics under various growing conditions. Here, we present SeedGerm-VIG, an automated and comprehensive pipeline developed for assessing seed vigour in wheat and other cereal crops. Building on the SeedGerm system, we integrated multiple deep learning models (i.e. YOLOv8x-Germ and optimised U-Net) and computer vision algorithms into the automated seed-level analysis pipeline to identify key germination phases and measure seed-, root-, and seedling-level phenotypic traits. Then, by using time series directed graph, not only did we track root tips to measure root emergence during the germination procedure (seed-lot R2 = 84.1%), but we also established a new approach to examine speed and uniformity of germination. These resulted in the establishment of a vigour scoring matrix, through which 21 commercial genotypes' (i.e. 494 randomly sampled seeds, with over 29,500 seed-level images) vigour scores were summarised and evaluated at key phases such as protrusion, radicle emergence, and chloroplast biogenesis, which largely matched with manual assessment based on the International Seed Testing Association (ISTA) guidelines. Finally, we also demonstrated that the SeedGerm-VIG pipeline could be used to assess seed vigour for other cereal crops such as rice (n = 120 seeds) and barley (n = 240 seeds), reliably. In conclusion, we believe that our work demonstrates a valuable step forward to enable the broader plant and crop research community to examine seed vigour and vigour-related features in an automated manner, facilitating effective and reproducible plant selection and relevant seed science research for crop improvement.
**Corresponding Author:**	Ji Zhou<br>NIAB: National Institute of Agricultural Botany<br>Cambridge, UNITED KINGDOM
**Corresponding Author Secondary Information:**	
**Corresponding Author's Institution:**	NIAB: National Institute of Agricultural Botany
**Corresponding Author's Secondary Institution:**	

Powered by Editorial Manager® and ProduXion Manager® from Aries Systems Corporation

**First Author:**	Jie Dai
**First Author Secondary Information:**	
**Order of Authors:**	Jie Dai
	Zhenjie Wen
	Mujahid Ali
	Shuchen Liu
	Jianhua Zhao
	Felipe Pinheiro
	Changcai Yang
	Bin Wang
	Lingzhen Ye
	Xueying Guan
	Ji Zhou
	Jinlong Huang
**Order of Authors Secondary Information:**	
**Response to Reviewers:**	Editor's comments<br>Please register any new software application in the bio.tools and SciCrunch.org databases to receive RRID (Research Resource Identification Initiative ID) and biotoolsID identifiers, and include these in your manuscript. Computational workflows should be registered in workflowhub.eu and the DOIs cited in the relevant places in the manuscript. These will facilitate tracking, reproducibility and re-use of your tool.<br>Response: Thanks for your suggestions. We have created a GUI using widget enabled by Jupyter notebook to facilitate non-expert accessing the SeedGerm-VIG (Lines 620-622 and Lines 238-241; Fig. S14). As we did not package the Jupyter notebook due to different runtime environments that different end users with varied operating systems might adopt, we therefore chose to share the modular source codes, Jupyter notebook files, DOME-ML and BioImage Archive (DOI: 10.6019/S-BIAD1852) to respond to the editor's request.<br><br>Reviewer #1<br>The authors present a pipeline for the identification of growth stages in germinating seedlings. YOLO is used to identify seedlings and growth stages, a U-Net is used to segment these seedlings including the emerging root and shoot. The root is tracked over time using a graph approach, allowing temporal dynamics to be measured.<br>The paper does a good job of explaining the pipeline, and has a lot of tables of results showing various traits and experiments that have been conducted. Most of the work takes place on wheat, with smaller studies on other species. The work builds upon a previous work SeedGerm, which has attracted a good amount of interest, and as such I think there is demand for tools such as this. I have some suggestions that I think could improve the manuscript and its impact:<br>Response: Many thanks for your positive comments.<br><br>- The distinction between this work and it's predecessor was not completely clear to me. Seedgerm outlines the platform, but also machine learning derived traits? It would be helpful to be very clear what is new here.<br>Response: Thanks for your comments! We have now justified this question in Lines 374-378, stating that SeedGerm was developed for seed-lot level germination analysis, our study partially inherited its hardware design and seed-lot level source codes for analysis. Instead of traditional ML models, SeedGerm-VIG incorporated DL models into the pipeline to improve the generalisation and accuracy for temporal seed-level analysis and vigour-related feature assessment.<br><br>- The adaptation of the work to new species e.g. barley could be more clear. Did you re-train the network to account for the change in species and background? Or does the network work here without retraining? In which case the generality is impressive, and

something to note in the paper.
Response: Thanks for your question! We used transfer learning to train learning models for rice and barely, with significantly reduced epochs and smaller training sets, suggesting how to apply DL techniques in future multi-species studies (see Line 409-412).

- The use of the phrase "optimised u-net" isn't common. What do you mean by optimised here? That it is trained? Or have you made changes to the architecture to make it more efficient? If not, the "u-net" is sufficient. Or "fine-tuned" if you wish to make clear it has been re-trained.
Response: Thanks for pointing it out! In the Materials and Methods section, we explained that our U-Net was based upon the optimised worked published by Hasal et al. (2023; Ref 40), which is not a standard U-Net (we improved wording in Line 534).

- Line 498, it isn't clear to me why you would train a unet for different numbers of epochs for different classes. Are there multiple unets being trained, or are the classes outputs from the same model? This was not clear.
Response: Thanks for pointing it out! In the Materials and Methods section, we explained that our trained three U-Net models (Line 536) for seed coat, seed/roots, and seedlings

- In its current form the pipeline is not hugely user friendly. The source.zip doesn't contain any code, this is stored in a different file. The code itself isn't on the github code repository at all, only in a zip file within the releases. The instructions are fine, but require a user with python and conda expertise. Once installed, the tool is primarily jupyter notebooks with many relative path strings which will require re-coding should the data change. There is no user interface except that provided by the notebook itself. For a programmer or machine learning expert, none of this will be a problem, but for those who are actually studying seed germination, this may not be practical. It is not obvious which code file to run first, and how to string the different bits of the pipeline together. Arguably there should be a single python file (either UI or command line interface) that exposes all of this functionality in a way where it is easy to run.
Response: We agree with the reviewer's comments. Still, given the lesson learned from SeedGerm, to package the Jupyter notebook with different runtime environments and libraries could easily cause problems to run the packaged sofware, if the libraries and end users' operating systems are upgraded. We therefore chose to share the modular source codes, Jupyter notebook files, DOME-ML, and BioImage Archive (for testing datasets), so that the users could choose what to utilise in their research.
To justify the reviewer's comments, we have created a GUI using widget enabled by Jupyter notebook to facilitate non-expert accessing the SeedGerm-VIG (Lines 620-622 and Lines 238-241; Fig. S14). Screenshots of the GUI can be seen as follows.

Page 71: "The sum of" doesn't make sense to me here. Do you mean "combination of"?
Response: Modified as requested (Line 72).

Reviewer #2
1. Summary
This manuscript introduces SeedGerm-VIG, an automated, open-source pipeline combining YOLOv8x and U-Net for assessing seed vigour using time-lapse phenotypic analysis. The pipeline is tested across wheat, barley, and rice, measuring both static and dynamic traits. A vigour scoring matrix is developed by integrating germination speed and uniformity across three phenological phases. The methodology is novel, and the application is timely and relevant to the plant phenotyping community.

2. Strengths
The integration of multiple deep learning models into a cohesive phenotyping pipeline is technically sound and innovative.
The manuscript presents strong validation using manual scoring, traditional software (SeedExtractor, SeedGerm), and heritability estimates.
The analysis includes a large dataset (29,500+ seed-level images), tested across 21 commercial genotypes.
The open-source release of code and data enhances reproducibility and community

adoption.
Response: Many thanks for your positive comments! We really appreciate your supports!

3. Suggestions for Improvement
A. Deep Learning Model Choices:
While I agree that YOLO and U-Net are SOTA for the proposed technique, it is important to justify their use. Also, why was YOLOv8x specifically chosen when there are much more powerful models out there?
Response: Thanks for your comments! We have now made comparisons of different DL models' performance for seed detection (Table 2) and germination phases identification (Table 3). A line of discussion (Line 349) has also been included.

A. Statistical Analysis
The derivation of the uniformity score (W/L ratio of peak height to data coverage) lacks benchmarking. Suggest including comparisons with standard dispersion metrics such as IQR or coefficient of variation (CV).
Response: Thanks for your suggestion! We have added Supplementary Table S10 and S11and added Line 393 to justify this.

Confidence intervals (e.g., 75%) are shown in radicle growth plots (Fig. 3c) but the statistical basis for these is not explained. Please clarify if bootstrapping or parametric methods were used.
Response: Thanks for your comments! We used seaborn library and these were default parameters. We have clarified in Line 616.

The clustering methods (e.g., affinity propagation, agglomerative clustering) are used without justification. A brief rationale for algorithm choice and cluster count selection is needed.
Response: Thanks for your suggestions! We selected clustering methods that automatically grouped data without requiring a predefined number of clusters, making them suitable for small- to medium-sized datasets (Lines 295-296). To verify the clustering results, we calculated the broad-sense heritability before and after clustering (Tables S2-S4) and found that all of them were above 0.9, indicating that after clustering, genetic effects could explain the vast majority of phenotypic variation in the 21 varieties, highlighting the essential differences between the 21 varieties.

B. Language and Grammar

Line 246: "growth rates did not follow a gradual and smooth increase pattern" could be rephrased for clarity.
Response: Corrected in Line 263.

Throughout: Several instances of overly informal tone (e.g., "nicely differentiate") should be revised to maintain scientific formality. Please conduct a grammar and tone check to ensure that the content is presented in a formal tone fit for a scientific paper.
Response: Thanks for pointing it out! Corrected in Line 396. A native English senior scientist was arranged to go through the manuscript as well.

C. Missing or Weak Analyses

The performance of the root tracking algorithm under conditions of seed/root movement or root intersection is not quantitatively assessed. Include failure case examples or tracking accuracy statistics (e.g., IoU or error rate).
Response: Thanks for your suggestion! When seeds and roots were moving, measures of the second and third roots were not very accurate ($R2 = \sim 0.4$). We improved Fig. S4, Line 362/Lines 438-440, and added Note S2 to provide a complete evaluation.

Limited discussion is provided for how SeedGerm-VIG performs under suboptimal imaging conditions (e.g., occlusion, water reflection). These issues are mentioned in the Limitations (Line 399-416), but not supported with quantitative analysis or visual examples.
Response: Thanks for your suggestion! Note S2 and Lines 438-440 have now been

added.

D. Image Augmentation:
The image augmentation techniques applied should be described to keep the reader more informed (line 160).
Response: Improved in Line 161.

E. Future Study
Although this study computes seed morphometry using 2D images, the downsides of using 2D images has been well established. In the future, I suggest that the authors experiment with 3D imaging techniques. There are many low-cost turntables available in the market that could be coupled with the existing smartphones to capture a panoramic view of the seed for analysis.
Response: Thanks for your suggestions! Lines 452-454 have been added.

Reviewer #3
This manuscript by Dai et al. is a comprehensive and informative work. The process of data collection and analysis are described in good detail, so that on reading through the study most of the questions I thought of had been answered by the end of it. With regards to the requirements of GigaScience for data accessibility and transparency, I believe it fulfils the criteria well. Overall, I think it is an excellent paper thanks to the clear description of the work performed and the accessibility of the data associated with it. I found the figures and tables particularly informative and well-presented, and was grateful for the inclusion of extensive supplementary figures which further illustrated many of the author's points well.
Response: Many thanks for your supports and very positive comments!

I have a few minor comments that I think might improve the paper, but otherwise believe it is of excellent standard.

1.1. Computational reproducibility. Using the code and data that the authors provided, I attempted to reproduce some of the results in the paper. This was a little difficult as the structure of the github repository was not particularly clear. For example, the "release" page contains some of the jupyter notebooks but the main repository does not. Perhaps the jupyter notebooks could be added to the main repository? The same goes for the DL models. I had to add both directories to the repo after cloning it myself.
Response: Thanks for your suggestions! We have modified readme file in the GitHub repo for detailed explanation (https://github.com/The-Zhou-Lab/SeedGerm-VIG/blob/main/README.md).

1.2. After getting the code and models into the repo, the next issue was finding the appropriate version of python to use. I subsequently found that version 3.7 was specified in the methods, but it would be worth also indicating this in the repository.
Response: Thanks for your suggestions! Similarly, we have modified Readme.md to justify this.
1.3. Environments and packages. When installing some of the packages at the versions recorded in the repo, conflict errors arose. For example, between tensorflow 2.2. and scipy 1.7.3. Downgrading scipy fixed this issue, but perhaps the authors could create a virtual environment and a requirements.txt file to list python and package versions which work together.
Response: Thanks! We have modified Readme.md to justify this request.

1.4. Most of the notebooks ran without issues, however there were a few errors. The mask_prediction.ipynb ran but produced images without masks. This may just be an issue reproducing the analysis on a new machine, but I would be grateful if the authors could check that this notebook runs correctly on other computers.
Response: Thanks for trialling our work! We have now improved the .ipynb file and uploaded to the GitHub. Please do use the URL (https://github.com/The-Zhou-Lab/SeedGerm-VIG/releases) to access the latest version of the uploaded notebook file.

1.5. The authors could add a note to tell others to run phase_detection.ipynb before the phase_correction_and_quantification.ipynb if they do not have GPU support for pytorch (as I didn't), to avoid errors about using CPU instead of GPU. Or perhaps just add a

Powered by Editorial Manager® and ProduXion Manager® from Aries Systems Corporation

	note about setting up pytorch with GPU.<br>Response: Thanks for your comments! We have modified Readme.md to justify this request.<br><br>Overall, the notebooks were easy to follow and reproduce. There were some minor differences in results, which could maybe be mitigated by specifying a seed when running the analysis if that is possible. The main improvement would be to document the environment in more detail, and perhaps add greater detail on how to run the notebooks.<br>Response: Thanks for your comments! We have modified Readme.md to justify this request.<br><br>2. The only other significant question I have for the authors is regarding the DL models. These are described in the methods, but I would be interested to know more detail. For example, would it be possible for the authors to share the code used to build these models? I'm aware that the models themselves were included as part of the github release, but I could not easily find information on how exactly they were built. If the authors could share the code and/or other detail regarding how the DL models were built I believe that would be very helpful. The model performance figures in the supplementary are useful to see how well they perform, but if others would like to develop their own DL models or know more about the process I believe it is hard to find that information at the moment.<br>Response: Thanks for your comments! We have listed all of the libraries in the Readme.md.<br><br>3. My last question is only very minor and relates to how SeedGerm-VIG is connected to SeedGerm. Was the original SeedGerm code used in this project? Or was it more the concept of seed germination analysis that is being extended here?<br>Response: Please refer to our answers to the similar question raised by Reviewer #1 (Lines 374-378). SeedGerm was developed for seed-lot level germination analysis, our study partially inherited its hardware design and seed-lot level source codes for analysis. Instead of traditional ML models, SeedGerm-VIG incorporated DL models into the pipeline to improve the generalisation and accuracy for temporal seed-level analysis and vigour-related feature assessment.<br><br>Aside from these minor comments/questions, I think the manuscript was very well presented and that the pipeline could be very useful for breeders with an interest in developing germplasm with improved seed vigour (if they can set it up and use it).<br>Response: A GUI interface has been added in this version of manuscript.
**Additional Information:**	
**Question**	**Response**
Are you submitting this manuscript to a special series or article collection?	No
**Experimental design and statistics**<br><br>Full details of the experimental design and statistical methods used should be given in the Methods section, as detailed in our Minimum Standards Reporting Checklist. Information essential to interpreting the data presented should be made available in the figure legends.<br><br>Have you included all the information requested in your manuscript?	Yes

**Resources**<br><br>A description of all resources used, including antibodies, cell lines, animals and software tools, with enough information to allow them to be uniquely identified, should be included in the Methods section. Authors are strongly encouraged to cite Research Resource Identifiers (RRIDs) for antibodies, model organisms and tools, where possible.<br><br>Have you included the information requested as detailed in our Minimum Standards Reporting Checklist?	Yes
**Availability of data and materials**<br><br>All datasets and code on which the conclusions of the paper rely must be either included in your submission or deposited in publicly available repositories (where available and ethically appropriate), referencing such data using a unique identifier in the references and in the “Availability of Data and Materials” section of your manuscript.<br><br>Have you have met the above requirement as detailed in our Minimum Standards Reporting Checklist?	Yes
GigaScience has policies and guidelines in place for the use of generative AI-writing tools such as ChatGPT. If you have used such writing tools to assist with writing the manuscript this must be declared and cited in the text. Authors should not list AI-writing tools and other AI-assisted technologies as an author or co-author and should acknowledge that they are fully responsible for text generated or refined by AI-writing tools.<p><br><br>A summary of use (particularly in the introduction or among methods) needs to be included at the end of the paper, and the outputs should also be included as a	No

supplementary file hosted in GigaDB or other open repositories. Please read our guidelines for more information. <p><br><br>By submitting to GigaScience, you are aware of the journal's AI-writing tools policy, and if you have declared use of such tools below, you have acknowledged this where appropriate in your manuscript and have made a summary of use and outputs available. <p><br><b>AI-assisted writing tools have been used in the preparation of this manuscript?	

Powered by Editorial Manager® and ProduXion Manager® from Aries Systems Corporation

Manuscript

Click here to access/download;Manuscript;Dai et al. V1.15_clean_20251015_final.docx

SeedGerm-VIG: an open and comprehensive pipeline to quantify seed vigour in wheat and other cereal crops using deep learning powered dynamic phenotypic analysis

Short title: AI-powered seed vigour assessment in cereals

Authors:

Jie Dai[1+], DaiJie@stu.njau.edu.cn, orcid: 0000-0002-3941-576X

Zhenjie Wen[1+], WenZhenjie@njau.edu.cn, orcid: 0000-0002-8191-1070

Mujahid Ali[1+], Mujahid.Ali@stu.njau.edu.cn, orcid: 0000-0001-9239-5705

Jinlong Huang[1], huangjl@stu.njau.edu.cn, orcid: 0009-0003-7332-9915

Shuchen Liu[1], Liushuchen@stu.njau.edu.cn, orcid: 0009-0003-3218-4473

Jianhua Zhao[1], 13770829760@163.com, orcid: 0009-0003-3268-6350

Felipe Pinheiro[2], Felipe.Pinheiro@niab.com, orcid: 0009-0007-4208-3018

Changcai Yang[3], changcaiyang@gmail.com, orcid: 0000-0003-0996-9718

Bin Wang[1], 2023801262@stu.njau.edu.cn, orcid: 0009-0002-2104-0477

Lingzhen Ye[4], yelingzhen@zju.edu.cn, orcid: 0000-0001-6509-9142

Xueying Guan[4*], xueyingguan@zju.edu.cn, orcid: 0000-0002-6528-2518

Ji Zhou[1,2*,+], Ji.Zhou@njau.edu.cn or Ji.Zhou@NIAB.com, orcid: 0000-0002-5752-5524

[+] Contributed equally, [*] Corresponding authors

[1]College of Engineering, College of Agriculture, Academy for Advanced Interdisciplinary Studies, Plant Phenomics Research Centre, Nanjing Agricultural University, Nanjing 210095, China

[2]Data Sciences Department, National Institute of Agricultural Botany (NIAB), Crop Science Centre (CSC), Cambridge CB3 0LE, United Kingdom

[3]Center for Agroforestry Mega Data Science, School of Future Technology, College of Computer and Information Sciences, Fujian Agriculture and Forestry University, Fuzhou 350002, China

[4]Zhejiang Provincial Key Laboratory of Crop Genetic Resources, Institute of Crop Science, Plant Precision Breeding Academy, College of Agriculture and Biotechnology, Zhejiang University, Hangzhou 310058 China

[+]Present address: State Key Laboratory of Plant Trait Design, CAS Center for Excellence in Molecular Plant Sciences, Shanghai Institute of Plant Physiology and Ecology, Chinese Academy of Sciences (CAS), Shanghai 200032, China

Corresponding authors

Ji.Zhou@NJAU.edu.cn or Ji.Zhou@NIAB.com; XueyingGuan@zju.edu.cn

Abstract

As one of the most important cereal crops, wheat (*Triticum aestivum* L.) production and grain quality are essential to many nations in the world. Early developmental phases such as seed germination and seedling establishment are key to wheat's growth and development as they impact directly on crop's early performance and yield potential. Hence, it is critical to develop varieties with favourable early growth characteristics under various growing conditions. Here, we present SeedGerm-VIG, an automated and comprehensive pipeline developed for assessing seed vigour in wheat and other cereal crops. Building on the SeedGerm system, we integrated multiple deep learning models (i.e. YOLOv8x-

Germ and optimised U-Net) and computer vision algorithms into the automated seed-level analysis pipeline to identify key germination phases and measure seed-, root-, and seedling-level phenotypic traits. Then, by using time series directed graph, not only did we track root tips to measure root emergence during the germination procedure (seed-lot R^2 = 84.1%), but we also established a new approach to examine speed and uniformity of germination. These resulted in the establishment of a vigour scoring matrix, through which 21 commercial genotypes' (i.e. 494 randomly sampled seeds, with over 29,500 seed-level images) vigour scores were summarised and evaluated at key phases such as protrusion, radicle emergence, and chloroplast biogenesis, which largely matched with manual assessment based on the International Seed Testing Association (ISTA) guidelines. Finally, we also demonstrated that the SeedGerm-VIG pipeline could be used to assess seed vigour for other cereal crops such as rice (n = 120 seeds) and barley (n = 240 seeds), reliably. In conclusion, we believe that our work demonstrates a valuable step forward to enable the broader plant and crop research community to examine seed vigour and vigour-related features in an automated manner, facilitating effective and reproducible plant selection and relevant seed science research for crop improvement.

Keywords:

Seed vigour, germination, vision-based deep learning, dynamic trait analysis, wheat

Introduction

The imminent challenges of climate changes, growing population, and fertiliser shortage have brought diverse threats to global food security [1]. As one of the most consumed cereal grains in the world, wheat (*Triticum aestivum* L.) production and grain quality are vital to many nations in the world [2,3]. Early developmental phases such as seed germination (growth stage, GS 00-09) and seedling establishment (GS 10-19) are particularly critical for wheat growth and development as low-quality establishment often translates into: (1) a reduced plant density and thus lower yield production, (2) decreased crop effectiveness when competing against weeds, and (3) the potential development of early-stage plant diseases [4–6]. Hence, better seed performance at phases such as protrusion, radicle emergence, seedling establishment is likely leading to improved crop health and performance, ensuring yield potential under field conditions [7,8].

In general, the combination of favourite germination and seedling establishment characteristics under varied growing conditions is defined as high seed vigour by the International Seed Testing Association (ISTA) [9]. As a complex trait, seed vigour is not only an critical aspect of seed quality [10,11], but also key for breeders and researchers to genetically improve plants [7,12]. Due to the importance of high-vigour features such as seed production capability and seed longevity under various storage conditions, seed vigour is widely accepted as an important subject in seed science research and crop improvement, forming the foundation of modern crop breeding, cultivation, agronomic management, and crop production [7,12,13].

Traditionally, assessing seed vigour involves many tests and experiments based on physiological and biochemical parameters of seed lots. For example, one popular method is to evaluate cumulative germination rates to identify high or low vigour groups of seed lots [14]. For such experiments, radicle

length at specific timepoints (e.g. 48 hours after imbibition) were measured, through which the effectiveness of germination and seed viability was estimated [15]. Other methods such as accelerated aging [16], electrical conductivity [17], and seedling emergence [18] were also used, leading to the classification of different vigour groups. Recently, biochemical markers were employed to study vigour, including: (1) sugar content (e.g. glucose and fructose) as sugars can negatively impact on germination [19]; and (2) protein content because it correlates with better seed performance [20]. Spectrometric reflectance and gas chromatography were also introduced to the research domain: (1) spectrometric reflectance collected by multi- and hyper-spectral sensors and spectroscopies was applied to classify high- and low-performing seed lots [21,22]; (2) gas chromatography was employed to estimate seed vigour groups based on volatile organic compounds released during seed metabolism [23].

Among the above approaches, the use of morphological attributes of seeds (e.g. size and shape), radicles (e.g. length), and seedlings (e.g. establishment timing and rates) provides direct evidence in determining seed vigour. For instance, larger seeds were reported to have a higher vigour potential due to greater nutrient reserves [24]; the physical structure of seed coat can influence water uptake during imbibition and thus affect the speed of seed germination [25]. Additionally, phenotypic variations of germination- and vigour-related features can lead to genetic studies of seed vigour, enabling assessment of seed quality and thus crop performance to accelerate crop breeding [26,27].

Since the 21st century, advances in remote sensing, computer vision (CV), and deep learning (DL) technologies open a new door for evaluating germination- and vigour-related traits or aspects [11]. Using colour, spectral and morphological traits, diverse vision-based solutions were introduced, including: (1) *PhenoSeeder* [28] and *SeedExtractor* [29] developed to analyse size- and colour-based traits based on red-green-blue (RGB) seed imagery; (2) *Germinator* [30] and *SeedQuant* [31] built to

identify germination status using colour and contrast features; (3) *SeedGerm* [32] applied supervised machine learning (ML) to quantify cumulative germination rates for plant species such as tomato, pepper, and Brassica; (4) RootNav 2.0 [33] used DL techniques to measure root features for wheat, *Arabidopsis*, and Brassica; (5) hyperspectral imaging and DL models were combined to estimate oil composition [34] and chemical components (e.g. amino acids and lipids) to study seed quality [35].

Still, many of the above methods focus on measuring traits at a specific timepoint (e.g. protrusion or when radicles reach a certain length), which missed the dynamic nature of the early developmental phase as seed performance can fluctuate during germination, particularly when seeds are interacting with external stimuli [36]. After imbibition, radicles play a vital role in absorbing resources and supporting seedling establishment, enabling the active transition phase between heterotroph and photoautotroph [37,38]. Hence, the ability to dynamically measure germination-related phenotypic changes at the seed level (e.g. changes of radicle length) will facilitate plant researchers and breeders to quantitatively examine seed performance, so that seed vigour can be evaluated, objectively and comprehensively.

Here, we present SeedGerm-VIG, an open analytic pipeline developed to assess seed vigour for wheat and other cereals such as rice and barley. Building on the SeedGerm platform previously reported [32], we performed time-lapse imaging of wheat seed germination (for 4-7 days, depending on treatments), followed by the application of SeedGerm-VIG to: (1) identify seed-level germination phases using the YOLOv8x-powered [39] DL model; (2) detect seeds and seedling (e.g. coleoptile) based on an optimised U-Net model [40], through which seed-level morphological features such as seed area, perimeter, length, width and roundness, seed coat colour, and the seedling system could be measured; and (3) track positions of radicles, lateral roots, and seedling tips over time to pinpoint their

emergence rates. Finally, utilising timepoints and growth rates of protrusion, radicle emergence, and chloroplast biogenesis (when seedling turned green), we successfully established a vigour scoring matrix to quantify this complex trait for 21 commercial genotypes at key developmental stages during germination, whose results were partially compatible with the ISTA's guidelines [15].

Findings

Time-lapse seed germination imaging

We first established many low-cost SeedGerm devices [32] to perform RGB overhead time-lapse imaging of seed germination with high-definition (HD) seed-level images (**Fig. 1a**). Experiments were conducted in translucent plastic boxes, with either second-hand smartphones (with a maximum of 3,840 × 2,464 pixels per image) or *Raspberry Pi* image sensors (with a maximum of 4,608 × 4,608 pixels per image) mounted on the top (**Fig. 1b**). A series of RGB images were acquired with one-hour intervals between each shot, recording from dry seeds and imbibition to seedling establishment after the chloroplast biogenesis phase (**Figs. 1c&d**). In total, 21 commercial wheat genotypes known for different germination paces were selected.

To improve the generalisation of the SeedGerm-VIG pipeline, we trialled different experimental settings such as different seeds (e.g. 18, 20, 25, or 30 seeds) and blue or black germination papers (**Fig. 1c**). Depending on plant species, treatments (e.g. cold stratification and ambient temperatures), and potential germination speeds, time-lapse imaging was set from 96 hours (i.e. 4 days) to 168 hours (i.e. 7 days), with HD seed-lot image series (i.e. RGB images acquired in the experiments) stored and synchronised via OneDrive (**Table S1**).

A high-quality training dataset for assessing seed vigour

A seed-lot image series normally covers the entire germination procedure until seedling was fully established (**Fig. 1d**, lower). For the 21 experiments performed in this study, a total of 1,890 seed-lot images were collected. To enable DL model to identify germination phases for every seed in a given experiment, plant specialists first used LabelMe [41] to annotate germination phases based on seed-level images (**Fig. 1d**, upper), including 250 images at imbibition (IMB), 218 at protrusion (PRO), 278 during radicle emergence (RE), and 215 during seedling establishment (SE). These images were randomly selected from 48 seed-lot images with either black (19) or blue germination papers (29).

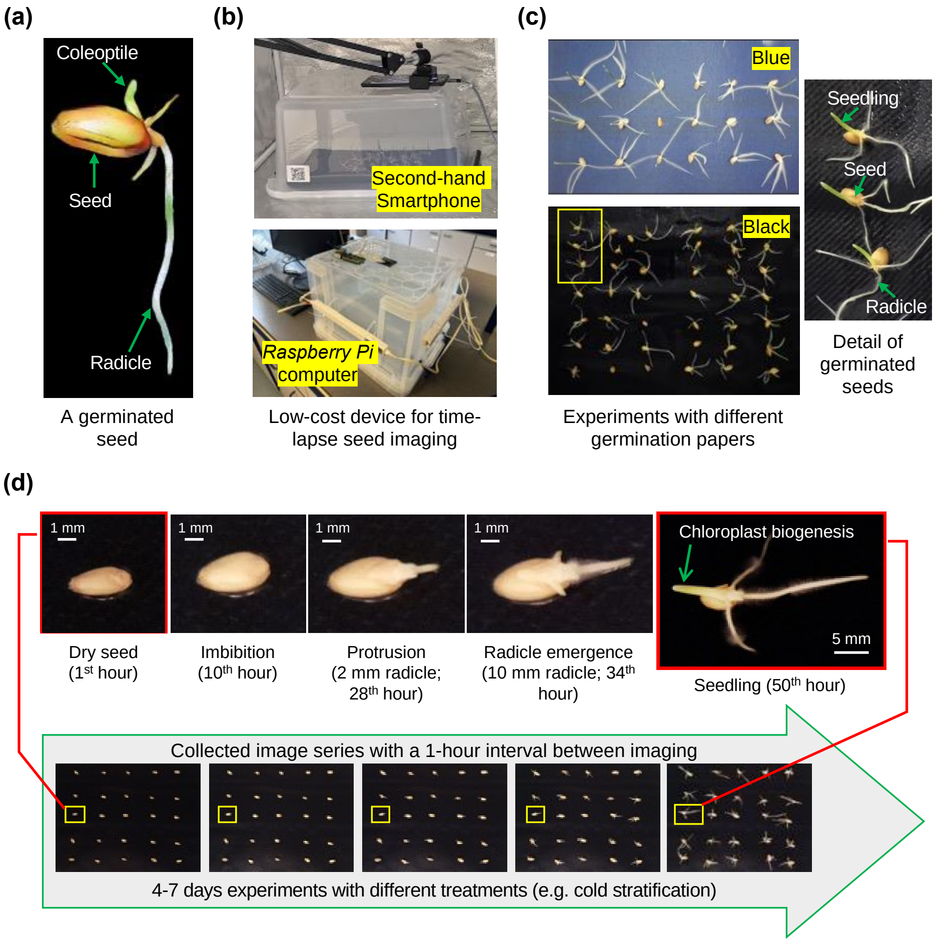

Figure 1. Time-lapse seed germination imaging and acquired image series using SeedGerm devices. (Note: detailed legends are provided in the Figures section).

After image augmentation (e.g. adding noise and image flipping; see **Methods**), an augmented training set called "SeedVig-phase" was established, which contained 3,053 images, relatively evenly distributed across four key germination phases (i.e. 776 images at IMB, 720 at PRO, 784 during RE, and 773 during SE). Additionally, to perform background removal for accurate seed-level trait analysis, more images were annotated, including 644 seeds without roots and seedlings, 702 seeds with roots and seedlings, and 680 seedlings. Similarly, image augmentation was applied and created a training set called "SeedVig-traits", consisting of 1,932 seeds, 2,106 seeds with roots and seedlings, and 2,040 seedlings. The two evenly distributed training sets (**Fig. S1**) were both used in developing and benchmarking DL models.

The YOLOv8x-Germ model for identifying key germination phases

To automate the identification of key germination phases at the seed level in seed-lot image series, we developed a YOLOv8x-powered model called "YOLOv8x-Germ" using the "SeedVig-phase" training set, which was then embedded in the SeedGerm-VIG pipeline (**Fig. 2a**, upper). Seed-level bounding boxes were generated with colour coding to indicate different germination phases (i.e. red, blue, green and orange for IMB, PRO, RE and SE phases, respectively), with confidence levels (0-1, with two decimal places retained) attached to signify the probability of the DL-based estimation (**Fig. 2a**, lower). The YOLOv8x-Germ model was used to identify seed-level germination phases and regions of interest (ROIs) in any seed-lot images collected under dissimilar conditions (**Fig. 2b**). According to the ISTA guideline [9], a specific germination phase is determined when at least 75% of seeds in a given seed lot reach the phase. We therefore used the unseen data in the "SeedVig-phase" dataset to evaluate the DL model's performance when it was used to identify key germination phases for all the seeds in a seed lot.

This resulted in highly accurate identification of germination phases at the seed-lot level, from IMB (100.0%) and SE (100.0%) to PRO (90.0%) and RE (83.3%) phases (**Fig. S2**, right). To standardise DL-based phase identification for experiments with different numbers of seeds, 15 seeds from all the experiments (i.e. seed lots) were sampled (**Fig. S3**).

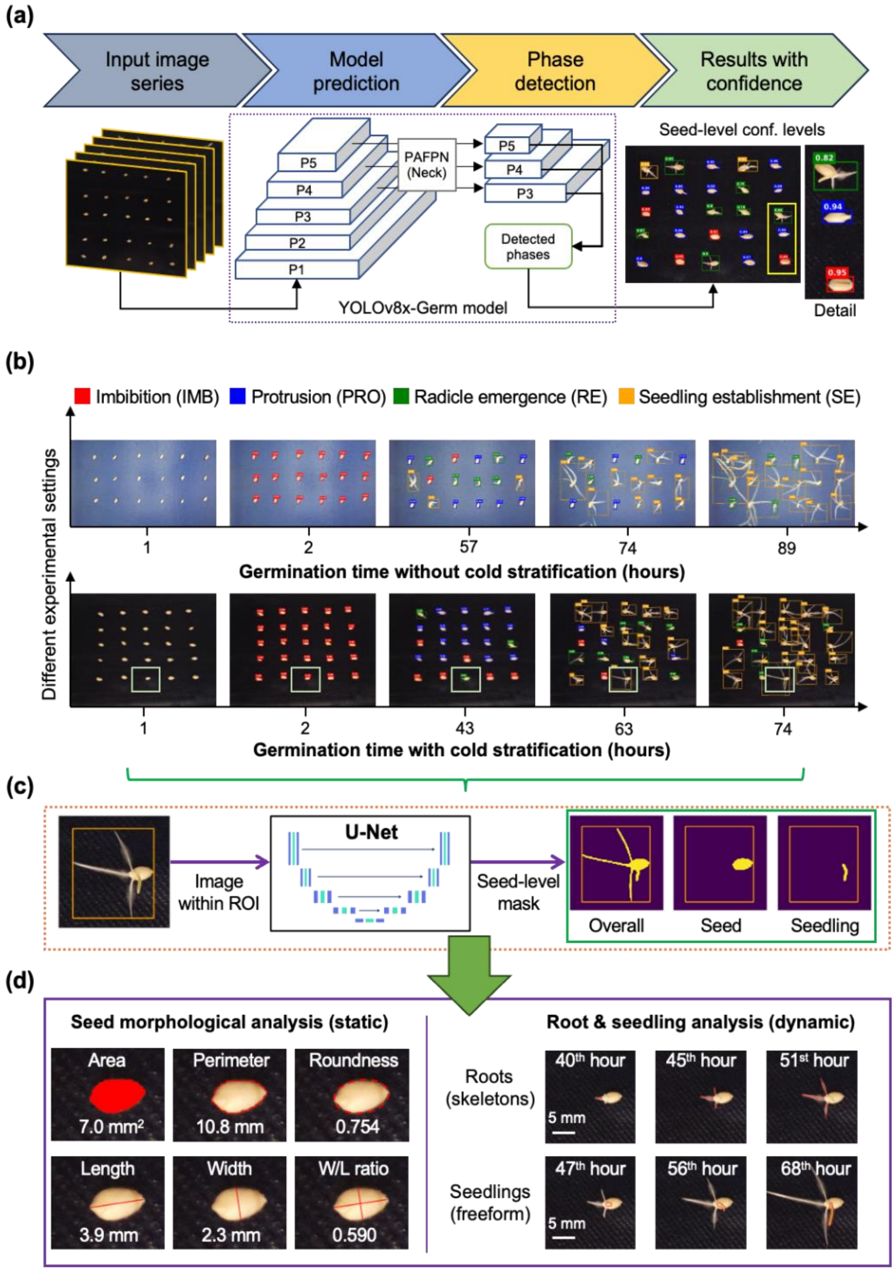

Figure 2. The analysis workflow of SeedGerm-VIG for identifying key germination phases for every seed in a seed lot, followed by the measurement of seed-level static and dynamic germination traits.

U-Net powered seed-level trait analysis and evaluation

To analyse germination- and vigour-related traits, after identifying seed-level bounding boxes (i.e. ROIs) of all the seeds in a seed lot, we developed a U-Net based model to precisely remove background objects (i.e. black and blue germination papers, and root hairs) within the ROIs (**Fig. 2c**). Using a seed in Genotype 7 (G7; Row 5, Column 3; highlighted in **Fig. 2b**), the U-Net model enabled us to retain seed, radicle, lateral roots, and seedling within the ROIs. Then, seed colour and morphological traits such as seed coat colour, area, perimeter, roundness, length, width, width and length (W/L) ratio were quantified to examine dry and imbibed seeds (**Fig. 2d**, left), followed by the measurement of root- (both radicles and two lateral roots) and seedling-related features such as root length (using two dimensional, 2D, skeletons, seedling size and colour; **Fig. 2d**, right). **Table 1** summaries all the static and dynamic traits quantified by the SeedGerm-VIG pipeline at three germination phases.

Table 1. The summary of traits quantified at three germination phases.

Germination phases	Static traits	Dynamic traits
**Imbibition (IMB) & protrusion (PRO)**	Seed area (mm2)	Change rates of seed area (%)
	Seed length (mm)	Change rates of seed length (%)
	Seed width (mm)	Change rates of seed width (%)
	Seed perimeter (mm)	Change rates of seed perimeter (%)
	Seed W/L ratio (0-1)	
	Seed roundness (0-1)	
	Seed coat colour Red (0-255)	
	Seed coat colour Green (0-255)	
	Seed coat colour Blue (0-255)	
	Timepoint of PRO (seed level; hour)	
	Timepoint of PRO (seed lot level; hour)	
**Radicle emergence (RE)**	Radicle length (mm)	Change rates of radicle length (mm/h)
	Root 2 length (mm)	Change rates of root 2 & 3 length (mm/h)
	Root 3 length (mm)	
	Timepoint of RE (seed level; hour)	
	Timepoint of RE (seed lot level; hour)	

**Seedling establishment (SE)**	Seedling length (mm)<br>Timepoint of coleoptile emergence (hour)<br>Timepoint of Chloroplast biogenesis (hour)<br>Timepoint of SE (seed level; hour)<br>Timepoint of SE (seed lot level; hour)	Duration from coleoptile emergence to chloroplast biogenesis (seed level; hour)

Computationally derived traits were evaluated against manual scoring using correlation analyses, resulting in significant correlations for these traits ($P < 0.001$; **Fig. S4**), including seed length (R^2 = 0.894), seed width (R^2 = 0.859), seed perimeter (R^2 = 0.881), seed area (R^2 = 0.881), radicle length (R^2 = 0.800), and seedling size (R^2 = 0.791), which indicated the reliability of the SeedGerm-VIG pipeline in phenotypic analysis. Moreover, we compared traditional germination traits scored manually and the SeedGerm-VIG-derived trait analysis, including germination potential (GP) on the first, second, and third day (i.e. Day 1, 2 and 3) of the experiment (i.e., GP_1, GP_2, GP_3), germination index (GI_1, GI_2, GI_3), mean germination time (MGT), and time to 50% germination (T_{50}). The correlation analyses between the 8 traditional and SeedGerm-VIG-derived traits range from 0.728 to 0.910 (**Fig. S5**), demonstrating the reliability of the SeedGerm-VIG pipeline. Additionally, we computed the broad-sense heritability for the SeedGerm-VIG-derived traits, estimating the genetic influence on them (**Tables S2-S4**).

A graph-based tracking method to study root emergence

To quantify timing, duration, and growth rate of root emergence (including radicle and lateral roots) for a seed lot during the germination procedure, we utilised 2D radicle skeleton (**Fig. 2d**, upper right) to derive dynamic traits (e.g. germination speed and phase-based uniformity) for assessing seed vigour. A temporal directed graph was established to track radicle and lateral root tips for all the seeds in a given experiment. Using a given seed in G7 (Row 1, Column 2; **Fig. 3a**), the graph recorded 2D coordinates

and growth direction of every primary root (i.e. radicle) and lateral root tips emerged from the seed before root intersection, including: (1) radicle and lateral root tips' coordinates and positional changes within 5-hour periods, (2) the distance and growth direction of the same root tip (red-coloured skeletons and blue-coloured growth distances, in mm; **Fig. 3a**), and (3) cumulative root growth (yellow-coloured skeletons, in mm; **Fig. 3a**). Detailed algorithmic explanation is given (**Methods**; **Figure S6**).

When root intersection happened (lower, **Fig. 3a**), the root skeletons were extracted within root intersection regions (highlighted with a light-green-coloured dotted rectangle; **Fig. 3b**). Intersected points, angles and starting points of root skeletons, and the most likely growth direction (i.e. based on the previously recorded root graphs) were determined (**Fig. S7**, left and middle), enabled us to associate root skeletons with their corresponding seeds within intersection regions. The above algorithmic steps were applied to track root tips throughout the germination procedure (**Fig. S7**, right). For example, by tracking roots of 25 seeds (i.e. G7), we visualised the tracking result at the 80th hour, where root skeletons were pseudo-coloured according to time (i.e. 0-100 hours, from dark blue to dark red), followed by the measurement of seed-level root emergence based on assembled root skeletons. Noticeably, root skeletons did not exactly match with the 80th hour image because they were assembled during time when roots were slightly moved during the experiment (**Fig. 3b**). To ensure that the SeedGerm-VIG work could reach the broader plant research community, we created a graphical user interface (GUI) to include the above algorithmic steps using widget, so that nonexperts can use the pipeline relatively easily (see **Methods**).

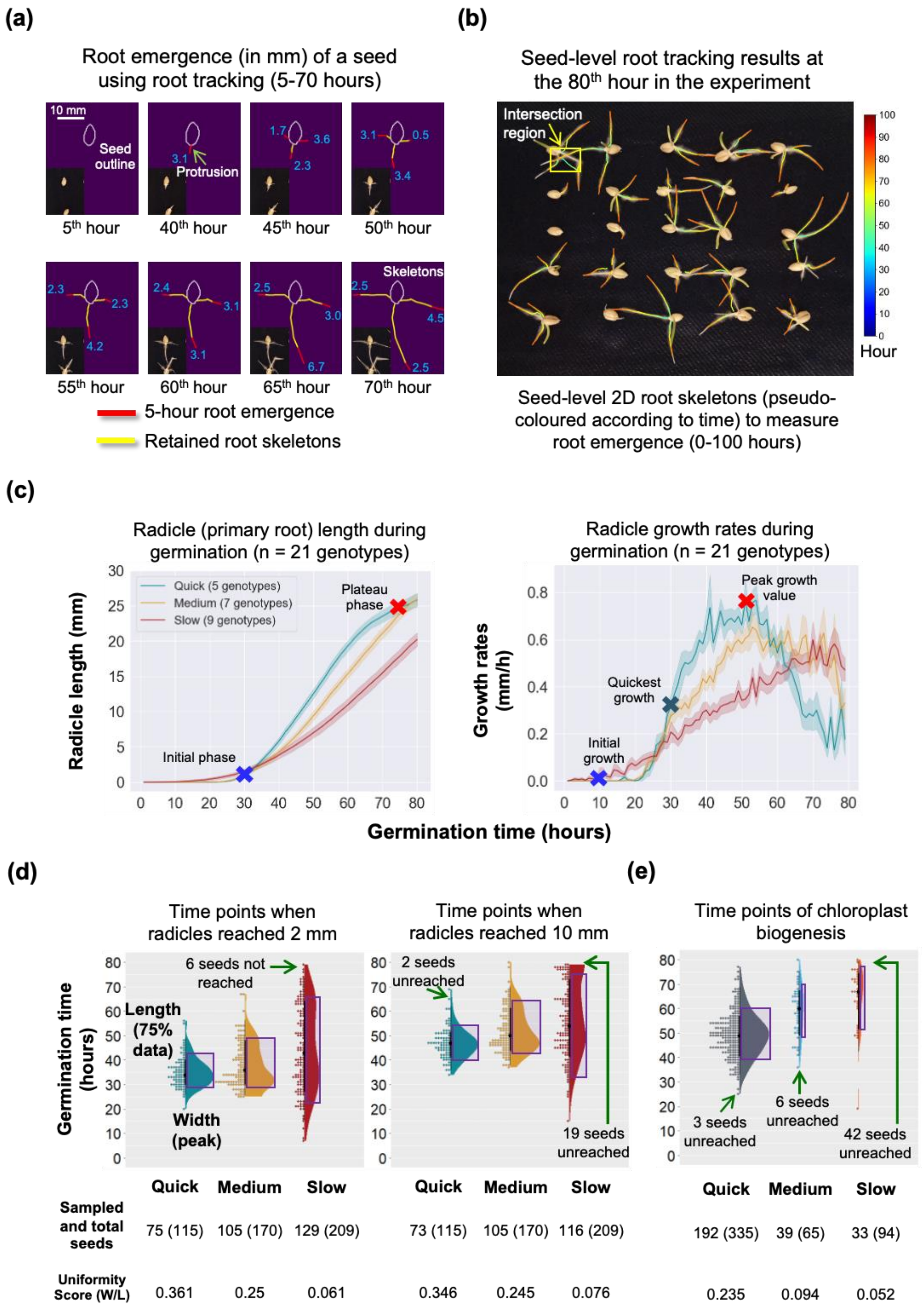

Figure 3. Tracking radicle and lateral root tips, growth speed profiles, and uniformity analysis during key germination phases.

The growth patterns of radicles

Using the root tip tracking algorithm, we examined the 21 genotypes and their radicle emergence characteristics. According to the ISTA guideline [15], when the length of a radicle reaches 2 mm, the seed is "germinated". When a seed's radicle length reaches 10 mm, the seed's root system is "established" [42,43]. In our case, by quantifying radicle length at different timepoints with 1-hour intervals when radicles emerged to 10 mm (roughly between 0 and 80 hours), we were able to calculate 1-hour growth rates for all the genotypes, leading to 21 radicle growth profile curves. All the genotypes followed a gradual increase pattern, with varying growth speeds (**Fig. S8**). Using the radicle length as a metric, we applied the agglomerative clustering method [44] to categorise the 21 curves (**Table S5**) into three categories: Quick (5 genotypes; teal coloured), Medium (7 genotypes; orange), and Slow (9 genotypes; dark red) groups (**Fig. 3c**, left). The differences between the three groups occurred at around 30th hour (highlighted with a blue-coloured cross) when seeds were entering protrusion. The Quick group's radicles began to slow down at 75th hour (highlighted with a red-coloured cross). Noticeably, quick root emergence did not necessarily lead to the longest radicle length as the Medium group's radicle length was slightly longer than those in the Quick group.

Moreover, we studied radicle growth rates of the 21 genotypes, demonstrating varied patterns (**Fig. 3c**, right): (1) growth rates did not increase in a gradual pattern, instead they fluctuated throughout the 80-hour period; (2) timings of highest growth rates of the three groups differed, with the Quick group (teal) peaked at around 50th hour (denoted with a red-coloured cross), followed by around 54th and 70th hours for the Medium and Slow groups; (3) all the groups experienced declines after the peak, with a sharp decline observable from the Quick group; (4) the Slow group (dark red) exhibited an early initial growth (denoted with a blue cross), the smallest peak, and a relatively gradual increase trend compared

with the other groups; (5) by finding the first derivative of the growth rate curve, we were able to locate the timepoint when radicle growth was changing at the quickest rate (indicated with a dark-teal cross), suggesting the most active phenotypic changes of radicle length. For the above curves (**Fig. 3c**), shaded regions were provided to represent the confidence interval (75%).

The uniformity scoring of vigour-related features

Building on the measures of growth patterns (i.e. germination speed), we further studied how to derive uniformity of the seed lots during the germination procedure. For the two radicle emergence stages (i.e. 2 mm for germination and 10 mm for root system establishment), we employed raincloud plots (the x-axis shows the three growth groups) to present the distribution of timepoints (y-axis) when seed lots entered germination and root establishment (**Fig. 3d**): (1) the Quick group (teal) had a narrow and concentrated distribution, with most values clustered around 30–40 hours; (2) the Medium group (orange) had a broader and more stretched distribution, with a central tendency around 30–50 hours; and (3) the Slow group's (dark red) data distribution was the widest and most spreading, with values covered across the y-axis and some seeds did not reach target radicle lengths (i.e. 2 and 10 mm, indicated with green-coloured arrows). Because the three groups had dissimilar ranges and centred values when radicle reached the two lengths, we therefore computed the ratio of the peak value (when most of the seeds reached 2 and 10 mm) and the coverage of 75% data (highlighted with purple-shading rectangles in **Fig. 3d**), resulting in a uniformity score (0-1) measuring how similar a given germination speed group elongated during different phases. This enabled us to effectively differentiate the three groups in terms of the RE uniformity, showing genotypes belonging to the Quick group had higher uniformity scores (i.e. 0.361 and 0.346 at the two phases) compared to the genotypes in Medium (around 30% less

than the Quick group) and Slow (over 80% less) groups.

Similarly, using the timepoints when chloroplast biogenesis occurred (seedling turned green; **Table S6**), we applied affinity propagation clustering [45] to classify the genotypes without pre-defining cluster number, followed by the analysis of their data distribution and uniformity (**Fig. 3e**). Three categorises were identified. While the Quick group (gray) exhibited the broadest distribution and the Slow group (light red) the narrowest, genotypes in the Quick group had a higher uniformity score (0.235) compared to those in the Medium (light blue; around 60% less) and Slow (over 70%) groups. Additionally, using the raincloud plots (**Figs. 3d&e**), we identified seeds that did not reach a certain phase. For example, 6 (out of 129) and 19 (out of 116) seeds in the Slow group did not reach 2 mm and 10 mm radicle lengths, whereas the majority of the seeds in the Quick and Medium groups established the root system successfully. As for the chloroplast biogenesis, the Slow group had 42 (44.7%) seeds did not reach this phase during the 80-hour monitoring period compared with only 3 seeds (0.9%) in the Quick group and 6 seeds (9.2%) in the Medium group.

A comprehensive assessment of seed vigour

Traditional seed vigour assessment often missed the dynamic nature of this complex trait as its performance could vary throughout the germination procedure. Hence, to facilitate an efficient and reproducible method to assess seed vigour, we created a comprehensive matrix to incorporate germination speed and uniformity at different germination phases into the assessment of seed vigour (**Fig. 4**). Using the phase-based classification of growth patterns (i.e. speed) and associated uniformity scores computed for the 21 genotypes (**Tables S7 & S8**), we categorised the genotypes into three groups during the PRO, RE and SE phases using the affinity propagation method [45]. Then, we set the Quick

group as 3 points, Medium as 2 points, Slow as 1 point and multiplied them with their uniformity scores (i.e. Speed × Uniformity), which resulted in three overall vigour groups: (1) five Low-vigour genotypes (G1, G2, G3, G18 and G19), (2) 11 Medium-vigour genotypes (G7, G9, G10, G12, G13, G14, G15, G16, G17, G20 and G21), and (3) five High-vigour genotypes (G4, G5, G6, G8, and G11). We compared the above vigour results with traditionally assessed vigour groups (i.e. 5 Low-vigour genotypes and 16 High-vigour genotypes; **Table S9**) and found that the manually scored Low-vigour (n = 5) and High-vigour (n = 5) genotypes matched with the SeedGerm-VIG derived lines, while the Medium-vigour group could not be assessed as manual scoring could not identify genotypes with this feature.

Genotypes	PRO phase (< 2 mm)		RE phase (2-10 mm)		SE phase (chloroplast biogenesis)		Overall vigour
	Speed	Uniformity	Speed	Uniformity	Speed	Uniformity	
G1	Slow	0.03	Slow	0.04	Slow	0.01	0.08
G2	Slow	0.02	Slow	0.01	Slow	0.01	0.04
G3	Medium	0.02	Slow	0.03	Slow	0.01	0.08
G4	Quick	0.31	Medium	0.29	Quick	0.04	1.63
G5	Quick	0.59	Quick	0.21	Quick	0.04	2.53
G6	Quick	0.23	Medium	0.22	Quick	0.10	1.43
G7	Medium	0.10	Medium	0.09	Quick	0.08	0.61
G8	Quick	0.33	Medium	0.30	Quick	0.10	1.90
G9	Quick	0.12	Slow	0.05	Quick	0.01	0.45
G10	Quick	0.14	Quick	0.09	Quick	0.04	0.80
G11	Quick	0.69	Medium	0.46	Quick	0.17	3.51
G12	Quick	0.16	Quick	0.09	Quick	0.02	0.82
G13	Medium	0.13	Medium	0.14	Medium	0.03	0.59
G14	Medium	0.04	Slow	0.04	Quick	0.02	0.19
G15	Quick	0.13	Slow	0.11	Quick	0.09	0.78
G16	Quick	0.02	Slow	0.02	Quick	0.02	0.13
G17	Medium	0.05	Medium	0.04	Medium	0.01	0.19
G18	Slow	0.02	Slow	0.01	Slow	0.01	0.04
G19	Slow	0.02	Slow	0.07	Slow	0.01	0.09
G20	Quick	0.04	Quick	0.02	Quick	0.01	0.19
G21	Quick	0.11	Quick	0.12	Medium	0.01	0.69

Slow speed or low uniformity   Medium speed or medium uniformity   Quick speed or high uniformity

Figure 4. A comprehensive matrix provides an overview of 21 wheat genotypes' germination speed groups (pseudo-coloured) and uniformity scoring (with two decimal points) at three key phases, followed by the computation of overall seed vigour scores based on measures of phase-based speed and uniformity for the 21 genotypes.

Discussion

As a critical and complex agronomic trait in cereals, seed vigour demonstrates plant seeds' ability to germinate, establish seedlings, and sustain early growth under varied growing conditions [7]. As seed vigour is governed by multiple genetic, biochemical, and physiological factors [14], it is difficult to conduct a comprehensive study through traditional approaches. Still, because high-vigour seeds often lead to uniform crop establishment and thus reduced risks of crop performance (e.g. poor establishment and seedling mortality) under field conditions, breeders and researchers are keen to study and incorporate this trait in the breeding programmes to sustain crop production [46]. Moreover, seed vigour is also treated as a vital target for developing climate-resilient crops, providing plants with a competitive edge against external stimuli at early developmental phases [47]. As previous studies on seed vigour often focus on germination-related traits measured at specific timepoints, which missed the dynamic and complex nature of seed vigour [26], we therefore developed the SeedGerm-VIG methodology, an open and automatic analytic pipeline for quantifying seed-, root- and seedling-related traits and their phenotypic changes during key germination phases, through which we could empower the research community with a new toolkit to assess seed vigour in a comprehensive and dynamic approach. We trust that our toolkit has made advances in a number of areas for seed science research.

The SeedGerm-VIG pipeline for assessing vigour-related traits

Due to recent advances in vision-based DL techniques, DL solutions have been widely applied to plant phenotyping related studies [48]. Using the SeedGerm platform for time-lapse imaging, we integrated two trained DL models (i.e. YOLOv8x and U-Net) into the SeedGerm-VIG pipeline to analyse time-series images collected in dissimilar experimental settings (e.g. the number of seeds, seed treatments,

and germination papers), which outperformed several classical and latest DL models (**Tables 2&3**). This enabled us to establish an automated approach to quantify seed-, root-, and seedling-related traits. For example, the YOLOv8x-Germ model was trained to perform the seed-level identification of key germination phases (i.e. IMB, PRO, RE, and SE) for every seed in a seed lot (**Fig. 2b**), which helped us identify seed-level germination progress and facilitate the segmentation of seed-level ROIs for the following trait analysis. With limited training datasets (3,053 seeds), the YOLOv8x-Germ model achieved high-accuracy predictions of the IMB (84.7%), SE (89.5%), PRO (77.0%), and RE (84.1%) phases based on 1,207 seeds (**Figs. S1&S2**), which can be easily improved when more training datasets are made available through our openly accessible pipeline.

Additionally, within the seed-level ROIs, we used an optimised U-Net model to generate masks of seeds with or without roots and seedlings (**Fig. 2c**), leading to the analysis of seed-level morphological features, root (both radicle and lateral roots) length and tips, and seedling size. According to the correlation analyses (**Fig. S4**), most of the computationally derived traits were significantly correlated with the manual scoring, demonstrating the reliability of the pipeline. Notably, by combining trait analysis with graph-based root tip tracking, we further developed dynamic measures of vigour-related traits by quantifying cumulative root length based on tracking the emergence of root on an hourly basis (**Fig. S9**), through which radicle growth profiles were produced to illustrate the 21 genotypes and their germination speed groups (**Fig. S8**). This also led to the curves of radicle growth rates, providing insights into radicle growth patterns during germination, from 0 to 80 hours.

In particular, we employed a range of computational traits to collectively describe seed vigour, including: (1) by using seed size during IMB to assess the speed of imbibition (**Fig. S10**); (2) by quantifying radicle and lateral root emergence (**Fig. S11**) to quantify timepoints and duration of PRO

and RE phases; (3) by monitoring the presence of greenness on seedlings to obtain the timing of chloroplast biogenesis. Equipping with the above measures, we were able to dissect seed vigour, a highly complex and variable trait, into vigour-related sub-traits to study the trait comprehensively. Compared with our previously published *SeedGerm* platform [32] that was developed for seed-lot level germination analysis, our study partially inherited its hardware design and seed-lot level source codes for automated image processing. Still, for assessing temporal seed-level analysis, instead of traditional ML models, the SeedGerm-VIG pipeline incorporated DL models and graph-based computer vision algorithms to improve the generalisation and accuracy, enabling seed-level trait analysis and vigour-related feature assessment in acquired image series.

The assessment of seed vigour using germination speed and uniformity

In the context of crop improvement, seed vigour can help us select genotypes that are more resilient under different environment and aging conditions [7]. How to assess vigour-related features when seeds are interacting with external stimuli can provide us with insights into the selection of varieties with better crop establishment, productivity, and adaptability in diverse agricultural settings [46]. In our study, besides quantifying timing and speed to evaluate seed performance over time, we also explored the calculation of uniformity, which is another favourite high-vigour feature advised by ISTA [49]. We used raincloud plots to exhibit the data distribution when seeds entered the PRO, RE, and SE phases. While the timepoints of seeds entered a key phase can be used to assess the germination speed and hence the cumulative germination rates, the raincloud plots also assisted us to examine the data distribution of how many seeds entered a given phase over time. For example, using 75% of seeds when they entered a germination phase as “length” and the peak value of the data distribution as “width” (**Fig. 3d**, left),

we computed the ratio of the width and the length (i.e. W/L) that was also proven to be reliable using heritability, coefficient of variation, and interquartile range (IQR) (**Tables S2-S4, S10, S11**), deriving a metric (0-1) to represent the uniformity of radicle emergence and chloroplast biogenesis. The metric could effectively distinguish dissimilar uniformity patterns for the 21 wheat genotypes, as well as the three germination speed groups. Finally, we proposed a seed vigour matrix to incorporate germination speed and uniformity at the three phases into the identification of overall seed vigour, resulting in 5 High-vigour, 11 Medium-vigour, and 5 Low-vigour genotypes, which largely correlated with the manual assessment following the ISTA guideline. To our knowledge, we originated this method to advance automated seed vigour assessment, integrating germination speed and uniformity to identify high-performing seed lots quantitatively.

The application of SeedGerm-VIG for other cereal crops

The SeedGerm-VIG pipeline enabled us to perform germination- and vigour-related trait analysis across the germination procedure, facilitating us to examine desired characteristics such as rapid germination, efficient establishment of root and seedling systems, and the smooth transition from root establishment to photosynthetic autotrophy under varied growing conditions (e.g. cold stratification and different temperatures). To study the generalisation of the proposed method, we further applied the SeedGerm-VIG pipeline to test other cereal crops such as barley and rice through transfer learning [50], so that the pre-trained DL models could be quickly fine-tuned with significantly reduced epochs and smaller training sets, indicating the potential use of DL techniques for multi-species studies. For barley, we carried out experiments with dark red germination papers for twelve commercial lines (i.e. barley genotype, BG; 20 seeds per line) for 90-95 hours (**Fig. 5a**, left).

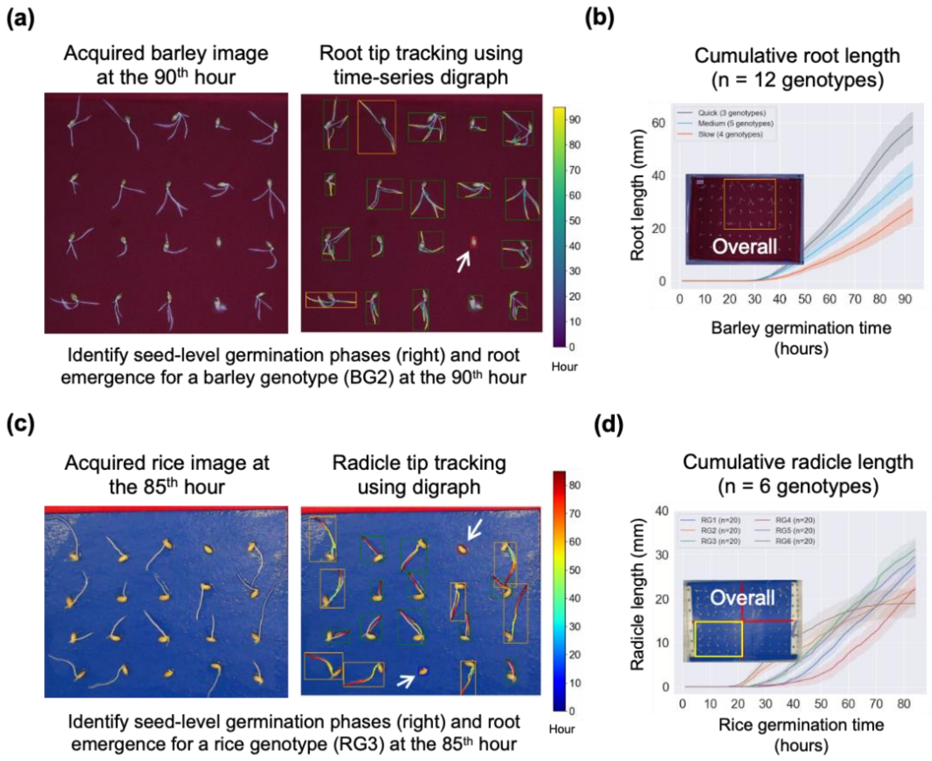

Figure 5. The application of the SeedGerm-VIG pipeline to perform seed-level analyse of root (barley) and radicle (rice) emergence for 12 commercial barley and six rice genotypes.

For example, the YOLO8s-Germ identified germination phases at the 90th hour with coloured bounding boxes, highlighting an ungerminated seed (Column 4, Row 3; enclosed with a red box), which was still at the IMB phase (**Fig. 5a**, right). Then, the graph-based root tracking algorithm was applied to assess seed-level barley roots, generating pseudo-coloured root skeletons that represented root emergence time (**Fig. 5a**, right). Similar to wheat experiments, we computed cumulative root length of the twelve genotypes using 15 sampled seeds, clustering three speed groups for the barley lines (**Fig. 5b**; **Table S12**). For rice, SeedGerm-VIG was applied to process six commercial rice genotypes (RG) trialled on light blue germination papers, identifying seed-level germination status at the 85th hour (with one seed ungerminated and one seed did not reach the PRO phase, both of which were highlighted with coloured bounding boxes), with tracked rice radicels and thus RE speed groups (**Figs. 5b&d**).

Additionally, we performed correlation analyses between computationally derived traits and manually scored radicle length for 12 barley (R^2 >= 0.793; $P < 0.001$) and six rice genotypes (R^2 = 0.815; $P < 0.001$), demonstrating the reliability and generalisability of the SeedGerm-VIG pipeline for other cereal plants (**Figs. S12 & S13**). The uniformity scores also computed, which were closely corresponded to the germination patterns across all the three speed groups for barley when the radicles reached either 2 or 10 mm (**Fig. S12b**), confirming the practical value of the vigour index.

Limitations and future developments

Building upon the ISTA guidelines, this study advanced the assessment of seed germination and vigour from seed lots to the seed level, which empowered us to study seed-level performance during the germination. Still, we have encountered several limits for the SeedGerm-VIG pipeline when suboptimal imaging conditions, root occlusions, and excessive seed or root movements were witnessed (**Note S2**). Some cases could be improved in future studies, including: (1) image quality, which could be affected at early phases due to reflection on germination paper surface as too much water was applied; this issue could be mitigated by controlling water applications at different timepoints; (2) complex root intersection, which could lead to low accuracies of root emergence measures during RE and SE phases; this might be rectified by more labelled training data, cutting-edge DL models, and focusing on radicles with more frequent overhead imaging (e.g. 10-minutes per shot when radicle tip changes are limited); (3) seed and root movement, which complicated seed positions and root tip tracking (see the algorithmic steps in **Note S1**); how to prevent seeds from excessive movement (e.g. 3D printed seed holders) could be considered; (4) the generalisation of the solution, which can be continuously improved with more wheat, barley, and rice genotypes included in the training dataset and thus the refined DL models; (5) the

detailed requirements outlined in ISTA's guidelines (e.g. distinguishing between normal and abnormal seedlings), which will be considered as they can improve the practicality of our pipeline. Additionally, due to the limitation of 2D photography, low-cost devices such as smartphones mounted on turntables can be considered to reconstruct 3D colour point clouds [51], based on which seed germination and vigour can be assessed more comprehensively. Notably, due to the open-source nature of our work, we believe that a community-driven effort could help verify and improve the performance and accuracy of the seed vigour assessment based upon the SeedGerm-VIG pipeline and its easy-to-use GUI.

Besides the above limits, it is valuable to point out that the study presented here is likely opening up new opportunities for us to reveal the genetics of seed vigour, which involve multiple genes and regulatory networks that influence the physiological and biochemical processes. Rapid germination, uniform root and seedling growth, stress tolerance, and even seed longevity are controlled by the interactions of genetic factors and external stimuli. Static and dynamic sub-traits presented in this study can be used to gain an in-depth understanding of the genetic basis of seed vigour, including: (1) genetic components of seed vigour that regulate seed metabolism, seed dormancy and germination, biotic and abiotic stresses, and seed coat and structural features; (2) genetic mapping to identify genetic regions associated with seed vigour using static and dynamic traits, which can identify loci that regulate desired vigour-related features, stress tolerance and seed quality; (3) crop breeding for seed vigour, which enables breeders to incorporate favourable alleles into new crop varieties through marker-assisted selection (MAS) and genomic selection in climate-resilient breeding programmes [52], ensuring better crop establishment, productivity, and resilience to climate change.

Materials and Methods

Plant materials and seed germination experiments

To assess seed vigour in wheat with phenotypic variations, we selected 21 commercial wheat genotypes. These varieties were chosen to capture a broad range of developmental paces during germination (**Fig. 1d**). Seeds were produced in pre-breeding field trials conducted in Cambridgeshire UK (52°23'85"N, 9°60'99"E) and were used within 6 months of harvest. Before the experiments, seed lots were stored at 10°C and 10-15% relative humidity to maintain viability. To evaluate germination speed, some seed lots were treated with cold stratification at 5°C for 24-72 hours in sealed plastic bags (**Table S1**).

Time-lapse imaging with different SeedGerm settings

Germination experiments were carried out in 2022 and 2023 in the UK. Two types of low-cost SeedGerm devices were built for time-lapse imaging: (1) a small translucent plastic germination box (dimensions: 31 × 39 × 48 cm) mounted with fixed second-hand Android smartphones (equipped with Sony IMX278 or IMX319 image sensors; a maximum of 3,840×2,160 pixels per image, roughly 5,000 pixels per seed), and (2) a big germination box (dimensions: 31 × 44 × 71cm) mounted with two types of *Raspberry Pi* cameras (i.e. Sony IMX477 or IMX219, with a maximum of 3,280×2,464 pixels per image; roughly 5,500 pixels per seeds). Different RGB sensors acquired HD images with different resolutions for a flexible hardware design (see image sensors in **Table S1**). Germination experiments were carried out on blue (Bärenstein, Germany) and black (Crown Supplies, UK) seed testing paper under an ambient temperature of 20-22 °C, for 4-7 days depending on the duration of the cold treatments (i.e. 24, 48 and 72 hours). Time-lapse imaging was conducted at regular intervals of one hour, covering from imbibition (**Fig. 1d**, left) to seedling emergence, after chloroplast biogenesis when at least 10 mm

green seedlings were visible (**Fig. 1d**, right).

Data annotation and augmentation

LabelMe [41] was used for data annotation. For the phase detection task of each seed during germination, a total of 48 images were labelled, covering the four germination phases, including 29 images of blue background and 19 images of black background. Due to the resolution differences and growth directions, augmentation methods (e.g. adding noise and flipping) were chosen to mimic imaging-related problems. After augmentation, a total of 151 images (which is called 'SeedVig-phase') were randomly divided into training, testing, and validation sets in a 7:2:1 ratio. For the mask prediction task, we labelled the seeds without roots and seedling, seeds with roots and seedling, and seedlings at early, middle, and late timepoints in the image series, respectively. A total of 32 images for seeds without roots and seedling, 36 for seeds with roots and seedlings, and 38 for seedlings were annotated. Due to the luminance change in different germination experiments, brightness adjustment was applied for image augmentation, and finally 96, 108 and 114 images were generated for seeds without roots and seedling, seeds with roots and seedling, and seedlings, respectively. The images (which is called 'SeedVig-traits') were divided randomly into 70% (for training) and 30% (for testing).

DL model selection and evaluation metrics

To detect seed objects using DL-powered models efficiently and accurately, we trained four DL models using the 'SeedVig-phase' dataset with roots and seedlings, including U-Net [40], PSP-Net [53], HR-Net [54], and DeepLabV3+ [55]. When testing these DL models, every model was trained for 300 epochs and their performance was evaluated through metrics such as mean intersection over union

(mIoU), mean pixel accuracy (mPA), and Accuracy. Through the evaluation, the U-Net model was selected as the architecture to train a DL model for seed objects segmentation (**Table 1**).

Table 2. Performance analysis of different DL models used for seed detection.

Model	mIoU (%)	mPA (%)	Accuracy (%)
U-Net	88.96	92.99	99.10
PSP-Net	82.30	88.00	98.34
HR-Net	83.87	89.60	98.49
DeepLabV3+	80.26	88.07	98.33

Similarly, we trained four DL models using the ‘SeedVig-traits’ dataset to segment seed objects accurately, including SSD [56], Faster-RCNN [57], YOLOv8 [39], and RE-DETR [58]. When testing these DL models, every model was trained for 100 epochs and their performance was evaluated through metrics such as Precision, Recall, mAP50 (mean average precision at IoU =0.5), and F1 score. Through the evaluation, the YOLOv8 model was selected as the architecture for seed detection (**Table 2**).

Table 3. Performance analysis of different DL models trained for identifying germination phases.

Model	Precision (%)	Recall (%)	mAP50 (%)	F1 (%)
YOLOv8	88.10	94.30	95.30	91.11
SSD	82.20	56.75	67.70	61.75
Faster-RCNN	63.38	41.98	52.14	50.25
RT-DETR	72.10	79.30	76.60	75.10

The SeedGerm-VIG pipeline for identifying germination phases

Images of blue germination paper were cropped according to ROIs. For images of black germination paper, rotation or translation were applied to correct slight camera shifts at the start of the image series. After establishing the training set, the standard YOLOv8x model [39] was trained for 2,000 epochs, which was used to identify seed-level germination phases in the experiment. A seed lots' germination phase was defined when 75% seeds of the seed lot entered a specific phase (**Fig. 2b**).

The SeedGerm-VIG pipeline for seed-level trait analysis

After establishing the training set, we followed the a previously reported approach of [40] and used the pre-trained VGG-16 [59] architecture as the backbone (encoding part) of the standard U-Net model [60]. These three U-Net models were trained for 1,000, 400, and 400 epochs to predict masks of seed coat, seed/roots, and seedling of each seed, respectively. The seed morphologies, including seed area, seed length, seed width, seed perimeter, seed W/L ratio, and seed roundness, were quantified through a previous described method [32], while seed colours were quantified by the mean values of R, G, and B channels in the RGB colour space of the seed objects (**Table S13**). The change rates of morphological features were quantified based on the obtained static phenotypes (i.e. values at different timepoints).

Using a root tip tracking algorithm, root tips' positional changes were recorded as the corresponding root change rates and cumulative root lengths were quantified by reconstructing the lines based on the positional changes of root tips across image series (**Fig. S6**). Seedling emergence was recorded when the seedling mask first overlapped with the mask of seeds without roots and seedling within a ROI (i.e., bounding box). For example, the regions corresponding to seedling mask were converted to excess green (ExG) values [61]; and, the chloroplast biogenesis threshold (greenness value) was computed

based on the mean of ExG values [62]. If the ExG value of any pixel in the seedling mask exceeded the threshold, the timepoint was recorded as chloroplast biogenesis. The duration from coleoptile emergence to chloroplast biogenesis was quantified based on the timepoints.

Graph-based root emergence tracking at the seed level

Based on the ROIs obtained, the predicted masks of seeds without roots and seedling and seeds with roots and seedling were used for root tip tracking. After extracting the skeletons (**Fig. 3a**), the root tips were determined by removing skeletons overlapped with seed coat and seedling. Then the root tips were numbered according to their emergence order, with their positional changes of root tips quantified based on their coordinates. As germination progresses, roots often intersect, complicating tracking efforts. To address this issue, we developed algorithms to segment 3-vertice and 4-vertice cliques [63]. After extracting skeletons (**Fig. S7**), branch point (red) or equivalent branch point (i.e. the midpoint of two branch points; blue) were identified, alongside root tips (green) within the local region. Vectors were then constructed from these (equivalent) branch point to the root tips, so that the intersected skeletons could be segmented based on the vector angle, with preference given to angle closer to 180°. Finally, the 2D skeletons were assembled for later analysis.

Building on root tip coordinates and intersection segmentation algorithm, this study utilized temporal graph [64] to track radicle tips. Although the SmartRoot toolkit [65] was reported to use a 90° scanning range to search for intersection roots from a side perspective, the 60° scanning setting used in this study for identifying intersection roots from overhead images were proved more useful in tracking root tips in complex root emergence images. Using Seed 2 of G7 (Row 1 Column 2) as an exemplar (**Fig. 3a**), each root was treated as a temporal graph where the initial vertex V_0 and edge E_0 were both $\varnothing$. The

primary root (radicle) emerged at the 40th hour (t_e), where $V_{t_e\_s}$ denoted the skeleton point connecting to the seed coat and the root tip was taken as V_t, with the skeleton connecting the two nodes defined as E_t. To continuously track root tips of different root, routes connecting C_t (current root tips) to $V_{t\text{-}1}$ were taken into consideration, along with their angles. For instance, at timepoint *t+1*, iterating the root tips in C_t, when root tip P met two requirements: (1) the route connecting P to V_t had the minimum weight (where skeleton points weighed 0 and others weighed 1); (2) the angle between $\overrightarrow{V_{t-1}\ V_t}$ and $\overrightarrow{V_t\ P}$ was no more than 60°, the G_{t+1} was updated accordingly. Through this process, the radicle growth process could be tracked and quantified (**Fig. 3a**). To avoid tracking errors, after radicle intersections, newly emerged radicles were not tracked. Formulas used to perform the tracking are listed below:

$$G_t = \begin{cases} (V_0, E_0), & \text{if } t < t_e - 1 \\ (V_{t_e\_s}, E_0), & \text{if } t = t_e - 1 \\ (V_t, E_t), & \text{if } t > t_e - 1 \end{cases}$$

$$\begin{cases} V_{t+1} = V_t \cup \{P\} \\ E_{t+1} = E_t \cup L(V_t, P) \end{cases}$$

Where at timepoint *t+1*, *P* should meet $P \in C_{t+1}$, when $\|V_t - P\| = \min_{P_l \in C_{t+1}} \| V_t - P_l\|$ and $cos\left(\overrightarrow{V_{t-1}\ V_t}, \overrightarrow{V_t\ P}\right) \geq 0.5$.

Statistical analysis and manual scoring

After trait analysis, the scales provided in **Table S1** were used to convert the pixel units into millimetres (mm). To verify the performance of the SeedGerm-VIG pipeline, a total of 84 images (four images per genotype), covering the early, middle, and late stages of seed germination experiments, were randomly selected for manual scoring. During the IMB phase, seed length, seed width, seed perimeter, and seed area were measured manually using ImageJ [66]. In the subsequent phases (including PRO, SE, and

RE), the lengths of roots and seedlings were assessed manually. Similarly, a total of 18 images of rice and barley were selected for manual measurement of root lengths. To verify the seed vigour of different wheat genotypes, the seed germination images at the 80th hour were used for assessment. Clustering analysis was performed using the 'Scikit-learn' library [67], while correlation analysis utilized the Pearson correlation coefficient and p-value after removing the outliers.

To our knowledge, we could not find any existing toolkits that could be used to quantify the emergence of radicles and seedlings from overhead imagery over time. Hence, we chose two representative research tools such as *SeedExtractor* [29] and *SeedGerm* [32] to evaluate seed-level morphological and colour traits between the SeedGerm-VIG pipeline and the two software packages. The correlation analysis indicated a strong correlation (correlation coefficient, $r > 0.79$) for the morphological traits (e.g. seed length, width, and area) measured between the SeedGerm-VIG and *SeedGerm*, as well as a significant correlation ($r > 0.91$) for RGB colour features measured by the SeedGerm-VIG and *SeedExtractor* (**Table S13**). The above suggested that the SeedGerm-VIG pipeline was able to provide reliable analyses of seed size and colour features compatible with results produced by methods previously reported.

Software implementation

When training the YOlOv8x-Germ and U-Net models, a Windows 10 workstation (16 GB memory, Nvidia GTX 1660Ti GPU, and Intel Core i7-10700F CPU) was used, along with TensorFlow (V2.2) framework [68] and Python (V3.7) for the model implementation. We applied key open scientific development libraries in this study including the scientific data processing library SciPy [69] and the image processing library Scikit-Image [70]. All figures, except raincloud plots, were plotted using the

Python libraries ‘matplotlib’ and ‘seaborn’ [71,72]. Line plots with confidence intervals were created by a parametric method (i.e. confidence intervals = 75%) using the ‘seaborn.relplot’ function with parameters ‘kind=”line”, errorbar=(“ci”, 75)’. Raincloud plots were generated using the R packages ‘ggplot2’ and ‘ggdist’ [73,74]. To facilitate a broader community to access our work, source code and DL models of the SeedGerm-VIG together with executable Jupyter notebooks [75]. When developing the GUI of the pipeline, widget enabled by Jupyter notebook was used together with testing data to assist nonexpert users to execute the SeedGerm-VIG pipeline (**Fig. S14**; **Open Access**).

Abbreviations

Barley genotype (BG), computer vision (CV), deep learning (DL), excess green (ExG), germination index (GI), germination potential (GP), growth stage (GS), graphic user interface (GUI), high definition (HD), imbibition (IMB), International Seed Testing Association (ISTA), machine learning (ML), marker-assisted selection (MAS), mean germination time (MGT), protrusion (PRO), radicle emergence (RE), red-green-blue (RGB), region of interest (ROI), rice genotype (RG), Root 1 (R1; i.e. radicle), Root 2 (R2), Root 3 (R3), seedling establishment (SE), time of 50% cumulative germination (T_{50}), wheat genotypes (WG), width and length ratio (W/L).

Availability of Source Code and Requirements

Project name: SeedGerm-VIG

Project homepage: https://github.com/The-Zhou-Lab/SeedGerm-VIG/

License: MIT license

SciCrunch RRID: SCR_027483

System requirements:

- Operating system: Windows 10/11
- Programming language: Python 3+
- Package management: see the project homepage
- Hardware requirements: NVIDIA GPU with memory ≥ 4GB and system RAM ≥ 4GB (minimum).

Open Access

Source code, trained learning models, and algorithms of SeedGerm-VIG are distributed under the MIT license (Creative Commons Attribution 4.0 international license), permitting academic use, distribution, and reproduction in any medium. Unless otherwise stated, the Creative Commons Public Domain Dedication waiver applies to the data and results made available here [76]. The SeedVig-phase and SeedVig-traits training sets, seed germination image series (i.e. wheat, barley, and rice), are under the CC0 public waiver and are available to the BioImage Archive accession S-BIAD1852 [77]. Test data supporting the results in the article are available at the laboratory's GitHub repository [78]. Other source code, data and user guides are are available in the *GigaScience* repository, GigaDB [79].

Author contributions

Ji Zhou, Jie Dai and Mujahid Ali wrote the manuscript with inputs from all the authors; Zhenjie Wen, Felipe Pinheiro, and Jie Dai conducted experiments under Changcai Yang, Xueying Guan, Lingzhen Ye and Ji Zhou's supervision; Jie Dai, Shuchen Liu, Jinlong Huang, Zhenjie Wen, and Bing Wang built the datasets for deep learning models under Ji Zhou's supervision; Jinlong Huang and Jie Dai, Zhenjie Wen, and Shuchen Liu measured the manual data and performed statical analysis and result interpretation

under Ji Zhou's supervision; Jie Dai, Jianhua Zhao, and Ji Zhou developed the SeedGerm-VIG pipeline; Mujahid Ali, Zhenjie Wen and Xueying Guan helped revise the manuscript. All authors read and approved the manuscript. Jie Dai, Zhenjie Wen, and Mujahid Ali contributed equally to this work.

Funding

This work and the Zhou lab members at NAU were supported by the National Natural Science Foundation of China (32070400 & U24A20402). Ji Zhou and Felipe Pinheiro were partially supported by the Allan & Gill Gray Foundation' Sustainable Productivity for Crop Improvement (G118688 to the University of Cambridge and NIAB). Ji Zhou was supported by the United Kingdom Research and Innovation's (UKRI) Biotechnology and Biological Sciences Research Council (BBSRC) AI in Bioscience Grant (BB/Y513969/1 to Ji Zhou). The UK-China research activities were supported by the BBSRC's International Partnership Grant (BB/Y514081/1 to NIAB). AI model training was partially conducted on the CropDiversity HPC funded by the BBSRC's ALERT grant (BB/X019683/1 to James Hutton Institute).

Acknowledgements

The authors would like to thank all members of the Zhou laboratory at the Nanjing Agricultural University (NAU) China and Cambridge Crop Research, the National Institute of Agricultural Botany (NIAB) UK for fruitful discussions. In particular, the authors would like to thank Jimmy Zhang at Imperial College for his supports in data collection and low-cost SeedGerm hardware development, Jie Zhou, Gang Sun, and Liyan Shen at the NAU for their help improve deep learning modelling.

Competing interests

The authors declare no competing financial interests.

Tables

Table 1. The summary of traits quantified at three germination phases.

Table 2. Performance analysis of different DL models used for seed detection.

Table 3. Performance analysis of different DL models trained for identifying germination phases.

Figures

Figure 1. Time-lapse seed germination imaging and acquired image series using SeedGerm devices.

(**a**) A germinated wheat seed consisting of coleoptile, seed and radicles. (**b**) A set of low-cost SeedGerm devices equipped with second-hand smartphone or *Raspberry Pi* sensors to perform overhead time-lapse imaging. (**c**) Germination experiments with blue and black germination papers. (**d**) Representative seed- and seed-lot level images of genotype 4 (G4) collected at key germination phases during the germination procedure. Scale bars provided to show the size of seed, radicles, and seedling.

Figure 2. The analysis workflow of SeedGerm-VIG for identifying key germination phases for every seed in a seed lot, followed by the measurement of seed-level static and dynamic germination traits.

(**a**) A general analysis workflow for seed-level germination phase identification using the YOLOv8x-Germ model, with seed-level confidence displayed. (**b**) Seed-level germination phases identified by the YOLOv8x-Germ model from seed-lot level image series (upper: G2; bottom: G7) together with bounding boxes, ranging from imbibition (IMB), protrusion (PRO), radicle emergence (RE), to seedling

establishment (SE). For a given experiment, the germination phase (i.e. IMB, PRO, RE, or SE) was defined when over 75% of the seeds reached a certain phase. (**c**) Seed-level masks generated using a standard U-Net model, dividing foreground objects (e.g. seeds, roots, and seedlings) from background signals (e.g. germination papers). (**d**) Phenotypic analysis of seeds, radicles and seedlings based on the identified foreground objects.

Figure 3. Tracking radicle and lateral root tips, growth speed profiles, and uniformity analysis during key germination phases.

(**a**) Radicle emergence of a seed (genotype 7, G7) measured using the root tip tracking algorithm with 5-hour intervals. (**b**) Seed-level radicle tracking results of G7 at the 80th hour, with root skeletons created and pseudo-coloured according to time (0-100 hours). (**c**) Profile curves of radicle length (left) and radicle growth rate (right) between 0 and 80 hours, which were classified into the Quick, Medium, and Slow Speed groups. (**d**) Raincloud plots used to demonstrate how uniform radicles in the three Speed groups reached 2 and 10 mm over time. (**d**) Raincloud plots show how uniform seeds' reached chloroplast biogenesis in the three Speed groups between 0 and 80 hours. Arrows point out seeds did not reach key germination phases. Uniformity scores were provided based on 75% of the data.

Figure 4. A comprehensive matrix provides an overview of 21 wheat genotypes' germination speed groups (pseudo-coloured) and uniformity scoring (with two decimal points) at three key phases, followed by the computation of overall seed vigour scores based on measures of phase-based speed and uniformity for the 21 genotypes.

Figure 5. The application of the SeedGerm-VIG pipeline to perform seed-level analyse of root (barley) and radicle (rice) emergence for 12 commercial barley and six rice genotypes.

(**a**) Barley seeds germination recorded on dark red germination papers and seed-level root emergence identified by the SeedGerm-VIG pipeline for a barley genotype (BG2) at the 90th hour, with seed-level bounding boxes showing seed germination phases (one seed still at protrusion was pointed with a white arrow) and root skeletons pseudo-coloured according to time (0-90 hours). (**b**) Root emergence curves produced to demonstrate germination speed and profiles for twelve barley commercial lines (15 seeds sampled per line) over 93 hours. (**c**) Rice seeds germination recorded on light blue germination papers and seed-level radicle emergence identified by the SeedGerm-VIG pipeline for a rice genotype (RG3) at the 85th hour, with seed-level bounding boxes showing germination phases (one seed was still at imbibition and one seed was at protrusion) and radicle skeletons pseudo-coloured according to time (0-85 hours). (**d**) Radicel emergence curves produced to show germination speed and profiles for six rice commercial lines (15 seeds sampled per line) over 85 hours.

Supplementary Material

Table S1. Experimental settings for 21 commercial wheat varieties for germination.

Table S2. Descriptive statistics and broad-sense heritability of timepoints when radicles reached 2 mm.

Table S3. Descriptive statistics and broad-sense heritability of timepoints when radicles reached 10 mm.

Table S4. Descriptive statistics and broad-sense heritability of timepoints when chloroplast biogenesis was detected.

Table S5. Three clusters of 21 wheat genotypes using the agglomerative clustering algorithm based on radicle growth rates, from 2 mm to 10 mm.

Table S6. Three clusters of 21 genotypes using the affinity propagation algorithm based on timepoints of chloroplast biogenesis.

Table S7. Three clusters of 21 genotypes using the K-means algorithm based on timepoints of key germination phases at the seed-lot level.

Table S8. Three clusters of 21 genotypes using the affinity propagation algorithm based on timepoints of radicle emergence and seedling emergence at the seed-lot level.

Table S9. Manual assessments of the 21 wheat genotypes for germination speed.

Table S10. Coefficient of variation of the 75% data from the three germination speed groups based on timepoints of key germination phases and chloroplast biogenesis.

Table S11. Interquartile range of the data from the three germination speed groups based on timepoints of key germination phases and chloroplast biogenesis.

Table S12. Three clusters of 12 barley genotypes using the affinity propagation algorithm based on radicle growth rates, from 2 mm to 10 mm.

Table S13. Correlation (*R*) of the three automated analytic approaches when measuring different seed parameters.

Note S1. Algorithmic steps to correct seed positions and root tips tracking.

Note S2. Exceptional cases that the SeedGerm-VIG made mistakes.

Figure S1. A high-quality seed germination training set for deep learning modelling.

Figure S2. Confusion matrixes for evaluating the accuracy of identifying key germination phases.

Figure S3. Profile curves of wheat genotypes reaching a germination phase (15 seeds sampled from every genotype).

Figure S4. Correlation analysis between manual and computational measures of seed, root, and seedling

traits across 21 wheat genotypes.

Figure S5. Correlation analysis between traditional and SeedGerm-VIG derived traits across 21 wheat genotypes.

Figure S6. Positional changes (30-65 hours) of root tips for a given seed (Row 3, Column 1) from the G4 seed lot.

Figure S7. The algorithmic steps to identify roots from images with intersected roots.

Figure S8. Profile curves of radicle growth for 21 wheat genotypes during germination.

Figure S9. Profile curves of first three roots for 21 wheat genotypes.

Figure S10. Raincloud plots of seed area and seed width changes during the imbibition (IMB) phase for three germination speed groups.

Figure S11. Root analysis of the first three roots in the Quick, Medium, and Slow germination speed groups.

Figure S12. Root-based germination analysis for twelve barley genotypes.

Figure S13. Radicle emergence analysis for six rice genotypes.

Figure S14. The graphic user interfaces (GUIs) developed using widget in Jupyter notebooks to automate tasks in the SeedGerm-VIG pipeline.

References

1. Seppelt R, Klotz S, Peiter E, et al. Agriculture and food security under a changing climate: An underestimated challenge. *iScience*. 2022; 25(12):105551. 10.1016/j.isci.2022.105551.

2. Hawkesford MJ, Araus JL, Park R, et al.. Prospects of doubling global wheat yields. *Food Energy Secur*. 2013; 2(1):34-48. 10.1002/fes3.15.

3. Li S, Tian Y, Wu K, et al.. Modulating plant growth–metabolism coordination for sustainable

agriculture. *Nature*. 2018; 560:595-600. 10.1038/s41586-018-0415-5.

4. Gang A, Vyas A VH. Toxic effect of heavy metals on germination and seedling growth of wheat. *J Environ Res Dev*. 2013;8:206–13.

5. Hojat Salehzade, Mousa Izadkhah Shishvan, Mehdi Ghiyasi, et al. Effect of Seed Priming on Germination and Seedling Growth of Wheat (*Triticum aestivum* L.). *Res J Biol Sci*. 2009;4(5):629–31.

6. Mwendwa JM, Brown WB, Weidenhamer JD, et al.. Evaluation of commercial wheat cultivars for canopy architecture, early vigour, weed suppression, and yield. *Agronomy*. 2020; 10(7):983. 10.3390/agronomy10070983.

7. Finch-Savage WE, Bassel GW. Seed vigour and crop establishment: Extending performance beyond adaptation. *J Exp Bot*. 2016; 67(3):567-91. 10.1093/jxb/erv490.

8. S. Ambika, V. Manonmani GS. Review on effect of seed size on seedling vigour and seed yield. *Res J Seed Sci*. 2014;7(2):31–8. 10.3923/rjss.2014.31.38

9. ISTA. Seed Vigour Testing. *Int Rules Seed Test*. In: The International Seed Testing Association (ISTA). Switzerland: Bassersdorf; 2021. p. i-15-20(20).

10. McDonald MB. Seed quality assessment. *Seed Sci Res*. 1998; 8(2):265-76. 10.1017/s0960258500004165.

11. Powell AA. Seed vigour in the 21 st century. *Seed Sci Technol*. 2022; 50:45-73. 10.15258/sst.2022.50.1.s.04.

12. Bettey M, Finch-Savage WE, King GJ, et al. Quantitative genetic analysis of seed vigour and pre-emergence seedling growth traits in Brassica oleracea. *New Phytol*. 2000; 148(2):277-86. 10.1046/j.1469-8137.2000.00760.x.

13. Penfield S, King J. Towards a systems biology approach to understanding seed dormancy and

germination. *Proc R Soc B Biol Sci*. 2009; 276(1673):3561-69. 10.1098/rspb.2009.0592.

14. Marcos-Filho J. Seed vigor testing: An overview of the past, present and future perspective. *Sci Agric*. 2015; 72(4):363-374. 10.1590/0103-9016-2015-0007.

15. Khajeh-Hosseini M, Gallo C, Wagner M H, et al. Proposal for the addition of wheat (*Triticum aestivum*) as a species to which the radicle emergence test for seed vigour can be applied. *Seed Test Int*. 2018;157:40–1.

16. Matera TC, Pereira LC, Braccini AL, et al. Accelerated aging test and its relationship to physiological potential of soybean seeds. *J Seed Sci*. 2019; 41(3):301-8. 10.1590/2317-1545v41n3212746.

17. Matthews S, Powell A. Electrical Conductivity Vigour Test: Physiological Basis and Use. *Seed Test Int*. 2006;131:32–5. 10.15258/sst.2009.37.3.20

18. Demir I, Ermis S, Okçu G, et al. Vigour tests for predicting seedling emergence of aubergine (*Solanum melongena* L.) seed lots. *Seed Sci Technol*. 2005;33(2):484-4. 10.15258/sst.2005.33.2.20.

19. Santos JF, Dirk LMA, Bruce Downie A, et al. Reciprocal effect of parental lines on the physiological potential and seed composition of corn hybrid seeds. *Seed Sci Res*. 2017;27(3):206-16. 10.1017/S0960258517000095.

20. Wen D, Hou H, Meng A, et al . Rapid evaluation of seed vigor by the absolute content of protein in seed within the same crop. *Sci Rep*. 2018;8:5569. 10.1038/s41598-018-23909-y.

21. Liu W, Liu J, Jiang J, et al. Comparison of partial least squares-discriminant analysis, support vector machines and deep neural networks for spectrometric classification of seed vigour in a broad range of tree species. *J Near Infrared Spectrosc*. 2021;29(1):33-41. 10.1177/0967033520963759.

22. Al-Amery M, Geneve RL, Sanches MF, et al. Near-infrared spectroscopy used to predict soybean

seed germination and vigour. *Seed Sci Res*. 2018;28(3):245-52. 10.1017/S0960258518000119.

23. Umarani R, Bhaskaran M, Vanitha C, et al. Fingerprinting of volatile organic compounds for quick assessment of vigour status of seeds. *Seed Sci Res*. 2020;30(2):112-21. 10.1017/S0960258520000252.

24. Snider JL, Collins GD, Whitaker J, et al . The impact of seed size and chemical composition on seedling vigor, yield, and fiber quality of cotton in five production environments. *F Crop Res*. 2016;193:186-95. 10.1016/j.fcr.2016.05.002.

25. Mandizvo T, Odindo AO. Seed coat structural and imbibitional characteristics of dark and light coloured Bambara groundnut (*Vigna subterranea* L.) landraces. *Heliyon*. 2019;5(2):e01249. 10.1016/j.heliyon.2019.e01249.

26. Reed RC, Bradford KJ, Khanday I. Seed germination and vigor: ensuring crop sustainability in a changing climate. *Heredity*. 2022;128:450-9. 10.1038/s41437-022-00497-2.

27. Wani BA, Ram M, Yasin BA, et al. Seedling vigour in wheat (*Triticum aestivum* L.) as a source of genetic variation and study of its correlation with yield and yield components. *African J Agric Res*. 2013;8(4):370-2. 10.5897/ajar12.1375.

28. Jahnke S, Roussel J, Hombach T, et al . *pheno*Seeder - A Robot System for Automated Handling and Phenotyping of Individual Seeds. *Plant Physiol*. 2016;172(3):1358-70. 10.1104/pp.16.01122.

29. Zhu F, Paul P, Hussain W, et al. *SeedExtractor*: An Open-Source GUI for Seed Image Analysis. *Front Plant Sci*. 2021;11:581546. 10.3389/fpls.2020.581546.

30. Joosen RVLL, Kodde J, Willems LAJJ, et al . Germinator: A software package for high-throughput scoring and curve fitting of Arabidopsis seed germination. *Plant J*. 2010;62(1):148-59. 10.1111/j.1365-313X.2009.04116.x.

31. Braguy J, Ramazanova M, Giancola S, et al. SeedQuant: a deep learning-based tool for assessing stimulant and inhibitor activity on root parasitic seeds. 2021;186(3):1632-44. 10.1093/plphys/kiab173.

32. Colmer J, O'Neill CM, Wells R, et al. SeedGerm: a cost-effective phenotyping platform for automated seed imaging and machine-learning based phenotypic analysis of crop seed germination. *New Phytol*. 2020;228(2):778-93. 10.1111/nph.16736.

33. Yasrab R, Atkinson JA, Wells DM, et al. RootNav 2.0: Deep learning for automatic navigation of complex plant root architectures. *Gigascience*. 2019;8(11):giz123. 10.1093/gigascience/giz123.

34. da Silva Medeiros ML, Cruz-Tirado JP, Lima AF, et al. Assessment oil composition and species discrimination of *Brassicas* seeds based on hyperspectral imaging and portable near infrared (NIR) spectroscopy tools and chemometrics. *J Food Compos Anal*. 2022;107:104403. 10.1016/j.jfca.2022.104403.

35. Shi T, Gao Y, Song J, et al. Using VIS-NIR hyperspectral imaging and deep learning for non-destructive high-throughput quantification and visualization of nutrients in wheat grains. *Food Chem*. 2024;461:140651. 10.1016/j.foodchem.2024.140651.

36. Weitbrecht K, Müller K, Leubner-Metzger G. First off the mark: Early seed germination. *J Exp Bot*. 2011;62(10):3289-309. 10.1093/jxb/err030.

37. Pogson BJ, Ganguly D, Albrecht-Borth V. Insights into chloroplast biogenesis and development. *Biochim Biophys Acta - Bioenerg*. 2015;1847(9):1017-24. 10.1016/j.bbabio.2015.02.003.

38. Maqbool S, Hassan MA, Xia X, et al. Root system architecture in cereals: progress, challenges and perspective. *Plant J*. 2022;110(1):23-42. 10.1111/tpj.15669.

39. Reis D, Kupec J, Hong J, et al. Real-Time Flying Object Detection with YOLOv8. *arXiv*. 2023. 10.48550/arXiv.2305.09972.

40. Hasal M, Pecha M, Nowaková J, et al. Retinal Vessel Segmentation by U-Net with VGG-16 Backbone on Patched Images with Smooth Blending. In: Barolli L, editor. *Advances in Intelligent Networking and Collaborative Systems*. Switzerland: Springer; 2023. p. 465–74.

41. Russell BC, Torralba A, Murphy KP, et al . LabelMe: A database and web-based tool for image annotation. *Int J Comput Vis*. 2008;77:157-73. 10.1007/s11263-007-0090-8.

42. Mangrich ME, Saltveit ME. Heat shocks reduce chilling sensitivity of cotton, kenaf, okra, and rice seedling radicles. *J Am Soc Hortic Sci*. 2000;125(3):377-82. 10.21273/jashs.125.3.377.

43. Rahnama A, Fakhri S, Meskarbashee M. Root growth and architecture responses of bread wheat cultivars to salinity stress. *Agron J*. 2019;111(6):2991-8. 10.2134/agronj2018.12.0795.

44. Jain AK, Murty MN, Flynn PJ. Data clustering: A review. *ACM Comput Surv*. 1999;31(3):264-323. 10.1145/331499.331504.

45. Frey BJ, Dueck D. Clustering by passing messages between data points. *Science*. 2007;315(5814):972-6. 10.1126/science.1136800.

46. Li W, He X, Chen Y, et al. A wheat transcription factor positively sets seed vigour by regulating the grain nitrate signal. *New Phytol*. 2020;225(4):1667-80. 10.1111/nph.16234.

47. Ramappa S, Joshi MA, Krishna H, et al. Unravelling the Genetic Basis of Moisture Deficit Stress Tolerance in Wheat for Seedling Vigour-Related Traits and Root Traits Using Genome-Wide Association Study. *Genes*. 2023;14(10):1902. 10.3390/genes14101902.

48. Arya S, Sandhu KS, Singh J, et al. Deep learning: as the new frontier in high-throughput plant phenotyping. *Euphytica*. 2022;218:47. 10.1007/s10681-022-02992-3.

49. Demir I, Ermis S, Mavi K, et al. Mean germination time of pepper seed lots (*Capsicum annuum* L.) predicts size and uniformity of seedlings in germination tests and transplant modules. *Seed Sci*

Technol. 2008;36(1):21-30. 10.15258/sst.2008.36.1.02.

50. Krizhevsky A, Sutskever I, Hinton GE. ImageNet classification with deep convolutional neural networks. *Commun ACM*. 2017;60(6):84-90. 10.1145/3065386.

51. Gibbs JA, Pound M, French AP, et al. Plant phenotyping: An active vision cell for three-dimensional plant shoot reconstruction. *Plant Physiol*. 2018;178(2):524-534. 10.1104/PP.18.00664.

52. Moore C, Rebetzke G. Genomic regions for embryo size and early vigour in multiple wheat (*Triticum aestivum* L.) populations. *Agronomy*. 2015;5(2):152-79. 10.3390/agronomy5020152.

53. Zhao H, Shi J, Qi X, et al. Pyramid Scene Parsing Network . *2017 IEEE Conf Comput Vis Pattern Recognit*. 2017;6230-9. 10.1109/CVPR.2017.660.

54. Wang J, Sun K, Cheng T, et al. Deep High-Resolution Representation Learning for Visual Recognition. *IEEE Trans Pattern Anal Mach Intell*. 2021;43(10):3349-64. 10.1109/TPAMI.2020.2983686.

55. Chen LC, Zhu Y, Papandreou G, et al. Encoder-Decoder with Atrous Separable Convolution for Semantic Image Segmentation. In: Ferrari V, Hebert M, Sminchisescu C, Weiss Y, editors. *Comput Vis -- ECCV 2018*. Cham: Springer; 2018. p. 833–51.

56. Liu W, Anguelov D, Erhan D, et al. SSD: Single Shot MultiBox Detector. In: Leibe B, Matas J, Sebe N, Welling M, editors. *Comput Vis -- ECCV 2016*. Cham: Springer; 2016. p. 21–37.

57. Ren S, He K, Girshick R, et al. Faster R-CNN: Towards Real-Time Object Detection with Region Proposal Networks. *IEEE Trans Pattern Anal Mach Intell*. 2016;39(6):1137-49. 10.1109/TPAMI.2016.2577031.

58. Zhao Y, Lv W, Xu S, et al. DETRs Beat YOLOs on Real-time Object Detection. *2024 IEEE/CVF Conf Comput Vis Pattern Recognit*. 2024;16965-74. 10.1109/CVPR52733.2024.01605.

59. Simonyan K, Zisserman A. Very deep convolutional networks for large-scale image recognition. *3rd Int Conf Learn Represent ICLR 2015 - Conf Track Proc*. 2015;1-14. https://doi.org/10.48550/arXiv.1409.1556.

60. Ronneberger O, Fischer P, Brox T. U-Net: Convolutional Networks for Biomedical Image Segmentation. In: Navab N, Hornegger J, Wells WM, Frangi AF, editors. *Medical Image Computing and Computer-Assisted Intervention – MICCAI 2015*. Cham: Springer; 2015. p. 234–41.

61. Woebbecke DM, Meyer GE, Von Bargen K, et al. Color indices for weed identification under various soil, residue, and lighting conditions. *Trans Am Soc Agric Eng*. 1995;38(1):259-69. 10.13031/2013.27838.

62. Ridler TW, Calvard S. Picture thresholding using an iterative selection method. *IEEE Trans Syst Man Cybern*. 1978;8(8):630-2. 10.1109/TSMC.1978.4310039.

63. De J, Li H, Cheng L. Tracing retinal vessel trees by transductive inference. *BMC Bioinformatics*. 2014;15:20. 10.1186/1471-2105-15-20.

64. Michail O. An Introduction to Temporal Graphs: An Algorithmic Perspective. In: Zaroliagis C, Pantziou G, Kontogiannis S, editors. *Algorithms, Probability, Networks, and Games: Scientific Papers and Essays Dedicated to Paul G. Spirakis on the Occasion of His 60th Birthday*. Cham: Springer; 2015. p. 308-43.

65. Lobet G, Pagès L, Draye X. A novel image-analysis toolbox enabling quantitative analysis of root system architecture. *Plant Physiol*. 2011;157(1):29-39. 10.1104/pp.111.179895.

66. Schneider CA, Rasband WS, Eliceiri KW. NIH Image to ImageJ: 25 years of image analysis. *Nat Methods*. 2012;9:671-5. 10.1038/nmeth.2089.

67. Pedregosa F, Varoquaux G, Gramfort A, et al. Scikit-learn: Machine Learning in Python. *J Mach*

Learn Res. 2011;12:2825–30.

68. Rampasek L, Goldenberg A. TensorFlow: Biology's Gateway to Deep Learning? *Cell Syst*. 2016;2(1):12-4. https://doi.org/10.1016/j.cels.2016.01.009.

69. Virtanen P, Gommers R, Oliphant TE, et al. SciPy 1.0: fundamental algorithms for scientific computing in Python. *Nat Methods*. 2020;17:261-72. 10.1038/s41592-019-0686-2.

70. Van Der Walt S, Schönberger JL, Nunez-Iglesias J, et al. Scikit-image: Image processing in python. *PeerJ*. 2014;2:e453. 10.7717/peerj.453.

71. Waskom M. Seaborn: Statistical Data Visualization. *J Open Source Softw*. 2021;6(60):3021. 10.21105/joss.03021.

72. Hunter JD. MATPLOTLIB: A 2D GRAPHICS ENVIRONMENT. *Comput Sci Eng*. 2007;9(3):90–5. 10.1109/MCSE.2007.55.

73. Wickham H. ggplot2: Elegant Graphics for Data Analysis. 1st ed. Springer: New York; 2009.

74. Kay M. ggdist: Visualizations of Distributions and Uncertainty in the Grammar of Graphics. *IEEE Trans Vis Comput Graph*. IEEE; 2024;30(1):414-24. 10.1109/TVCG.2023.3327195.

75. Randles BM, Pasquetto IV, Golshan MS, et al. Using the Jupyter Notebook as a Tool for Open Science: An Empirical Study. *Proc ACM/IEEE Jt Conf Digit Libr*. 2017;1-2. 10.1109/JCDL.2017.7991618.

76. CC BY 4.0. https://creativecommons.org/licenses/by/4.0/. Accessed 28 Sep 2025.

77. SeedGerm-VIG Image Series. https://www.ebi.ac.uk/biostudies/bioimages/studies/S-BIAD1852. Accessed 28 Sep 2025.

78. SeedGerm-VIG. https://github.com/The-Zhou-Lab/SeedGerm-VIG/releases. Accessed 28 Sep 2025.

79. Dai J; Wen Z; Ali M; et al. Supporting data for "SeedGerm-VIG: an open and comprehensive pipeline to quantify seed vigour in wheat and other cereal crops using deep learning powered dynamic phenotypic analysis" GigaScience Database. 2025. https://doi.org/10.5524/102764

Markup manuscript

Click here to access/download;Manuscript;Dai et al. V1.15_markup.docx

SeedGerm-VIG: an open and comprehensive pipeline to quantify seed vigour in wheat and other cereal crops using deep learning powered dynamic phenotypic analysis

Short title: AI-powered seed vigour assessment in cereals

Authors:

Jie Dai[1+], DaiJie@stu.njau.edu.cn, orcid: 0000-0002-3941-576X

Zhenjie Wen[1+], WenZhenjie@njau.edu.cn, orcid: 0000-0002-8191-1070

~~Mujiahid~~Mujahid Ali[1+], Mujahid.Ali@stu.njau.edu.cn, orcid: 0000-0001-9239-5705

Jinlong Huang[1], huangjl@stu.njau.edu.cn, orcid: 0009-0003-7332-9915

Shuchen Liu[1], Liushuchen@stu.njau.edu.cn, orcid: 0009-0003-3218-4473

Jianhua Zhao[1], 13770829760@163.com, orcid: 0009-0003-3268-6350

Felipe Pinheiro[2], Felipe.Pinheiro@niab.com, orcid: 0009-0007-4208-3018

Changcai Yang[3], changcaiyang@gmail.com, orcid: 0000-0003-0996-9718

Bin Wang[1], 2023801262@stu.njau.edu.cn, orcid: 0009-0002-2104-0477

Lingzhen Ye[4], yelingzhen@zju.edu.cn, orcid: 0000-0001-6509-9142

Xueying Guan[4*], xueyingguan@zju.edu.cn, orcid: 0000-0002-6528-2518

Ji Zhou[1,2*], Ji.Zhou@njau.edu.cn or Ji.Zhou@NIAB.com, orcid: 0000-0002-5752-5524

[+] Contributed equally, [*] Corresponding authors

[1]College of Engineering, College of Agriculture, Academy for Advanced Interdisciplinary Studies, Plant Phenomics Research Centre, Nanjing Agricultural University, Nanjing 210095, China

[2]Data Sciences Department, National Institute of Agricultural Botany (NIAB), Crop Science Centre (CSC), Cambridge CB3 0LE, United Kingdom

[3]Center for Agroforestry Mega Data Science, School of Future Technology, College of Computer and Information Sciences, Fujian Agriculture and Forestry University, Fuzhou 350002, China

[4]Zhejiang Provincial Key Laboratory of Crop Genetic Resources, Institute of Crop Science, Plant Precision Breeding Academy, College of Agriculture and Biotechnology, Zhejiang University, Hangzhou 310058 China

Corresponding authors

Ji.Zhou@NJAU.edu.cn or Ji.Zhou@NIAB.com; XueyingGuan@zju.edu.cn

Abstract

As one of the most important cereal crops, wheat (*Triticum aestivum* L.) production and grain quality are essential to many nations in the world. Early developmental phases such as seed germination and seedling establishment are key to wheat's growth and development as they impact directly on ~~crop~~crop's early performance and yield potential. Hence, it is critical to develop varieties with favourable early growth characteristics under various growing conditions~~ to sustain early crop performance.~~. Here, we present SeedGerm-VIG, an automated and comprehensive pipeline developed for assessing seed vigour in wheat and other cereal crops. Building on the SeedGerm system, we integrated multiple deep learning models (i.e. YOLOv8x-Germ and optimised U-Net) and computer vision algorithms into the automated seed-level analysis pipeline to identify key germination phases and measure seed-, root-, and seedling-level phenotypic traits. Then, by using time series directed graph,

not only did we track root tips to measure root emergence during the germination procedure (seed-lot ~~level~~ R^2 = 84.1%), but we also established a new approach to examine speed and uniformity of germination. These resulted in the establishment of a vigour scoring matrix, through which 21 commercial genotypes' (i.e. 494 randomly sampled seeds~~;~~, with over 29,500~~+~~ seed-level images) vigour scores were summarised and evaluated at key phases such as protrusion, radicle emergence, and chloroplast biogenesis, which largely ~~matching~~matched with manual assessment based on the International Seed Testing Association (ISTA) guidelines. Finally, we also demonstrated that the SeedGerm-VIG pipeline could be used to assess seed vigour for other cereal crops such as rice (n = 120 seeds) and barley ~~genotypes~~ (n = 240~~) with highly accurate results~~ seeds), reliably. In conclusion, we believe that our work demonstrates a valuable step forward to enable the broader plant and crop research community to examine seed vigour and vigour-related features in an automated ~~and quantifiable~~ manner, facilitating effective and reproducible plant selection and relevant seed science research for crop improvement ~~under a rapidly changing global climate~~.

Formatted: Font: Italic

Keywords:

Seed vigour, germination, vision-based deep learning, dynamic trait analysis, wheat

Introduction

The imminent challenges of climate changes, growing population, and fertiliser shortage have brought diverse threats to global food security [1]. As one of the most consumed cereal grains in the world, wheat (*Triticum aestivum* L.) production and grain quality are vital to many nations in the world [2,3]. Early developmental phases such as seed germination (growth stage, GS 00-09) and seedling establishment (GS 10-19) are particularly critical for wheat growth and development as low-quality establishment often translates into: (1) a reduced plant density and thus lower yield production, (2) decreased crop effectiveness when competing against weeds, and (3) the potential development of early-stage plant diseases [4–6]. Hence, better seed performance at phases such as protrusion, radicle emergence, seedling establishment is likely leading to improved crop health and performance, ensuring yield potential under field conditions [7,8].

In general, the ~~sum~~ combination of favourite germination and seedling establishment characteristics under varied growing conditions is defined as high seed vigour by the International Seed Testing Association (ISTA) [9]. As a complex trait, seed vigour is not only an critical aspect of seed quality [10,11], but also key for breeders and researchers to genetically improve plants [7,12]. Due to the importance of high-vigour features such as seed production capability and seed longevity under various storage conditions, seed vigour is widely accepted as an important subject in seed science research and crop improvement, forming the foundation of modern crop breeding, cultivation, agronomic management, and crop production [7,12,13].

Traditionally, ~~to~~ assessing seed vigour involves many tests and experiments based on physiological and biochemical parameters of seed lots. For example, one popular method is to evaluate cumulative germination rates to identify high or low vigour groups of seed lots [14]. For such experiments, radicle

length at specific time-points (e.g. 48 hours after imbibition) were measured, through which the effectiveness of germination and seed viability was estimated [15]. Other methods such as accelerated aging [16], electrical conductivity [17], and seedling emergence [18] were also used, leading to the classification of different vigour groups. Recently, biochemical markers were employed to study vigour, including: (1) sugar content (e.g. glucose and fructose) as sugars can negatively impact on germination [19]; and (2) protein content because it correlates with better seed performance [20]. Spectrometric reflectance and gas chromatography were also introduced to the research domain: (1) spectrometric reflectance collected by multi- and hyper-spectral sensors and spectroscopies was applied to classify high- and low-performing seed lots [21,22]; (2) gas chromatography was employed to estimate seed vigour groups based on volatile organic compounds released during seed metabolism [23].

Among the above approaches, the use of morphological attributes of seeds (e.g. size and shape), radicles (e.g. length), and seedlings (e.g. establishment timing and rates) provides direct evidence in determining seed vigour. For instance, larger seeds were reported to have a higher vigour potential due to greater nutrient reserves [24]; the physical structure of seed coat can influence water uptake during imbibition and thus affect the speed of seed germination [25]. Additionally, phenotypic variations of germination- and vigour-related features can lead to genetic studies of seed vigour, enabling assessment of seed quality and thus crop performance to accelerate crop breeding [26,27].

Since the 21st century, advances in remote sensing, computer vision (CV), and deep learning (DL) technologies open a new door for evaluating germination- and vigour-related traits or aspects [11]. Using colour, spectral and morphological traits, diverse vision-based solutions were introduced, including: (1) *PhenoSeeder* [28] and *SeedExtractor* [29] developed to analyse size- and colour-based traits based on red-green-blue (RGB) seed imagery; (2) *Germinator* [30] and *SeedQuant* [31] built to

identify germination status using colour and contrast features; (3) *SeedGerm* [32] applied supervised machine learning (ML) to quantify cumulative germination rates for plant species such as tomato, pepper, and Brassica; (4) RootNav 2.0 [33] used DL techniques to measure root features for wheat, *Arabidopsis*, and Brassica; (5) hyperspectral imaging and DL models were combined to estimate oil composition [34] and chemical components (e.g. amino acids and lipids) to study seed quality [35].

Still, many of the above methods focus on measuring traits at a specific time~~-~~point (e.g. protrusion or when radicles reach a certain length), which missed the dynamic nature of the early developmental phase as seed performance can ~~differ~~ fluctuate during germination, particularly when seeds are interacting with external stimuli [36]. After imbibition, radicles play a vital role in absorbing resources and supporting seedling establishment, enabling the active transition phase between heterotroph and photoautotroph [37,38]. Hence, the ability to dynamically measure germination-related phenotypic changes at the seed level (e.g. changes of radicle length) will facilitate plant researchers and breeders to quantitatively examine seed performance, so that seed vigour can be evaluated, objectively and comprehensively.

Here, we present SeedGerm-VIG, an open analytic pipeline developed to assess seed vigour for wheat and other cereals such as rice and barley. Building on the SeedGerm platform previously reported [32], we performed time-lapse imaging of wheat seed germination (for 4-7 days, depending on treatments), followed by the application of SeedGerm-VIG to: (1) identify seed-level germination phases using the YOLOv8x-powered [39] DL model; (2) detect seeds and seedling (e.g. coleoptile) based on an optimised U-Net model [40], through which seed-level morphological features such as seed area, perimeter, length, width and roundness, seed coat colour, and the seedling system could be measured; and (3) track positions of radicles, lateral roots, and seedling tips over time to pinpoint their

emergence rates. Finally, utilising timepoints and growth rates of protrusion, radicle emergence, and chloroplast biogenesis (when seedling turned green), we successfully established a vigour scoring matrix to quantify this complex trait for 21 commercial genotypes at key developmental stages during germination, whose results were partially compatible with the ISTA's guidelines [15].

Findings

Time-lapse seed germination imaging

We first established many low-cost SeedGerm devices [32] to perform RGB overhead time-lapse imaging of seed germination with high-definition (HD) seed-level images (**Fig. 1a**). Experiments were conducted in translucent plastic boxes, with either second-hand smartphones (with a maximum of 3,840 × 2,464 pixels per image) or *Raspberry Pi* image sensors (with a maximum of 4,608 × 4,608 pixels per image) mounted on the top (**Fig. 1b**). A series of RGB images were acquired with one-hour intervals between each shot, recording from dry seeds and imbibition to seedling establishment after the chloroplast biogenesis phase (**Figs. 1c&d**). In total, 21 commercial wheat genotypes known for different germination paces were selected.

To improve the generalisation of the SeedGerm-VIG pipeline, we trialled different experimental settings such as different seeds (e.g. 18, 20, 25, or 30 seeds) and blue or black germination papers (**Fig. 1c**). Depending on plant species, treatments (e.g. cold stratification and ambient temperatures), and potential germination speeds, time-lapse imaging was set from 96 hours (i.e. 4 days) to 168 hours (i.e. 7 days), with HD seed-lot image series (i.e. RGB images acquired in the experiments) stored and synchronised via OneDrive (**Table S1**).

A high-quality training dataset for assessing seed vigour

A seed-lot image series normally covers the entire germination procedure until seedling was fully established (**Fig. 1d**, lower). For the 21 experiments performed in this study, a total of 1,890 seed-lot images were collected. To enable DL model to identify germination phases for every seed in a given experiment, plant specialists first used LabelMe [41] to annotate germination phases based on seed-level images (**Fig. 1d**, upper), including 250 images at imbibition (IMB), 218 at protrusion (PRO), 278 during radicle emergence (RE), and 215 during seedling establishment (SE). These images were randomly selected from 48 seed-lot images with either black (19) or blue germination papers (29).

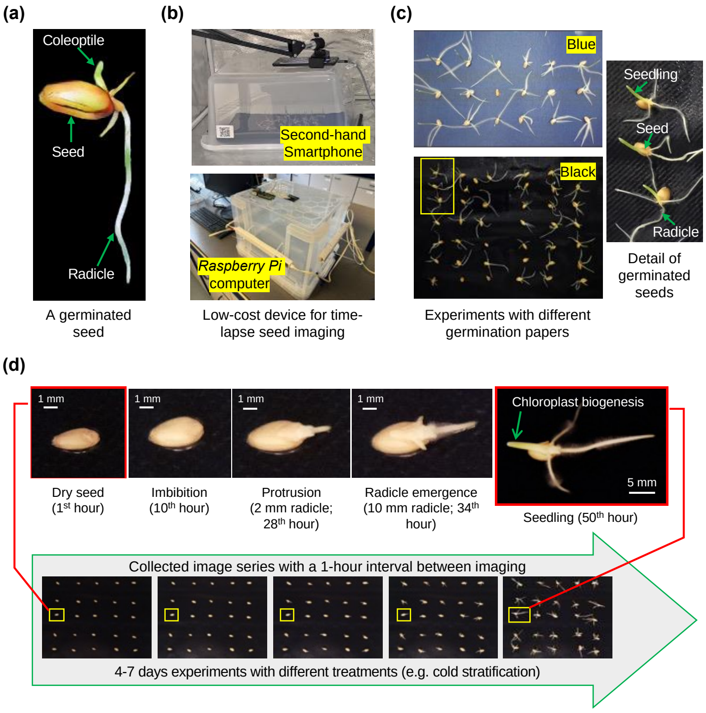

Figure 1. Time-lapse seed germination imaging and acquired image series using SeedGerm devices. (Note: detailed legends are provided in the Figures section).

After image augmentation (e.g. adding noise and image flipping; see **Methods**), an augmented training set called "SeedVig-phase" was established, which contained 3,053 images, relatively evenly distributed across four key germination phases (i.e. 776 images at IMB, 720 at PRO, 784 during RE, and 773 during SE). Additionally, to perform background removal for accurate seed-level trait analysis, more images were annotated, including 644 seeds without roots and seedlings, 702 seeds with roots and seedlings, and 680 seedlings. Similarly, image augmentation was applied and created a training set called "SeedVig-traits", consisting of 1,932 seeds, 2,106 seeds with roots and seedlings, and 2,040 seedlings. The two evenly distributed training sets (**Fig. S1**) were both used in developing and benchmarking DL models.

The YOLOv8x-Germ model for identifying key germination phases

To automate the identification of key germination phases at the seed level in ~~a given~~ seed~~-~~lot image series, we developed a YOLOv8x-powered model called "YOLOv8x-Germ" using the "SeedVig-phase" training set, which was then embedded in the SeedGerm-VIG pipeline (**Fig. 2a**, upper). Seed-level bounding boxes were generated with colour coding to indicate different germination phases (i.e. red, blue, green and orange for IMB, PRO, RE and SE phases, respectively), with confidence levels (0-1, with two decimal places retained) attached to signify the probability of the DL-based estimation (**Fig. 2a**, lower). The YOLOv8x-Germ model was used to identify seed-level germination phases and regions of interest (ROIs) in any seed-lot images collected under dissimilar conditions (**Fig. 2b**). According to the ISTA guideline [9], a specific germination phase is determined when at least 75% of seeds in a given seed lot reach the phase. We therefore used the unseen data in the "SeedVig-phase" dataset to evaluate the DL model's performance when it was used to identify key germination phases for all the seeds in a

seed lot. This resulted in highly accurate identification of germination phases at the seed-lot level, from IMB (100.0%) and SE (100.0%) to PRO (90.0%) and RE (83.3%) phases (**Fig. S2**, right). To standardise DL-based phase identification for experiments with different numbers of seeds, 15 seeds from all the experiments (i.e. seed lots) were sampled (**Fig. S3**).

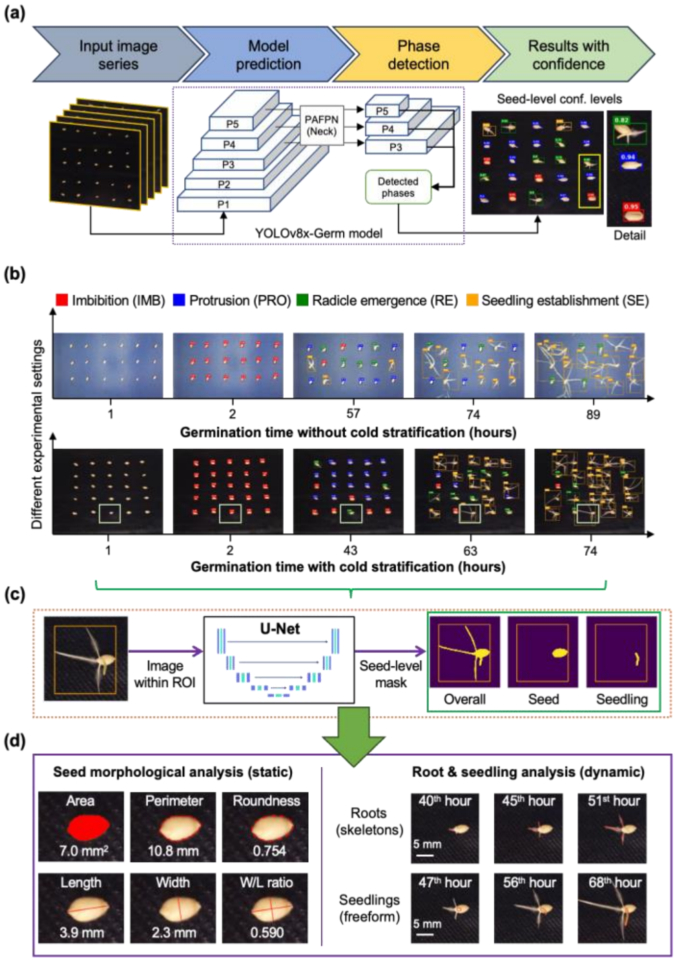

Figure 2. The analysis workflow of SeedGerm-VIG for identifying key germination phases for every seed in a seed lot, followed by the measurement of seed-level static and dynamic germination traits.

U-Net powered seed-level trait analysis and evaluation

To analyse germination- and vigour-related traits, after identifying seed-level bounding boxes (i.e. ROIs) of all the seeds in a seed lot, we developed a U-Net based model to precisely remove background objects (i.e. black and blue germination papers, and root hairs) within the ROIs (**Fig. 2c**). Using a seed in Genotype 7 (G7; Row 5, Column 3; highlighted in **Fig. 2b**), the U-Net model enabled us to retain seed, radicle, lateral roots, and seedling within the ROIs. Then, seed colour and morphological traits such as seed coat colour, area, perimeter, roundness, length, width, width and length (W/L) ratio were quantified to examine dry and imbibed seeds (**Fig. 2d**, left), followed by the measurement of root- (both radicles and two lateral roots) and seedling-related features such as root length (using two dimensional, 2D, skeletons, seedling size and colour; **Fig. 2d**, right). **Table 1** summaries all the static and dynamic traits quantified by the SeedGerm-VIG pipeline at three germination phases.

Table 1. The summary of traits quantified at three germination phases.

Germination phases	Static traits	Dynamic traits
**Imbibition (IMB) & protrusion (PRO)**	Seed area (mm2)	Change rates of seed area (%)
	Seed length (mm)	Change rates of seed length (%)
	Seed width (mm)	Change rates of seed width (%)
	Seed perimeter (mm)	Change rates of seed perimeter (%)
	Seed W/L ratio (0-1)	
	Seed roundness (0-1)	
	Seed coat colour Red (0-255)	
	Seed coat colour Green (0-255)	
	Seed coat colour Blue (0-255)	
	~~Time point~~Timepoint of PRO (seed level; hour)	
	~~Time point~~Timepoint of PRO (seed lot level; hour)	
**Radicle emergence (RE)**	Radicle length (mm)	Change rates of radicle length (mm/h)
	Root 2 length (mm)	Change rates of root 2 & 3
	Root 3 length (mm)	

	~~Time point~~Timepoint of RE (seed level; hour)<br>~~Time point~~Timepoint of RE (seed lot level; hour)	length (mm/h)
**Seedling establishment (SE)**	Seedling length (mm)<br>~~Time point~~Timepoint of coleoptile emergence (hour)<br>~~Time point~~Timepoint of Chloroplast biogenesis (hour)<br>~~Time point~~Timepoint of SE (seed level; hour)<br>~~Time point~~Timepoint of SE (seed lot level; hour)	Duration from coleoptile emergence to chloroplast biogenesis (seed level; hour)

Computationally derived traits were evaluated against manual scoring using correlation analyses, resulting in significant correlations for these traits ($P < 0.001$; **Fig. S4**), including seed length (R^2 = 0.894), seed width (R^2 = 0.859), seed perimeter (R^2 = 0.881), seed area (R^2 = 0.881), radicle length (R^2 = 0.800), and seedling size (R^2 = 0.791), which indicated the reliability of the SeedGerm-VIG pipeline in phenotypic analysis. Moreover, we compared traditional germination traits scored manually and the SeedGerm-VIG-derived trait analysis, including germination potential (GP) on the first, second, and third day (i.e. Day 1, 2 and 3) of the experiment (i.e., GP_1, GP_2, GP_3), germination index (GI_1, GI_2, GI_3), mean germination time (MGT), and time to 50% germination (T_{50}). The correlation analyses between the 8 traditional and SeedGerm-VIG-derived traits range from 0.728 to 0.910 (**Fig. S5**), demonstrating the reliability of the SeedGerm-VIG pipeline. Additionally, we computed the broad-sense heritability for the SeedGerm-VIG-derived traits, estimating the genetic influence on them (~~**Table**~~**Tables S2-S4**).

A graph-based tracking method to study root emergence

To quantify timing, duration, and growth rate of root emergence (including radicle and lateral roots) for

a seed lot during the germination procedure, we utilised 2D radicle skeleton (**Fig. 2d**, upper right) to derive dynamic traits (e.g. germination speed and phase-based uniformity) for assessing seed vigour. A temporal directed graph was established to track radicle and lateral root tips for all the seeds in a given experiment. Using a given seed in G7 (Row 1, Column 2; **Fig. 3a**), the graph recorded 2D coordinates and growth direction of every primary root (i.e. radicle) and lateral root tips emerged from the seed before root intersection, including: (1) radicle and lateral root tips' coordinates and positional changes within 5-hour periods, (2) the distance and growth direction of the same root tip (red-coloured skeletons and blue-coloured growth distances, in mm; **Fig. 3a**), and (3) cumulative root growth (yellow-coloured skeletons, in mm; **Fig. 3a**). Detailed algorithmic explanation is given ~~in the~~ (**Methods**~~ and~~; **Figure S6**~~.~~).

When root intersection happened (lower, **Fig. 3a**), the root skeletons were extracted within root intersection regions (highlighted with a light-green-coloured dotted rectangle; **Fig. 3b**). Intersected points, angles and starting points of root skeletons, and the most likely growth direction (i.e. based on the previously recorded root graphs) were determined (**Fig. S7**, left and middle), enabled us to associate root skeletons with their corresponding seeds within intersection regions. The above algorithmic steps were applied to track root tips throughout the germination procedure (**Fig. S7**, right). For example, by tracking roots of 25 seeds (i.e. G7), we visualised the tracking result at the 80th hour, where root skeletons were pseudo-coloured according to time (i.e. 0-100 hours, from dark blue to dark red), followed by the measurement of seed-level root emergence based on assembled root skeletons. Noticeably, root skeletons did not exactly match with the 80th hour image because they were assembled during time when roots were slightly moved during the experiment (**Fig. 3b**). To ensure that the SeedGerm-VIG work could reach the broader plant research community, we created a graphical user interface (GUI) to include the above algorithmic steps using widget, so that nonexperts can use the

pipeline relatively easily (see **Methods**).

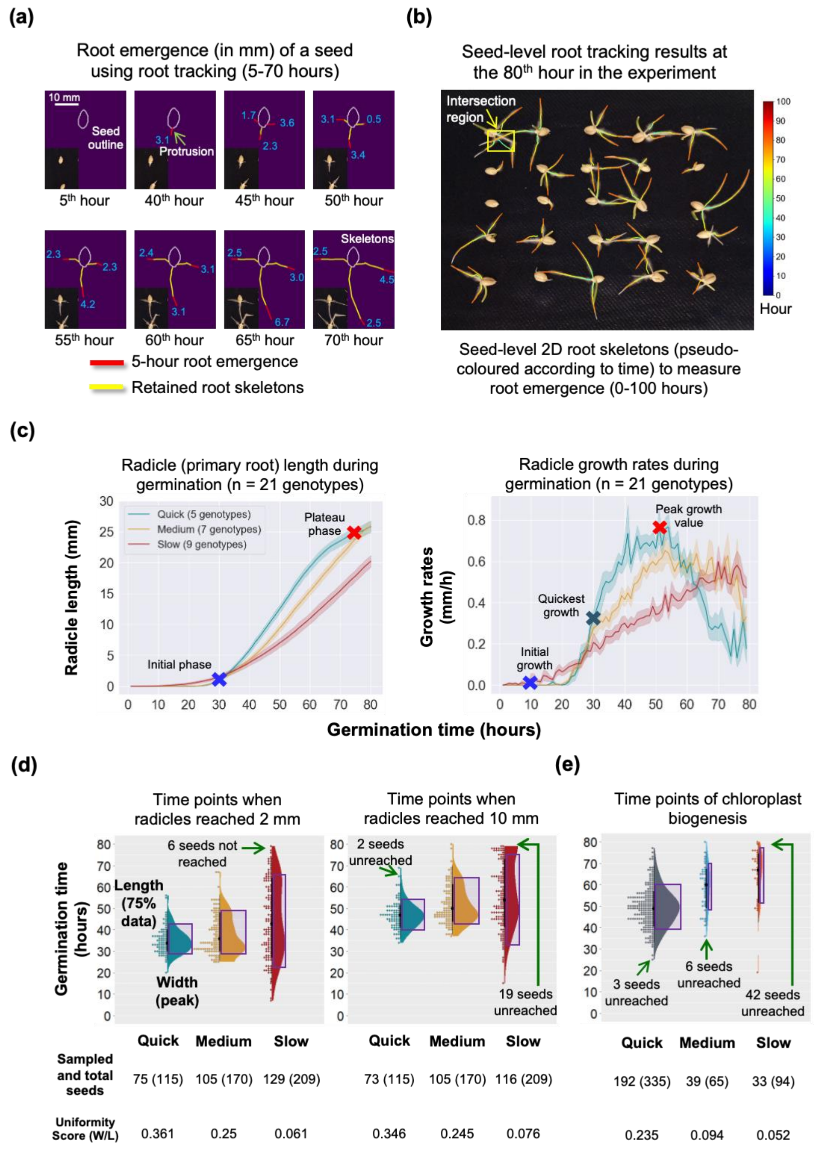

Figure 3. Tracking radicle and lateral root tips, growth speed profiles, and uniformity analysis during key germination phases.

The growth patterns of radicles

Using the root tip tracking algorithm, we examined the 21 genotypes and their radicle emergence characteristics. According to the ISTA guideline [15], when the length of a radicle reaches 2 mm, the seed is "germinated". When a seed's radicle length reaches 10 mm, the seed's root system is "established" [42,43]. In our case, by quantifying radicle length at different ~~time points~~timepoints with 1-hour intervals when radicles emerged to 10 mm (roughly between 0 and 80 hours), we were able to calculate 1-hour growth rates for all the genotypes, leading to 21 radicle growth profile curves. All the genotypes followed a gradual increase pattern, with varying growth speeds (**Fig. S8**). Using the radicle length as a metric, we applied the agglomerative clustering method [44] to categorise the 21 curves (**Table S5**~~), grouping them~~) into three categories: Quick (5 genotypes; teal coloured), Medium (7 genotypes; orange), and Slow (9 genotypes; dark red) groups (**Fig. 3c**, left). The differences between the three groups occurred at around 30th hour (highlighted with a blue-coloured cross) when seeds were entering protrusion. The Quick group's radicles began to slow down at 75th hour (highlighted with a red-coloured cross). Noticeably, quick root emergence did not necessarily lead to the longest radicle length as the Medium group's radicle length was slightly longer than those in the Quick group.

Moreover, we studied radicle growth rates of the 21 genotypes, demonstrating varied patterns (**Fig. 3c**, right): (1) growth rates did not ~~follow~~increase in a gradual~~ and smooth increase~~ pattern, instead they fluctuated throughout the 80-hour period; (2) timings of highest growth rates of the three groups differed, with the Quick group (teal) peaked at around 50th hour (denoted with a red-coloured cross), followed by around 54th and 70th hours for the Medium and Slow groups; (3) all the groups experienced declines after the peak, with a sharp decline observable from the Quick group; (4) the Slow group (dark red)

exhibited an early initial growth (denoted with a blue cross), the smallest peak, and a relatively gradual increase trend compared with the other groups; (5) by finding the first derivative of the growth rate curve, we were able to locate the ~~time point~~timepoint when radicle growth was changing at the quickest rate (indicated with a dark-teal cross), suggesting the most active phenotypic changes of radicle length. For the above curves (**Fig. 3c**), shaded regions were provided to represent the confidence interval (75%).

The uniformity scoring of vigour-related features

Building on the measures of growth patterns (i.e. germination speed), we further studied how to derive uniformity of the seed lots during the germination procedure. For the two radicle emergence stages (i.e. 2 mm for germination and 10 mm for root system establishment), we employed raincloud plots (the x-axis shows the three growth groups) to present the distribution of ~~time points~~timepoints (y-axis) when seed lots entered germination and root establishment (**Fig. 3d**): (1) the Quick group (teal) had a narrow and concentrated distribution, with most values clustered around 30–40 hours; (2) the Medium group (orange) had a broader and more stretched distribution, with a central tendency around 30–50 hours; and (3) the Slow group's (dark red) data distribution was the widest and most spreading, with values covered across the y-axis and some seeds did not reach target radicle lengths (i.e. 2 and 10 mm, indicated with green-coloured arrows). Because the three groups had dissimilar ranges and centred values when radicle reached the two lengths, we therefore computed the ratio of the peak value (when most of the seeds reached 2 and 10 mm) and the coverage of 75% data (highlighted with purple-shading rectangles in **Fig. 3d**), resulting in a uniformity score (0-1) measuring how similar a given germination speed group elongated during different phases. This enabled us to effectively differentiate the three groups in terms of the RE uniformity, showing genotypes belonging to the Quick group had higher uniformity

scores (i.e. 0.361 and 0.346 at the two phases) compared to the genotypes in Medium (around 30% less than the Quick group) and Slow (over 80% less) groups.

Similarly, using the timepoints when chloroplast biogenesis occurred (seedling turned green; **Table S6**), we applied affinity propagation clustering [45] to classify the genotypes ~~into three categories~~without pre-defining cluster number, followed by the analysis of their data distribution and uniformity (**Fig. 3e**). Three categorises were identified. While the Quick group (gray) exhibited the broadest distribution and the Slow group (light red) the narrowest, genotypes in the Quick group had a higher uniformity score (0.235) compared to those in the Medium (light blue; around 60% less) and Slow (over 70%) groups. Additionally, using the raincloud plots (**Figs. 3d&e**), we identified seeds that did not reach a certain phase. For example, 6 (out of 129) and 19 (out of 116) seeds in the Slow group did not reach 2 mm and 10 mm radicle lengths, whereas the majority of the seeds in the Quick and Medium groups established the root system successfully. As for the chloroplast biogenesis, the Slow group had 42 (44.7%) seeds did not reach this phase during the 80-hour monitoring period compared with only 3 seeds (0.9%) in the Quick group and 6 seeds (9.2%) in the Medium group.

A comprehensive assessment of seed vigour

Traditional seed vigour assessment often missed the dynamic nature of this complex trait as its performance could vary throughout the germination procedure. Hence, to facilitate an efficient and reproducible method to assess seed vigour, we created a comprehensive matrix to incorporate germination speed and uniformity at different germination phases into the assessment of seed vigour (**Fig. 4**). Using the phase-based classification of growth patterns (i.e. speed) and associated uniformity scores computed for the 21 genotypes (**Tables S7 & S8**), we categorised the genotypes into three groups

during the PRO, RE and SE phases using the affinity propagation method [45].[45]. Then, we set the Quick group as 3 points, Medium as 2 points, Slow as 1 point and multiplied them with their uniformity scores (i.e. Speed × Uniformity), which resulted in three overall vigour groups: (1) five Low-vigour genotypes (G1, G2, G3, G18 and G19), (2) 11 Medium-vigour genotypes (G7, G9, G10, G12, G13, G14, G15, G16, G17, G20 and G21), and (3) five High-vigour genotypes (G4, G5, G6, G8, and G11). We compared the above vigour results with traditionally assessed vigour groups (i.e. 5 Low-vigour genotypes and 16 High-vigour genotypes; **Table S9**) and found that the manually scored Low-vigour (n = 5) and High-vigour (n = 5) genotypes matched with the SeedGerm-VIG derived lines, while the Medium-vigour group could not be assessed as manual scoring could not identify genotypes with this feature.

Genotypes	PRO phase (< 2 mm)		RE phase (2-10 mm)		SE phase (chloroplast biogenesis)		Overall vigour
	Speed	Uniformity	Speed	Uniformity	Speed	Uniformity	
G1	Slow	0.03	Slow	0.04	Slow	0.01	0.08
G2	Slow	0.02	Slow	0.01	Slow	0.01	0.04
G3	Medium	0.02	Slow	0.03	Slow	0.01	0.08
G4	Quick	0.31	Medium	0.29	Quick	0.04	1.63
G5	Quick	0.59	Quick	0.21	Quick	0.04	2.53
G6	Quick	0.23	Medium	0.22	Quick	0.10	1.43
G7	Medium	0.10	Medium	0.09	Quick	0.08	0.61
G8	Quick	0.33	Medium	0.30	Quick	0.10	1.90
G9	Quick	0.12	Slow	0.05	Quick	0.01	0.45
G10	Quick	0.14	Quick	0.09	Quick	0.04	0.80
G11	Quick	0.69	Medium	0.46	Quick	0.17	3.51
G12	Quick	0.16	Quick	0.09	Quick	0.02	0.82
G13	Medium	0.13	Medium	0.14	Medium	0.03	0.59
G14	Medium	0.04	Slow	0.04	Quick	0.02	0.19
G15	Quick	0.13	Slow	0.11	Quick	0.09	0.78
G16	Quick	0.02	Slow	0.02	Quick	0.02	0.13
G17	Medium	0.05	Medium	0.04	Medium	0.01	0.19
G18	Slow	0.02	Slow	0.01	Slow	0.01	0.04
G19	Slow	0.02	Slow	0.07	Slow	0.01	0.09
G20	Quick	0.04	Quick	0.02	Quick	0.01	0.19
G21	Quick	0.11	Quick	0.12	Medium	0.01	0.69

■ Slow speed or low uniformity ■ Medium speed or medium uniformity ■ Quick speed or high uniformity

Figure 4. A comprehensive matrix provides an overview of 21 wheat genotypes' germination speed groups (pseudo-coloured) and uniformity scoring (with two decimal points) at three key phases, followed by the computation of overall seed vigour scores based on measures of phase-based speed and

uniformity for the 21 genotypes.

Discussion

As a critical and complex agronomic trait in cereals, seed vigour demonstrates plant seeds' ability to germinate, establish seedlings, and sustain early growth under varied growing conditions [7]. As seed vigour is governed by multiple genetic, biochemical, and physiological factors [14], it is difficult to conduct a comprehensive study through traditional approaches. Still, because high-vigour seeds often lead to uniform crop establishment and thus reduced risks of crop performance (e.g. poor establishment and seedling mortality) under field conditions, breeders and researchers are keen to study and incorporate this trait in the breeding programmes to sustain crop production [46]. Moreover, seed vigour is also treated as a vital target for developing climate-resilient crops, providing plants with a competitive edge against external stimuli at early developmental phases [47]. As previous studies on seed vigour often focus on germination-related traits measured at specific timepoints, which missed the dynamic and complex nature of seed vigour [26], we therefore developed the SeedGerm-VIG methodology, an open and automatic analytic pipeline for quantifying seed-, root- and seedling-related traits and their phenotypic changes during key germination phases, through which we could empower the research community with a new toolkit to assess seed vigour in a comprehensive and dynamic approach. We trust that our toolkit has made advances in a number of areas for seed science research.

The SeedGerm-VIG pipeline for assessing vigour-related traits

Due to recent advances in vision-based DL techniques, DL solutions have been widely applied to plant phenotyping related studies [48]. Using the SeedGerm platform for time-lapse imaging, we integrated

two trained DL models (i.e. YOLOv8x and U-Net) into the SeedGerm-VIG pipeline to analyse time-series images collected in dissimilar experimental settings (e.g. the number of seeds, seed treatments, and germination papers), which outperformed several classical and latest DL models (**Tables 2&3**). This enabled us to establish an automated approach to quantify seed-, root-, and seedling-related traits. For example, the YOLOv8x-Germ model was trained to perform the seed-level identification of key germination phases (i.e. IMB, PRO, RE, and SE) for every seed in a seed lot (**Fig. 2b**), which helped us identify seed-level germination progress and facilitate the segmentation of seed-level ROIs for the following trait analysis. With limited training datasets (3,053 seeds), the YOLOv8x-Germ model achieved high-accuracy predictions of the IMB (84.7%), SE (89.5%), PRO (77.0%), and RE (84.1%) phases based on 1,207 seeds (**Figs. S1&S2**), which can be easily improved when more training datasets are made available through our openly accessible pipeline.

Additionally, within the seed-level ROIs, we used an optimised U-Net model to generate masks of seeds with or without roots and seedlings (**Fig. 2c**), leading to the analysis of seed-level morphological features, root (both radicle and lateral roots) length and tips, and seedling size. According to the correlation analyses (**Fig. S4**), most of the computationally derived traits were significantly correlated with the manual scoring, demonstrating the reliability of the pipeline. Notably, by combining trait analysis with graph-based root tip tracking, we further developed dynamic measures of vigour-related traits by quantifying cumulative root length based on tracking the emergence of root on an hourly basis (**Fig. S9**), through which radicle growth profiles were produced to illustrate the 21 genotypes and their germination speed groups (**Fig. S8**). This also led to the curves of radicle growth rates, providing insights into radicle growth patterns during germination, from 0 to 80 hours.

In particular, we employed a range of computational traits to collectively describe seed vigour,

including: (1) by using seed size during IMB to assess the speed of imbibition (**Fig. S10**); (2) by quantifying radicle and lateral root emergence (**Fig. S11**) to quantify timepoints and duration of PRO and RE phases; (3) by monitoring the presence of greenness on seedlings to obtain the timing of chloroplast biogenesis. Equipping with the above measures, we were able to dissect seed vigour, a highly complex and variable trait, into vigour-related sub-traits to study the trait comprehensively. Compared with our previously published *SeedGerm* platform [32] that was developed for seed-lot level germination analysis, our study partially inherited its hardware design and seed-lot level source codes for automated image processing. Still, for assessing temporal seed-level analysis, instead of traditional ML models, the SeedGerm-VIG pipeline incorporated DL models and graph-based computer vision algorithms to improve the generalisation and accuracy, enabling seed-level trait analysis and vigour-related feature assessment in acquired image series.

The assessment of seed vigour using germination speed and uniformity

In the context of crop improvement, seed vigour can help us select genotypes that are more resilient under different environment and aging conditions [7]. How to assess vigour-related features when seeds are interacting with external stimuli can provide us with insights into the selection of varieties with better crop establishment, productivity, and adaptability in diverse agricultural settings [46]. In our study, besides quantifying timing and speed to evaluate seed performance over time, we also explored the calculation of uniformity, which is another favourite high-vigour feature advised by ISTA [49]. We used raincloud plots to exhibit the data distribution when seeds entered the PRO, RE, and SE phases. While the timepoints of seeds entered a key phase can be used to assess the germination speed and hence the cumulative germination rates, the raincloud plots also assisted us to examine the data distribution of

how many seeds entered a given phase over time. For example, using 75% of seeds when they entered a germination phase as “length” and the peak value of the data distribution as “width” (**Fig. 3d**, left), we computed the ratio of the width and the length (i.e. W/L) that was also proven to be reliable using heritability~~ and~~, coefficient of variation, and interquartile range (IQR) (**Tables S2-S4~~ &~~, S10, S11**), deriving a metric (0-1) to represent the uniformity of radicle emergence and chloroplast biogenesis. The metric could ~~nicely differentiate~~effectively distinguish dissimilar uniformity patterns for the 21 wheat genotypes, as well as the three germination speed groups. Finally, we proposed a seed vigour matrix to incorporate germination speed and uniformity at the three phases into the identification of overall seed vigour, resulting in 5 High-vigour, 11 Medium-vigour, and 5 Low-vigour genotypes, which largely correlated with the manual assessment following the ISTA guideline. To our knowledge, we originated this method to advance automated seed vigour assessment, integrating germination speed and uniformity to identify high-performing seed lots quantitatively.

The application of SeedGerm-VIG for other cereal crops

The SeedGerm-VIG pipeline enabled us to perform germination- and vigour-related trait analysis across the germination procedure, facilitating us to examine desired characteristics such as rapid germination, efficient establishment of root and seedling systems, and the smooth transition from root establishment to photosynthetic autotrophy under varied growing conditions (e.g. cold stratification and different temperatures). To study the generalisation of the proposed method, we further applied the SeedGerm-VIG pipeline to test other cereal crops such as barley and rice ~~(**Fig. 5**).~~through transfer learning [50], so that the pre-trained DL models could be quickly fine-tuned with significantly reduced epochs and smaller training sets, indicating the potential use of DL techniques for multi-species studies. For barley,

we carried out experiments with dark red germination papers for twelve commercial lines (i.e. barley genotype, BG; 20 seeds per line) ~~together with time-lapse imaging of 93~~for 90-95 hours (**Fig. 5a**, left).

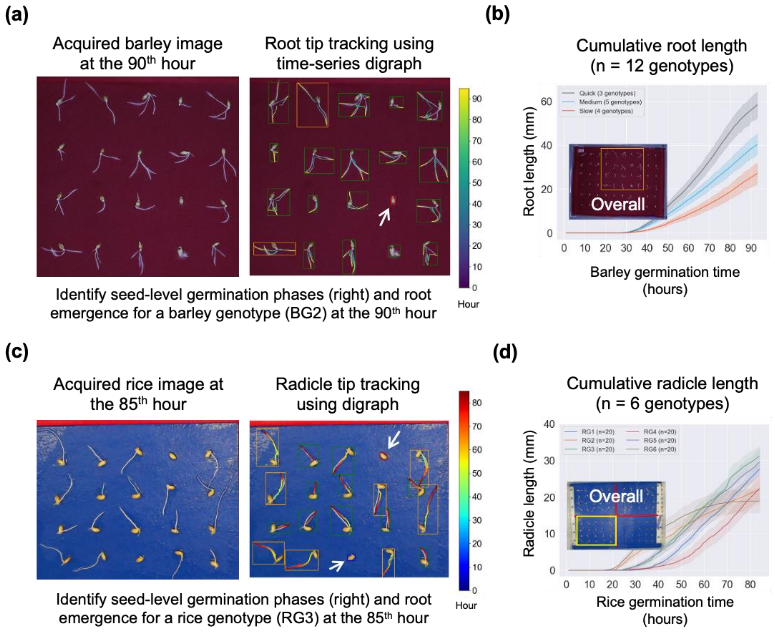

Figure 5. The application of the SeedGerm-VIG pipeline to perform seed-level analyse of root (barley) and radicle (rice) emergence for 12 commercial barley and six rice genotypes.

For example, the YOLO8s-Germ identified germination phases at the 90th hour with coloured bounding boxes, highlighting an ungerminated seed (Column 4, Row 3; enclosed with a red box), which was still at the IMB phase (**Fig. 5a**, right). Then, the graph-based root tracking algorithm was applied to assess seed-level barley roots, generating pseudo-coloured root skeletons that represented root emergence time (**Fig. 5a**, right). Similar to wheat experiments, we computed cumulative root length of the twelve genotypes using 15 sampled seeds, clustering three speed groups for the barley lines (**Fig. 5b**; **Table** ~~S11~~S12). For rice, SeedGerm-VIG was applied to process six commercial rice genotypes

(RG) trialled on light blue germination papers, identifying seed-level germination status at the 85th hour (with one seed ungerminated and one seed did not reach the PRO phase, both of which were highlighted with coloured bounding boxes), with tracked rice radicels and thus RE speed groups (**Figs. 5b&d**).

Additionally, we performed correlation analyses between computationally derived traits and manually scored radicle length for 12 barley (R^2 >= 0.793; P < 0.001) and six rice genotypes (R^2 = 0.815; P < 0.001), demonstrating the reliability and generalisability of the SeedGerm-VIG pipeline for other cereal plants (**Figs. S12 & S13**). The uniformity scores also computed, which were closely corresponded to the germination patterns across all the three speed groups for barley when the radicles reached either 2 or 10 mm (**Fig. S12b**), confirming the practical value of the vigour index.

Limitations and future developments

Building upon the ISTA guidelines, this study advanced the assessment of seed germination and vigour from seed lots to the seed level, which empowered us to study seed-level performance during the germination. Still, we have encountered several limits for the SeedGerm-VIG pipeline~~, which~~ when suboptimal imaging conditions, root occlusions, and excessive seed or root movements were witnessed (**Note S2**). Some cases could be improved in ~~the~~ future studies, including: (1) image quality, which could be affected at early phases due to reflection on germination paper surface as too much water was applied; this issue could be mitigated by controlling water applications at different timepoints; (2) complex root intersection, which could lead to low accuracies of root emergence measures during RE and SE phases; this might be rectified by more labelled training data, cutting-edge DL models, and focusing on radicles with more frequent overhead imaging (e.g. 10-minutes per shot when radicle tip changes are limited); (3) seed and root movement, which complicated seed positions and root tip

tracking (see the algorithmic steps in **Note S1**); how to prevent seeds from excessive movement (e.g. 3D printed seed holders) could be considered; (4) the generalisation of the solution, which can be continuously improved with more wheat, barley, and rice genotypes included in the training dataset and thus the refined DL models; (5) the detailed requirements outlined in ISTA's guidelines (e.g. distinguishing between normal and abnormal seedlings), which will be considered ~~in future developments as they can improve the practicality of our pipeline.~~as they can improve the practicality of our pipeline. Additionally, due to the limitation of 2D photography, low-cost devices such as smartphones mounted on turntables can be considered to reconstruct 3D colour point clouds [51], based on which seed germination and vigour can be assessed more comprehensively. Notably, due to the open-source nature of our work, we believe that a community-driven effort could help verify and improve the performance and accuracy of the seed vigour assessment based upon the SeedGerm-VIG pipeline~~.~~ and its easy-to-use GUI.

Besides the above limits, it is valuable to point out that the study presented here is likely opening up new opportunities for us to reveal the genetics of seed vigour, which involve multiple genes and regulatory networks that influence the physiological and biochemical processes. Rapid germination, uniform root and seedling growth, stress tolerance, and even seed longevity are controlled by the interactions of genetic factors and external stimuli. Static and dynamic sub-traits presented in this study can be used to gain an in-depth understanding of the genetic basis of seed vigour, including: (1) genetic components of seed vigour that regulate seed metabolism, seed dormancy and germination, biotic and abiotic stresses, and seed coat and structural features; (2) genetic mapping to identify genetic regions associated with seed vigour using static and dynamic traits, which can identify loci that regulate desired vigour-related features, stress tolerance and seed quality; (3) crop breeding for seed vigour, which enables breeders to incorporate favourable alleles into new crop varieties through marker-assisted

selection (MAS) and genomic selection in climate-resilient breeding programmes [52], ensuring better crop establishment, productivity, and resilience to climate change.

Materials and Methods

Plant materials and seed germination experiments

To assess seed vigour in wheat with phenotypic variations, we selected 21 commercial wheat genotypes. These varieties were chosen to capture a broad range of developmental paces during germination (**Fig. 1d**). Seeds were produced in pre-breeding field trials conducted in Cambridgeshire UK (52°23'85"N, 9°60'99"E) and were used within 6 months of harvest. Before the experiments, seed lots were stored at 10°C and 10-15% relative humidity to maintain viability. To evaluate germination speed, some seed lots were treated with cold stratification at 5°C for 24-72 hours in sealed plastic bags (**Table S1**).

Time-lapse imaging with different SeedGerm settings

Germination experiments were carried out in 2022 and 2023 in the UK. Two types of low-cost SeedGerm devices were built for time-lapse imaging: (1) a small translucent plastic germination box (dimensions: 31 × 39 × 48 cm) mounted with fixed second-hand Android smartphones (equipped with Sony IMX278 or IMX319 image sensors; a maximum of 3,840×2,160 pixels per image, roughly 5,000 pixels per seed), and (2) a big germination box (dimensions: 31 × 44 × 71cm) mounted with two types of *Raspberry Pi* cameras (i.e. Sony IMX477 or IMX219, with a maximum of 3,280×2,464 pixels per image; roughly 5,500 pixels per seeds). Different RGB sensors acquired HD images with different resolutions for a flexible hardware design (see image sensors in **Table S1**). Germination experiments were carried out on blue (Bärenstein, Germany) and black (Crown Supplies, UK) seed testing paper

under an ambient temperature of 20-22 °C, for 4-7 days depending on the duration of the cold treatments (i.e. 24, 48 and 72 hours). Time-lapse imaging was conducted at regular intervals of one hour, covering from imbibition (**Fig. 1d**, left) to seedling emergence, after chloroplast biogenesis when at least 10 mm green seedlings were visible (**Fig. 1d**, right).

Data annotation and augmentation

LabelMe [41] was used for data annotation. For the phase detection task of each seed during germination, a total of 48 images were labelled, covering the 4 germination phases, including 29 images of blue background and 19 images of black background. Due to the resolution differences and growth directions, augmentation methods (e.g. adding noise and flipping) were chosen to mimic imaging-related problems. After augmentation, a total of 151 images (which is called 'SeedVig-phase') were randomly divided into training, testing, and validation sets in a 7:2:1 ratio. For the mask prediction task, we labelled the seeds without roots and seedling, seeds with roots and seedling, and seedlings at early, middle, and late timepoints in the image series, respectively. A total of 32 images for seeds without roots and seedling, 36 for seeds with roots and seedlings, and 38 for seedlings were annotated. Due to the luminance change in different germination experiments, brightness adjustment was applied for image augmentation, and finally 96, 108 and 114 images were generated for seeds without roots and seedling, seeds with roots and seedling, and seedlings, respectively. The images (which is called 'SeedVig-traits') were divided randomly into 70% (for training) and 30% (for testing).

DL model selection and evaluation metrics

To detect seed objects using DL-powered models efficiently and accurately, we trained four DL models

using the ‘SeedVig-phase’ dataset with roots and seedlings, including U-Net [40], PSP-Net [53], HR-Net [54], and DeepLabV3+ [55]. When testing these DL models, every model was trained for 300 epochs and their performance was evaluated through metrics such as mean intersection over union (mIoU), mean pixel accuracy (mPA), and Accuracy. Through the evaluation, the U-Net model was selected as the architecture to train a DL model for seed objects segmentation (**Table 1**).

Table 2. Performance analysis of different DL models used for seed detection.

Model	mIoU (%)	mPA (%)	~~Precision~~Accuracy (%)
U-~~net~~Net	88.96	92.99	99.10
PSP-~~net~~Net	82.30	88.00	98.34
HR-Net	83.87	89.60	98.49
DeepLabV3+	80.26	88.07	98.33

Formatted Table

Similarly, we trained four DL models using the ‘SeedVig-traits’ dataset to segment seed objects accurately, including SSD [56], Faster-RCNN [57], YOLOv8 [39], and RE-DETR [58]. When testing these DL models, every model was trained for 100 epochs and their performance was evaluated through metrics such as Precision, Recall, mAP50 (mean average precision at IoU =0.5), and F1 score. Through the evaluation, the YOLOv8 model was selected as the architecture for seed detection (**Table 2**).

Table 3. Performance analysis of different DL models trained for identifying germination phases.

Model	Precision (%)	Recall (%)	mAP50 (%)	F1 (%)
YOLOv8	88.10	94.30	95.30	91.11
SSD	82.20	56.75	67.70	61.75

Faster-RCNN	63.38	41.98	52.14	50.25
RT-DETR	72.10	79.30	76.60	75.10

The SeedGerm-VIG pipeline for identifying germination phases

Images of blue germination paper were cropped according to ROIs. For images of black germination paper, rotation or translation were applied to correct slight camera shifts at the start of the image series. After establishing the training set, the standard YOLOv8x model [39] was trained for 2,000 epochs, which was used to identify seed-level germination phases in the experiment. A seed lots' germination phase was defined when 75% seeds of the seed lot entered a specific phase (**Fig. 2b**).

The SeedGerm-VIG pipeline for seed-level trait analysis

After establishing the training set, we followed the a previously reported approach of [40] and used the pre-trained VGG-16 [59] architecture as the backbone (encoding part) of the standard U-Net model [60]. These is three U-Net models was were trained for 1,000, 400, and 400 epochs to predict masks of seed coat, seed/roots, and seedling of each seed, respectively. The seed morphologies, including seed area, seed length, seed width, seed perimeter, seed W/L ratio, and seed roundness, were quantified through a previous described method [32], while seed colours were quantified by the mean values of R, G, and B channels in the RGB colour space of the seed objects (**Table S11S13**). The change rates of morphological features were quantified based on the obtained static phenotypes (i.e. values at different timepoints).

Using a root tip tracking algorithm, the root tips' positional changes were recorded as the corresponding root change rates and cumulative root lengths were quantified by reconstructing the lines

based on the positional changes of root tips across image series (**Fig. S6**). Seedling emergence was recorded when the seedling mask first overlapped with the mask of seeds without roots and seedling within a ROI (i.e., bounding box). For example, the regions corresponding to seedling mask were converted to excess green (ExG) values [61]; and, the chloroplast biogenesis threshold (greenness value) was computed based on the mean of ExG values [62]. If the ExG value of any pixel in the seedling mask exceeded the threshold, the timepoint was recorded as chloroplast biogenesis. The duration from coleoptile emergence to chloroplast biogenesis was quantified based on the timepoints.

Graph-based root emergence tracking at the seed level

Based on the ROIs obtained, the predicted masks of seeds without roots and seedling and seeds with roots and seedling were used for root tip tracking. After extracting the skeletons (**Fig. 3a**), the root tips were determined by removing skeletons overlapped with seed coat and seedling. Then the root tips were numbered according to their emergence order, with their positional changes of root tips quantified based on their coordinates. As germination progresses, roots often intersect, complicating tracking efforts. To address this issue, we developed algorithms to segment 3-vertice and 4-vertice cliques [63]. After extracting skeletons (**Fig. S7**), branch point (red) or equivalent branch point (i.e. the midpoint of two branch points; blue) were identified, alongside root tips (green) within the local region. Vectors were then constructed from these (equivalent) branch point to the root tips, so that the intersected skeletons could be segmented based on the vector angle, with preference given to angle closer to 180°. Finally, the 2D skeletons were assembled for later analysis.

Building on root tip coordinates and intersection segmentation algorithm, this study utilized temporal graph [64] to track radicle tips. Although the SmartRoot toolkit [65] was reported to use a 90° scanning

range to search for intersection roots from a side perspective, the 60° scanning setting used in this study for identifying intersection roots from overhead images were proved more useful in tracking root tips in complex root emergence images.

Using seed 2 of G7 (Row 1 Column 2) as an exemplar (**Fig. 3a**), each root was treated as a temporal graph where the initial vertex V_0 and edge E_0 were both ∅. The primary root (radicle) emerged at the 40th hour (t_e), where $V_{t_e\_s}$ denoted the skeleton point connecting to the seed coat and the root tip was taken as V_t, with the skeleton connecting the two nodes defined as E_t. To continuously track root tips of different root, routes connecting C_t (current root tips) to V_{t-1} were taken into consideration, along with their angles. For instance, at timepoint $t+1$, iterating the root tips in C_t, when root tip P met two requirements: (1) the route connecting P to V_t had the minimum weight (where skeleton points weighed 0 and others weighed 1); (2) the angle between $\overrightarrow{V_{t-1}\ V_t}$ and $\overrightarrow{V_t\ P}$ was no more than 60°, the G_{t+1} was updated accordingly. Through this process, the radicle growth process could be tracked and quantified (**Fig. 3a**). To avoid tracking errors, after radicle intersections, newly emerged radicles were not tracked. Formulas used to perform the tracking are listed below:

$$G_t = \begin{cases} (V_0, E_0), & \text{if } t < t_e - 1 \\ (V_{t_e\_s}, E_0), & \text{if } t = t_e - 1 \\ (V_t, E_t), & \text{if } t > t_e - 1 \end{cases}$$

$$\begin{cases} V_{t+1} = V_t \cup \{P\} \\ E_{t+1} = E_t \cup L(V_t, P) \end{cases}$$

Where at timepoint $t+1$, P should meet $P \in C_{t+1}$, when $\|V_t - P\| = \min_{P_l \in C_{t+1}} \| V_t - P_l\|$ and $cos\left(\overrightarrow{V_{t-1}\ V_t}, \overrightarrow{V_t\ P}\right) \geq 0.5$.

Statistical analysis and manual scoring

After trait ~~extraction~~analysis, the scales provided in Table S1 were used to convert the pixel units into millimetres (mm). To verify the performance of the SeedGerm-VIG pipeline, a total of 84 images (4 images per genotype), covering the early, middle, and late stages of seed germination experiments, were randomly selected for manual scoring. During the IMB phase, seed length, seed width, seed perimeter, and seed area were measured manually using ImageJ [66]. In the subsequent phases (including PRO, SE, and RE), the lengths of roots and seedlings were assessed manually. Similarly, a total of 18 images of rice and barley were selected for manual measurement of root lengths. To verify the seed vigour of different wheat genotypes, the seed germination images at the 80th hour were used for assessment. Clustering analysis was performed using the 'Scikit-learn' library [67], while correlation analysis utilized the Pearson correlation coefficient and *p*-value after removing the outliers.

To our knowledge, we could not find any existing toolkits that could be used to quantify the emergence of radicles and seedlings from overhead imagery over time. Hence, we chose two representative research tools such as *SeedExtractor* [29] and *SeedGerm* [32] to evaluate seed-level morphological and colour traits between the SeedGerm-VIG pipeline and the two software packages. The correlation analysis indicated a strong correlation (correlation coefficient, $r > 0.79$) for the morphological traits (e.g. seed length, width, and area) measured between the SeedGerm-VIG and *SeedGerm*, as well as a significant correlation ($r > 0.91$) for RGB colour features measured by the SeedGerm-VIG and *SeedExtractor* (**Table S13**). The above suggested that the SeedGerm-VIG pipeline was able to provide reliable analyses of seed size and colour features compatible with results produced by methods previously reported.

Software implementation

When training the YOlOv8x-Germ and U-Net models, a Windows 10 workstation (16 GB memory, Nvidia GTX 1660Ti GPU, and Intel Core i7-10700F CPU) was used, along with TensorFlow (V2.2) framework [68] and Python (V3.7) for the model implementation. We applied key open scientific development libraries in this study including the scientific data processing library SciPy [69] and the image processing library Scikit-Image [70]. All figures, except raincloud plots, were plotted using the Python libraries 'matplotlib' and 'seaborn' [71,72]. Line plots with confidence intervals were created by a parametric method (i.e. confidence intervals = 75%) using the 'seaborn.relplot' function with parameters 'kind="line", errorbar=("ci", 75)'. Raincloud plots were generated using the R packages 'ggplot2' and 'ggdist' [73,74]. To facilitate a broader community to access our work, source code and DL models of the SeedGerm-VIG together with executable Jupyter notebooks [75]. When developing the GUI of the pipeline, widget enabled by Jupyter notebook was used together with testing data to assist nonexpert users to execute the SeedGerm-VIG pipeline (**Fig. S14**; **Open Access**).

Abbreviations

Barley genotype (BG), computer vision (CV), deep learning (DL), excess green (ExG), germination index (GI), germination potential (GP), growth stage (GS), graphic user interface (GUI), high definition (HD), imbibition (IMB), International Seed Testing Association (ISTA), machine learning (ML), marker-assisted selection (MAS), mean germination time (MGT), protrusion (PRO), radicle emergence (RE), red-green-blue (RGB), region of interest (ROI), rice genotype (RG), root 1 (R1; i.e. radicle), root 2 (R2), root 3 (R3), seedling establishment (SE), time of 50% cumulative germination (T_{50}), wheat genotypes (WG), width and length ratio (W/L).

Open Access

Source code, trained learning models, and ~~algorithm~~algorithms of SeedGerm-VIG are distributed under the MIT license (Creative Commons Attribution 4.0 international license~~,~~), permitting academic use, distribution, and reproduction in any medium~~, provided you give appropriate credit to the original authors and the source, provide a link to the Creative Commons license, and indicate if changes were made~~. Unless otherwise stated, the Creative Commons Public Domain Dedication (http://creativecommons.org/licenses/by/4.0) waiver applies to the data and results made available here. ~~Source code, the~~ The SeedVig-phase and SeedVig-traits ~~set~~training sets, seed germination image series (i.e. wheat, barley, and ~~other test~~rice), are under the CC0 public waiver and are available to the BioImage Archive repository S-BIAD1852 (DOI: 10.6019/S-BIAD1852). Test data supporting the results in the article are available at https://Github.com/The-Zhou-Lab/SeedGerm-VIG/releases. Other source code, data and user guides are openly available on request.

Author contributions

Ji Zhou, Jie Dai and ~~Mujiahid~~Mujahid Ali wrote the manuscript with inputs from all the authors; Zhenjie Wen, Felipe Pinheiro, and Jie Dai conducted experiments under Changcai Yang, Xueying Guan, Lingzhen Ye and Ji Zhou's supervision; Jie Dai, Shuchen Liu, Jinlong Huang, Zhenjie Wen, and Bing Wang built the datasets for deep learning models under Ji Zhou's supervision; Jinlong Huang and Jie Dai, Zhenjie Wen, and Shuchen Liu measured the manual data and performed statical analysis and result interpretation under Ji Zhou's supervision; Jie Dai, Jianhua Zhao, and Ji Zhou developed the SeedGerm-VIG pipeline; ~~Mujiahid~~Mujahid Ali, Zhenjie Wen and Xueying Guan helped revise the manuscript. All

authors read and approved the manuscript. Jie Dai, Zhenjie Wen, and ~~Mujiahid~~Mujahid Ali contributed equally to this work.

Funding

This work and the Zhou lab members at NAU were supported by the National Natural Science Foundation of China (32070400 & U24A20402). Ji Zhou and Felipe Pinheiro were partially supported by the Allan & Gill Gray Foundation' Sustainable Productivity for Crop Improvement (G118688 to the University of Cambridge and NIAB). Ji Zhou was supported by the United Kingdom Research and Innovation's (UKRI) Biotechnology and Biological Sciences Research Council (BBSRC) AI in Bioscience Grant (BB/Y513969/1 to Ji Zhou). The UK-China research activities were supported by the BBSRC's International Partnership Grant (BB/Y514081/1 to NIAB). AI model training was partially conducted on the CropDiversity HPC funded by the BBSRC's ALERT grant (BB/X019683/1 to James Hutton Institute).

Acknowledgements

The authors would like to thank all members of the Zhou laboratory at the Nanjing Agricultural University (NAU) China and Cambridge Crop Research, the National Institute of Agricultural Botany (NIAB) UK for fruitful discussions. In particular, the authors would like to thank Jimmy Zhang at Imperial College for his supports in data collection and low-cost SeedGerm hardware development, Jie Zhou, Gang Sun, and Liyan Shen at the NAU for their help improve deep learning modelling.

Competing interests

The authors declare no competing financial interests.

Tables

Table 1. The summary of traits quantified at three germination phases.

Table 2. Performance analysis of different DL models used for seed detection.

Table 3. Performance analysis of different DL models trained for identifying germination phases.

Figures

Figure 1. Time-lapse seed germination imaging and acquired image series using SeedGerm devices.

(**a**) A germinated wheat seed consisting of coleoptile, seed and radicles. (**b**) A set of low-cost SeedGerm devices equipped with second-hand smartphone or *Raspberry Pi* sensors to perform overhead time-lapse imaging. (**c**) Germination experiments with blue and black germination papers. (**d**) Representative seed- and seed-lot level images of genotype 4 (G4) collected at key germination phases during the germination procedure. Scale bars provided to show the size of seed, radicles, and seedling.

Figure 2. The analysis workflow of SeedGerm-VIG for identifying key germination phases for every seed in a seed lot, followed by the measurement of seed-level static and dynamic germination traits.

(**a**) A general analysis workflow for seed-level germination phase identification using the YOLOv8x-Germ model, with seed-level confidence displayed. (**b**) Seed-level germination phases identified by the YOLOv8x-Germ model from seed-lot level image series (upper: G2; bottom: G7) together with bounding boxes, ranging from imbibition (IMB), protrusion (PRO), radicle emergence (RE), to seedling

establishment (SE). For a given experiment, the germination phase (i.e. IMB, PRO, RE, or SE) was defined when over 75% of the seeds reached a certain phase. (**c**) Seed-level masks generated using a standard U-~~net~~Net model, dividing foreground objects (e.g. seeds, roots, and seedlings) from background signals (e.g. germination papers). (**d**) Phenotypic analysis of seeds, radicles and seedlings based on the identified foreground objects.

Figure 3. Tracking radicle and lateral root tips, growth speed profiles, and uniformity analysis during key germination phases.

(**a**) Radicle emergence of a seed (genotype 7, G7) measured using the root tip tracking algorithm with 5-hour intervals. (**b**) Seed-level radicle tracking results of G7 at the 80th hour, with root skeletons created and pseudo-coloured according to time (0-100 hours). (**c**) Profile curves of radicle length (left) and radicle growth rate (right) between 0 and 80 hours, which were classified into the Quick, Medium, and Slow Speed groups. (**d**) Raincloud plots used to demonstrate how uniform radicles in the three Speed groups reached 2 and 10 mm over time. (**d**) Raincloud plots show how uniform seeds' reached chloroplast biogenesis in the three Speed groups between 0 and 80 hours. Arrows point out seeds did not reach key germination phases. Uniformity scores were provided based on 75% of the data obtained from the experiments.

Figure 4. A comprehensive matrix provides an overview of 21 wheat genotypes' germination speed groups (pseudo-coloured) and uniformity scoring (with two decimal points) at three key phases, followed by the computation of overall seed vigour scores based on measures of phase-based speed and uniformity for the 21 genotypes.

Figure 5. The application of the SeedGerm-VIG pipeline to perform seed-level analyse of root (barley) and radicle (rice) emergence for 12 commercial barley and six rice genotypes.

(**a**) Barley seeds germination recorded on dark red germination papers and seed-level root emergence identified by the SeedGerm-VIG pipeline for a barley genotype (BG2) at the 90th hour, with seed-level bounding boxes showing seed germination phases (one seed still at protrusion was pointed with a white arrow) and root skeletons pseudo-coloured according to time (0-90 hours). (**b**) Root emergence curves produced to demonstrate germination speed and profiles for twelve barley commercial lines (15 seeds sampled per line) over 93 hours. (**c**) Rice seeds germination recorded on light blue germination papers and seed-level radicle emergence identified by the SeedGerm-VIG pipeline for a rice genotype (RG3) at the 85th hour, with seed-level bounding boxes showing germination phases (one seed was still at imbibition and one seed was at protrusion) and radicle skeletons pseudo-coloured according to time (0-85 hours). (**d**) Radicel emergence curves produced to show germination speed and profiles for six rice commercial lines (15 seeds sampled per line) over 85 hours.

Supplementary Material

Table S1. Experimental settings for 21 commercial wheat varieties for germination.

Table S2. Descriptive statistics and broad-sense heritability of ~~time points~~timepoints when radicles reached

2 mm.

Table S3. Descriptive statistics and broad-sense heritability of ~~time points~~timepoints when radicles reached 10 mm.

Table S4. Descriptive statistics and broad-sense heritability of ~~time points~~timepoints when chloroplast biogenesis was detected.

Table S5. Three clusters of 21 wheat genotypes using the agglomerative clustering algorithm based on radicle growth rates, from 2 mm to 10 mm.

Table S6. Three clusters of 21 genotypes using the affinity propagation algorithm based on ~~time points~~timepoints of chloroplast biogenesis.

Table S7. Three clusters of 21 genotypes using the K-means algorithm based on ~~time points~~timepoints of key germination phases at the seed-lot level.

Table S8. Three clusters of 21 genotypes using the affinity propagation algorithm based on timepoints of radicle emergence and seedling emergence at the seed-lot level.

Table S9. Manual assessments of the 21 wheat genotypes for germination speed.

Table S10. Coefficient of variation of the 75% data from the three germination speed groups based on ~~time points~~timepoints of key germination phases and chloroplast biogenesis.

Table S11. Interquartile range of the data from the three germination speed groups based on timepoints of key germination phases and chloroplast biogenesis.

Table S12. Three clusters of 12 barley genotypes using the affinity propagation algorithm based on radicle growth rates, from 2 mm to 10 mm.

Table ~~S12~~S13. Correlation (*R*) of the three automated analytic approaches when measuring different seed parameters.

Note S1. Algorithmic steps to correct seed positions and ~~rot~~root tips tracking.

Note S2. Exceptional cases that the SeedGerm-VIG made mistakes.

Figure S1. A high-quality seed germination training set for deep learning modelling.

Figure S2. Confusion matrixes for evaluating the accuracy of identifying key germination phases.

Figure S3. Profile curves of wheat genotypes reaching a germination phase (15 seeds sampled from every genotype).

Figure S4. Correlation analysis between manual and computational measures of seed, root, and seedling traits across 21 wheat genotypes.

Figure S5. Correlation analysis between traditional and SeedGerm-VIG derived traits across 21 wheat genotypes.

Figure S6. Positional changes (30-65 hours) of root tips for a given seed (Row 3, Column 1) from the G4 seed lot.

Figure S7. The algorithmic steps to identify roots from images with intersected roots.

Figure S8. Profile curves of radicle growth for 21 wheat genotypes during germination.

Figure S9. Profile curves of first three roots for 21 wheat genotypes.

Figure S10. Raincloud plots of seed area and seed width changes during the imbibition (IMB) phase for three germination speed groups.

Figure S11. Root analysis of the first three roots in the Quick, Medium, and Slow germination speed groups.

Figure S12. Root-based germination analysis for twelve barley genotypes.

Figure S13. Radicle emergence analysis for six rice genotypes.

Figure S14. The graphic user interfaces (GUIs) developed using widget in Jupyter notebooks to automate tasks in the SeedGerm-VIG pipeline.

References

1. Seppelt R, Klotz S, Peiter E, et al. Agriculture and food security under a changing climate: An underestimated challenge. *iScience*. 2022; 25(12):105551. ~~https://doi.org/~~10.1016/j.isci.2022.105551.

2. Hawkesford MJ, Araus JL, Park R, et al~~.~~.. Prospects of doubling global wheat yields. *Food Energy Secur*. 2013; 2(1):34-48. ~~https://doi.org/~~10.1002/fes3.15.

3. Li S, Tian Y, Wu K, et al~~.~~.. Modulating plant growth–metabolism coordination for sustainable agriculture. *Nature*. 2018; 560:595-600. ~~https://doi.org/~~10.1038/s41586-018-0415-5.

4. Gang A, Vyas A VH. Toxic effect of heavy metals on germination and seedling growth of wheat. *J Environ Res Dev*. 2013;8:206–13.

~~4~~5. Hojat Salehzade, Mousa Izadkhah Shishvan, Mehdi Ghiyasi, et al. Effect of Seed Priming on Germination and Seedling Growth of Wheat (*Triticum aestivum* L.). *Res J Biol Sci*. 2009;4(5):629–31.

~~5. Gang A, Vyas A VH. Toxic effect of heavy metals on germination and seedling growth of wheat. *J Environ Res Dev*. 2013;8:206–13.~~

6. Mwendwa JM, Brown WB, Weidenhamer JD, et al~~.~~.. Evaluation of commercial wheat cultivars for canopy architecture, early vigour, weed suppression, and yield. *Agronomy*. 2020; 10(7):983. ~~https://doi.org/~~10.3390/agronomy10070983.

7. Finch-Savage WE, Bassel GW. Seed vigour and crop establishment: Extending performance beyond adaptation. *J Exp Bot*. 2016; 67(3):567-91. ~~https://doi.org/~~10.1093/jxb/erv490.

8. S. Ambika ~~S,~~, V. Manonmani ~~V, Somasundaram G~~GS. Review on effect of seed size on seedling vigour and seed yield. *Res J Seed Sci*. 2014;7(2):31–8.

9. ISTA. Seed Vigour Testing. *Int Rules Seed Test*. In: The International Seed Testing Association

(ISTA). Switzerland: Bassersdorf; 2021. p. i-15-20(20).

10. McDonald MB. Seed quality assessment. *Seed Sci Res*. 1998; 8(2):265-76. ~~https://doi.org/~~10.1017/s0960258500004165.

11. Powell AA. Seed vigour in the ~~21st~~21 st century. *Seed Sci Technol*. 2022; 50:45-73. ~~https://doi.org/~~10.15258/sst.2022.50.1.s.04.

12. Bettey M, Finch-Savage WE, King GJ, et al. Quantitative genetic analysis of seed vigour and pre-emergence seedling growth traits in Brassica oleracea. *New Phytol*. 2000; 148(2):277-86. ~~https://doi.org/~~10.1046/j.1469-8137.2000.00760.x.

13. Penfield S, King J. Towards a systems biology approach to understanding seed dormancy and germination. *Proc R Soc B Biol Sci*. 2009; 276(1673):3561-69. ~~https://doi.org/~~10.1098/rspb.2009.0592.

14. Marcos-Filho J. Seed vigor testing: An overview of the past, present and future perspective. *Sci Agric*. 2015; 72(4):363-374. ~~https://doi.org/~~10.1590/0103-9016-2015-0007.

15. Khajeh-Hosseini M, Gallo C, Wagner M H, et al. Proposal for the addition of wheat (*Triticum aestivum*) as a species to which the radicle emergence test for seed vigour can be applied. *Seed Test Int*. 2018;157:40–1.

16. Matera TC, Pereira LC, Braccini AL, ~~Krzyzanowski FC, Scapim CA, Piana SC, et al.~~.et al. Accelerated aging test and its relationship to physiological potential of soybean seeds. *J Seed Sci*. 2019; 41(3):301-8. ~~https://doi.org/~~10.1590/2317-1545v41n3212746.

17. Matthews S, Powell A. Electrical Conductivity Vigour Test: Physiological Basis and Use. *Seed Test Int*. 2006;131:32–5.

18. Demir I, Ermis S, Okçu G, et al. Vigour tests for predicting seedling emergence of aubergine

Formatted: Font: Not Italic

(*Solanum melongena* L.) seed lots. *Seed Sci Technol*. 2005;33(2):484-4. ~~https://doi.org/~~10.15258/sst.2005.33.2.20.

19. Santos JF, Dirk LMA, Bruce Downie A, et al. Reciprocal effect of parental lines on the physiological potential and seed composition of corn hybrid seeds. *Seed Sci Res*. 2017;27(3):206-16. ~~https://doi.org/~~10.1017/S0960258517000095.

20. Wen D, Hou H, Meng A, et al. Rapid evaluation of seed vigor by the absolute content of protein in seed within the same crop. *Sci Rep*. 2018;8:5569. ~~https://doi.org/~~10.1038/s41598-018-23909-y.

21. Liu W, Liu J, Jiang J, et al. Comparison of partial least squares-discriminant analysis, support vector machines and deep neural networks for spectrometric classification of seed vigour in a broad range of tree species. *J Near Infrared Spectrosc*. 2021;29(1):33-41. ~~https://doi.org/~~10.1177/0967033520963759.

22. Al-Amery M, Geneve RL, Sanches MF, et al. Near-infrared spectroscopy used to predict soybean seed germination and vigour. *Seed Sci Res*. 2018;28(3):245-52. ~~https://doi.org/~~10.1017/S0960258518000119.

23. Umarani R, Bhaskaran M, Vanitha C, et al. Fingerprinting of volatile organic compounds for quick assessment of vigour status of seeds. *Seed Sci Res*. 2020;30(2):112-21. ~~https://doi.org/~~10.1017/S0960258520000252.

24. Snider JL, Collins GD, Whitaker J, et al. The impact of seed size and chemical composition on seedling vigor, yield, and fiber quality of cotton in five production environments. *F Crop Res*. 2016;193:186-95. ~~https://doi.org/~~10.1016/j.fcr.2016.05.002.

25. Mandizvo T, Odindo AO. Seed coat structural and imbibitional characteristics of dark and light coloured Bambara groundnut (*Vigna subterranea* L.) landraces. *Heliyon*. 2019;5(2):e01249.

Formatted: Font: Not Italic

~~https://doi.org/~~10.1016/j.heliyon.2019.e01249.

26. Reed RC, Bradford KJ, Khanday I. Seed germination and vigor: ensuring crop sustainability in a changing climate. *Heredity*. 2022;128:450-9. ~~https://doi.org/~~10.1038/s41437-022-00497-2.

27. Wani BA, Ram M, Yasin BA, et al. Seedling vigour in wheat (*Triticum aestivum* L.) as a source of genetic variation and study of its correlation with yield and yield components. *African J Agric Res*. 2013;8(4):370-2. ~~https://doi.org/~~10.5897/ajar12.1375.

28. Jahnke S, Roussel J, Hombach T, et al. *pheno*Seeder - A Robot System for Automated Handling and Phenotyping of Individual Seeds. *Plant Physiol*. 2016;172(3):1358-70. ~~https://doi.org/~~10.1104/pp.16.01122.

29. Zhu F, Paul P, Hussain W, et al. *SeedExtractor*: An Open-Source GUI for Seed Image Analysis. *Front Plant Sci*. 2021;11:581546. ~~https://doi.org/~~10.3389/fpls.2020.581546.

30. Joosen RVLL, Kodde J, Willems LAJJ, et al. Germinator: A software package for high-throughput scoring and curve fitting of Arabidopsis seed germination. *Plant J*. 2010;62(1):148-59. ~~https://doi.org/~~10.1111/j.1365-313X.2009.04116.x.

31. Braguy J, Ramazanova M, Giancola S, et al. SeedQuant: a deep learning-based tool for assessing stimulant and inhibitor activity on root parasitic seeds. ~~*Plant Physiol.*~~ 2021;186(3):1632-44. ~~https://doi.org/~~10.1093/plphys/kiab173.

32. Colmer J, O'Neill CM, Wells R, et al. SeedGerm: a cost-effective phenotyping platform for automated seed imaging and machine-learning based phenotypic analysis of crop seed germination. *New Phytol*. 2020;228(2):778-93. ~~https://doi.org/~~10.1111/nph.16736.

33. Yasrab R, Atkinson JA, Wells DM, et al. RootNav 2.0: Deep learning for automatic navigation of complex plant root architectures. *Gigascience*. 2019;8(11):giz123.

~~https://doi.org/~~10.1093/gigascience/giz123.

34. da Silva Medeiros ML, Cruz-Tirado JP, Lima AF, et al. Assessment oil composition and species discrimination of *Brassicas* seeds based on hyperspectral imaging and portable near infrared (NIR) spectroscopy tools and chemometrics. *J Food Compos Anal*. 2022;107:104403. ~~https://doi.org/~~10.1016/j.jfca.2022.104403.

35. Shi T, Gao Y, Song J, et al. Using VIS-NIR hyperspectral imaging and deep learning for non-destructive high-throughput quantification and visualization of nutrients in wheat grains. *Food Chem*. 2024;461:140651. ~~https://doi.org/~~10.1016/j.foodchem.2024.140651.

36. Weitbrecht K, Müller K, Leubner-Metzger G. First off the mark: Early seed germination. *J Exp Bot*. 2011;62(10):3289-309. ~~https://doi.org/~~10.1093/jxb/err030.

37. Pogson BJ, Ganguly D, Albrecht-Borth V. Insights into chloroplast biogenesis and development. *Biochim Biophys Acta - Bioenerg*. 2015;1847(9):1017-24. ~~https://doi.org/~~10.1016/j.bbabio.2015.02.003.

38. Maqbool S, Hassan MA, Xia X, et al. Root system architecture in cereals: progress, challenges and perspective. *Plant J*. 2022;110(1):23-42. ~~https://doi.org/~~10.1111/tpj.15669.

39. Reis D, Kupec J, Hong J, et al. Real-Time Flying Object Detection with YOLOv8. *arXiv*. 2023. ~~https://doi.org/~~10.48550/arXiv.2305.09972.

40. Hasal M, Pecha M, Nowaková J, et al. Retinal Vessel Segmentation by U-Net with VGG-16 Backbone on Patched Images with Smooth Blending. In: Barolli L, editor. *Advances in Intelligent Networking and Collaborative Systems*. Switzerland: Springer; 2023. p. 465–74.

41. Russell BC, Torralba A, Murphy KP, et al. LabelMe: A database and web-based tool for image annotation. *Int J Comput Vis*. 2008;77:157-73. ~~https://doi.org/~~10.1007/s11263-007-0090-8.

42. Mangrich ME, Saltveit ME. Heat shocks reduce chilling sensitivity of cotton, kenaf, okra, and rice seedling radicles. *J Am Soc Hortic Sci*. 2000;125(3):377-82. ~~https://doi.org/~~10.21273/jashs.125.3.377.

43. Rahnama A, Fakhri S, Meskarbashee M. Root growth and architecture responses of bread wheat cultivars to salinity stress. *Agron J*. 2019;111(6):2991-8. ~~https://doi.org/~~10.2134/agronj2018.12.0795.

44. Jain AK, Murty MN, Flynn PJ. Data clustering: A review. *ACM Comput Surv*. 1999;31(3):264-323. ~~https://doi.org/~~10.1145/331499.331504.

45. Frey BJ, Dueck D. Clustering by passing messages between data points. *Science*. 2007;315(5814):972-6. ~~https://doi.org/~~10.1126/science.1136800.

46. Li W, He X, Chen Y, et al. A wheat transcription factor positively sets seed vigour by regulating the grain nitrate signal. *New Phytol*. 2020;225(4):1667-80. ~~https://doi.org/~~10.1111/nph.16234.

47. Ramappa S, Joshi MA, Krishna H, et al. Unravelling the ~~genetic basis~~Genetic Basis of ~~moisture-deficit stress tolerance~~Moisture Deficit Stress Tolerance in ~~wheat~~Wheat for ~~seedling vigour-related traits~~Seedling Vigour-Related Traits and ~~root traits using genome-wide association study.~~Root Traits Using Genome-Wide Association Study. *Genes*. 2023;14(10):1902. ~~https://doi.org/~~10.3390/genes14101902.

48. Arya S, Sandhu KS, Singh J, et al. Deep learning: as the new frontier in high-throughput plant phenotyping. *Euphytica*. 2022;218:47. ~~https://doi.org/~~10.1007/s10681-022-02992-3.

49. Demir I, Ermis S, Mavi K, et al. Mean germination time of pepper seed lots (*Capsicum annuum* L.) predicts size and uniformity of seedlings in germination tests and transplant modules. *Seed Sci Technol*. 2008;36(1):21-30. ~~https://doi.org/~~10.15258/sst.2008.36.1.02.

~~50.~~50. Krizhevsky A, Sutskever I, Hinton GE. ImageNet classification with deep convolutional neural networks. *Commun ACM*. 2017;60(6):84-90. 10.1145/3065386.

51. Gibbs JA, Pound M, French AP, et al. Plant phenotyping: An active vision cell for three-dimensional plant shoot reconstruction. *Plant Physiol*. 2018;178(2):524-534. 10.1104/PP.18.00664.

52. Moore C, Rebetzke G. Genomic regions for embryo size and early vigour in multiple wheat (*Triticum aestivum* L.) populations. *Agronomy*. 2015;5(2):152-79. ~~https://doi.org/~~10.3390/agronomy5020152.

~~51~~53. Zhao H, Shi J, Qi X, et al. Pyramid Scene Parsing Network. *2017 IEEE Conf Comput Vis Pattern Recognit*. 2017;6230-9. ~~https://doi.org/~~10.1109/CVPR.2017.660.

~~52~~54. Wang J, Sun K, Cheng T, et al. Deep High-Resolution Representation Learning for Visual Recognition. *IEEE Trans Pattern Anal Mach Intell*. 2021;43(10):3349-64. ~~https://doi.org/~~10.1109/TPAMI.2020.2983686.

~~53~~55. Chen LC, Zhu Y, Papandreou G, et al. Encoder-Decoder with Atrous Separable Convolution for Semantic Image Segmentation. In: Ferrari V, Hebert M, Sminchisescu C, Weiss Y, editors. *Comput Vis -- ECCV 2018*. Cham: Springer; 2018. p. 833–51.

~~54~~56. Liu W, Anguelov D, Erhan D, et al. SSD: Single Shot MultiBox Detector. In: Leibe B, Matas J, Sebe N, Welling M, editors. *Comput Vis -- ECCV 2016*. Cham: Springer; 2016. p. 21–37.

~~55~~57. Ren S, He K, Girshick R, et al. Faster R-CNN: Towards Real-Time Object Detection with Region Proposal Networks. *IEEE Trans Pattern Anal Mach Intell*. 2016;39(6):1137-49. ~~https://doi.org/~~10.1109/TPAMI.2016.2577031.

~~56~~58. Zhao Y, Lv W, Xu S, et al. DETRs Beat YOLOs on Real-time Object Detection. *2024 IEEE/CVF Conf Comput Vis Pattern Recognit*. 2024;16965-74. ~~https://doi.org/~~10.1109/CVPR52733.2024.01605.

~~57~~59. Simonyan K, Zisserman A. Very deep convolutional networks for large-scale image

recognition. *3rd Int Conf Learn Represent ICLR 2015 - Conf Track Proc*. 2015;1-14. https://doi.org/10.48550/arXiv.1409.1556.

~~58~~60. Ronneberger O, Fischer P, Brox T. U-Net: Convolutional Networks for Biomedical Image Segmentation. In: Navab N, Hornegger J, Wells WM, Frangi AF, editors. *Medical Image Computing and Computer-Assisted Intervention – MICCAI 2015*. Cham: Springer; 2015. p. 234–41.

~~59~~61. Woebbecke DM, Meyer GE, Von Bargen K, et al. Color indices for weed identification under various soil, residue, and lighting conditions. *Trans Am Soc Agric Eng*. 1995;38(1):259-69. ~~https://doi.org/~~10.13031/2013.27838.

~~60~~62. Ridler TW, Calvard S. Picture thresholding using an iterative selection method. *IEEE Trans Syst Man Cybern*. 1978;8(8):630-2. ~~https://doi.org/~~10.1109/TSMC.1978.4310039.

~~61~~63. De J, Li H, Cheng L. Tracing retinal vessel trees by transductive inference. *BMC Bioinformatics*. 2014;15:20. ~~https://doi.org/~~10.1186/1471-2105-15-20.

~~62~~64. Michail O. An Introduction to Temporal Graphs: An Algorithmic Perspective. In: Zaroliagis C, Pantziou G, Kontogiannis S, editors. *Algorithms, Probability, Networks, and Games: Scientific Papers and Essays Dedicated to Paul G. Spirakis on the Occasion of His 60th Birthday*. Cham: Springer; 2015. p. 308-43.

~~63~~65. Lobet G, Pagès L, Draye X. A novel image-analysis toolbox enabling quantitative analysis of root system architecture. *Plant Physiol*. 2011;157(1):29-39. ~~https://doi.org/~~10.1104/pp.111.179895.

~~64~~66. Schneider CA, Rasband WS, Eliceiri KW. NIH Image to ImageJ: 25 years of image analysis. *Nat Methods*. 2012;9:671-5. ~~https://doi.org/~~10.1038/nmeth.2089.

~~65~~67. Pedregosa F, Varoquaux G, Gramfort A, et al. Scikit-learn: Machine Learning in Python. *J Mach Learn Res*. 2011;12:2825–30.

~~66~~68. Rampasek L, Goldenberg A. TensorFlow: Biology's Gateway to Deep Learning? *Cell Syst*. 2016;2(1):12-4. https://doi.org/10.1016/j.cels.2016.01.009.

~~67~~69. Virtanen P, Gommers R, Oliphant TE, et al. SciPy 1.0: fundamental algorithms for scientific computing in Python. *Nat Methods*. 2020;17:261-72. ~~https://doi.org/~~10.1038/s41592-019-0686-2.

~~68~~70. Van Der Walt S, Schönberger JL, Nunez-Iglesias J, et al. Scikit-image: Image processing in python. *PeerJ*. 2014;2:e453. ~~https://doi.org/~~10.7717/peerj.453.

~~69~~71. Waskom M. Seaborn: Statistical Data Visualization. *J Open Source Softw*. 2021;6(60):3021. ~~https://doi.org/~~10.21105/joss.03021.

~~70~~72. Hunter JD. ~~Matplotlib~~MATPLOTLIB: A 2D ~~Graphics Environment.~~GRAPHICS ENVIRONMENT. *Comput Sci Eng*. 2007;9(3):90–5. ~~https://doi.org/~~10.1109/MCSE.2007.55.

~~71~~73. Wickham H. ggplot2: Elegant Graphics for Data Analysis. 1st ed. Springer: New York; 2009.

~~72~~74. Kay M. ggdist: Visualizations of Distributions and Uncertainty in the Grammar of Graphics. *IEEE Trans Vis Comput Graph*. IEEE; 2024;30(1):414-24. ~~https://doi.org/~~10.1109/TVCG.2023.3327195.

~~73~~75. Randles BM, Pasquetto IV, Golshan MS, et al. Using the Jupyter Notebook as a Tool for Open Science: An Empirical Study. *Proc ACM/IEEE Jt Conf Digit Libr*. 2017;1-2. ~~https://doi.org/~~10.1109/JCDL.2017.7991618.

Figure 1

Click here to access/download;Figure;Slide1.jpeg

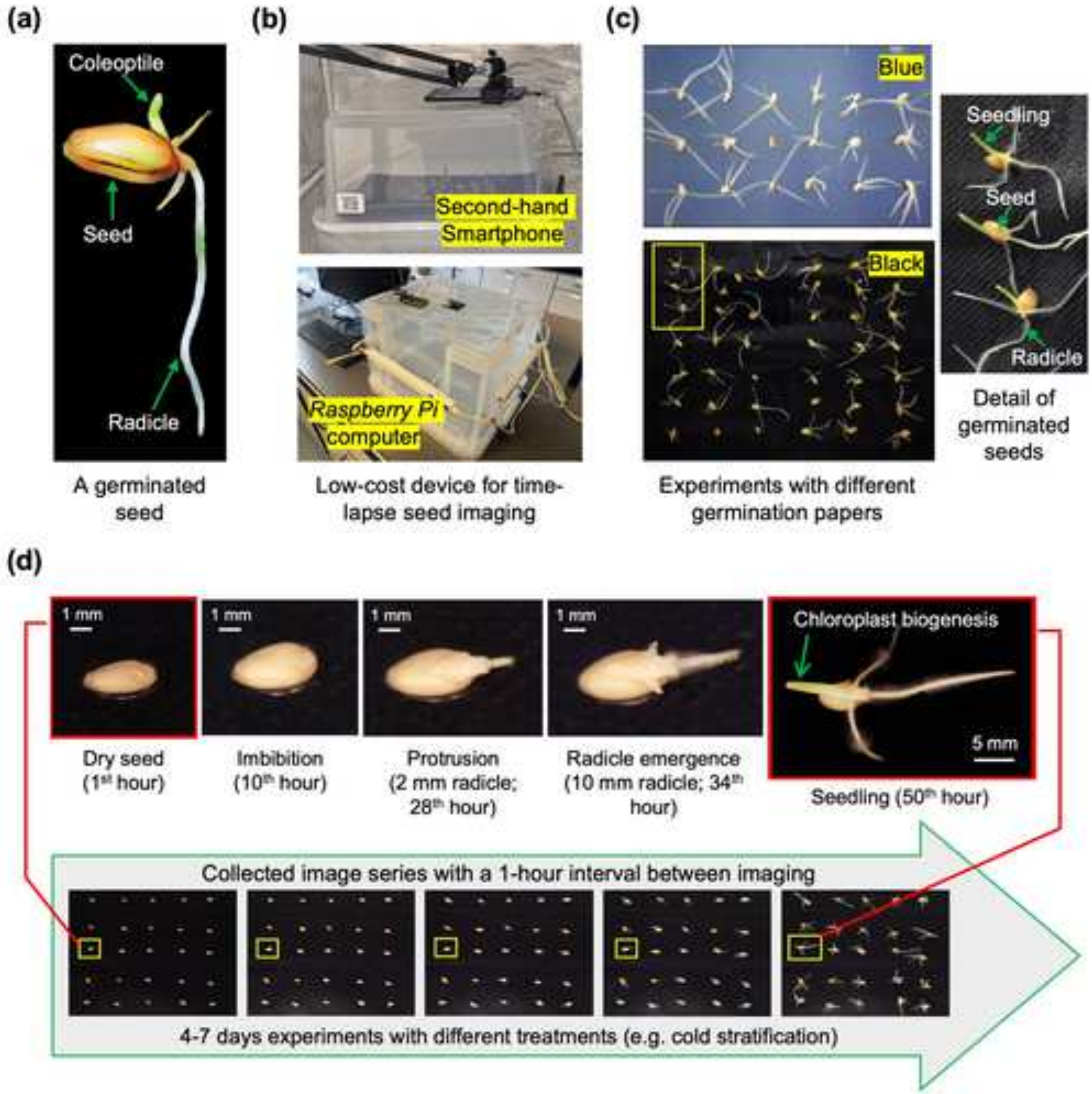

Figure 2

Click here to access/download;Figure;Slide2.jpeg

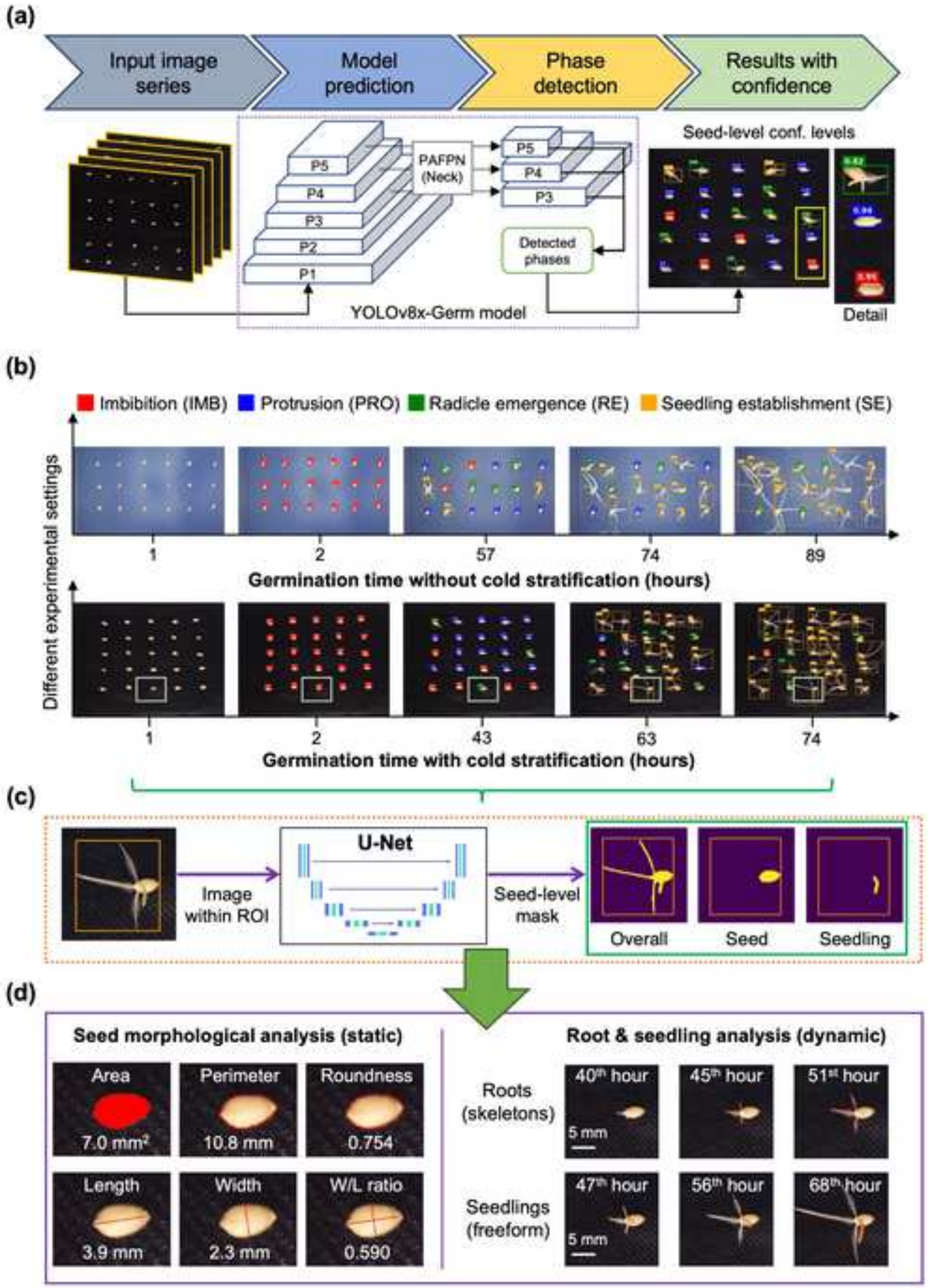

Figure 3 Click here to access/download;Figure;Slide3.jpeg

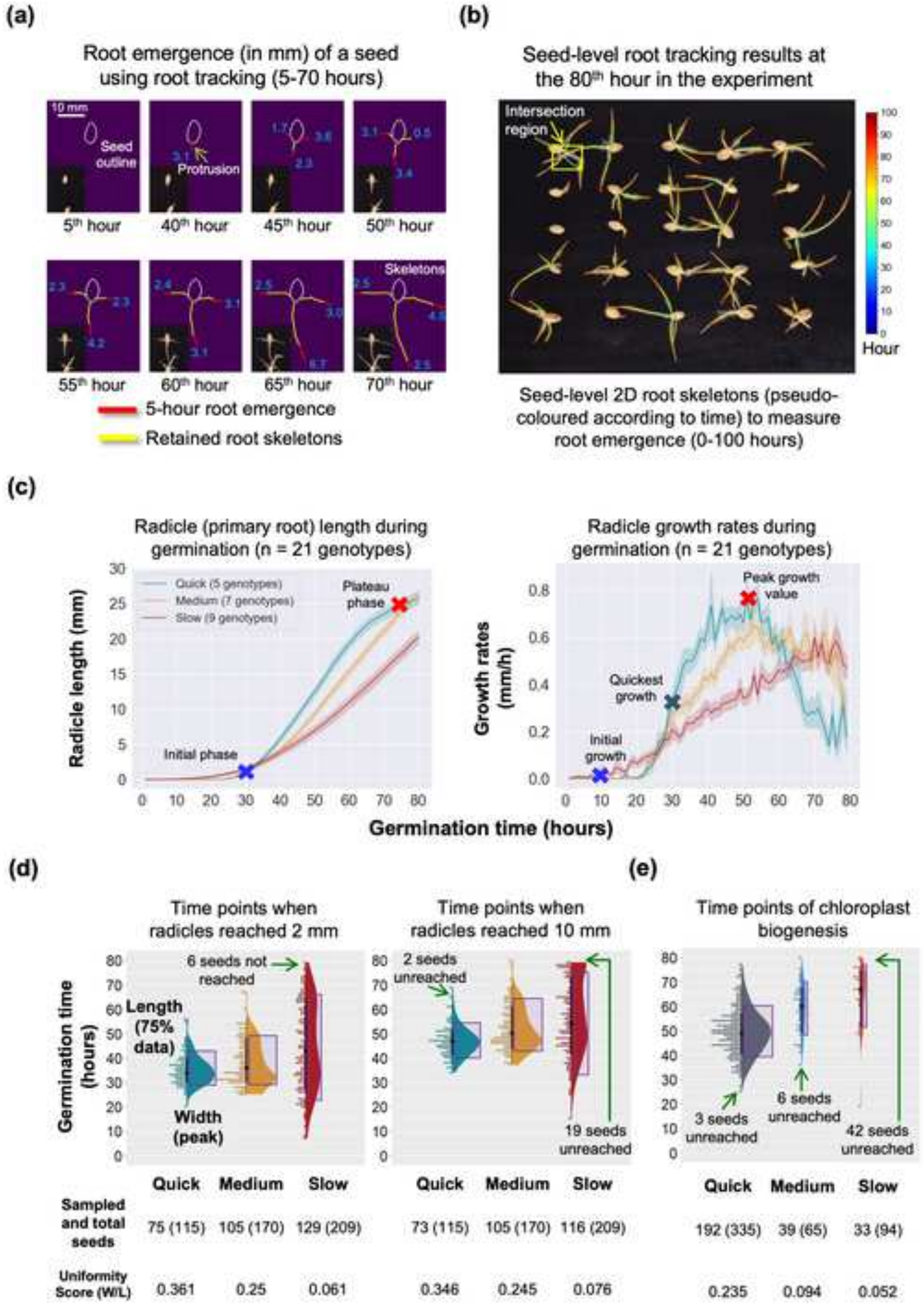

Figure 4

Click here to access/download;Figure;Slide4.jpeg

Genotypes	PRO phase (< 2 mm)		RE phase (2-10 mm)		SE phase (chloroplast biogenesis)		Overall vigour
	Speed	Uniformity	Speed	Uniformity	Speed	Uniformity	
G1	Slow	0.03	Slow	0.04	Slow	0.01	0.08
G2	Slow	0.02	Slow	0.01	Slow	0.01	0.04
G3	Medium	0.02	Slow	0.03	Slow	0.01	0.08
G4	Quick	0.31	Medium	0.29	Quick	0.04	1.63
G5	Quick	0.59	Quick	0.21	Quick	0.04	2.53
G6	Quick	0.23	Medium	0.22	Quick	0.10	1.43
G7	Medium	0.10	Medium	0.09	Quick	0.08	0.61
G8	Quick	0.33	Medium	0.30	Quick	0.10	1.90
G9	Quick	0.12	Slow	0.05	Quick	0.01	0.45
G10	Quick	0.14	Quick	0.09	Quick	0.04	0.80
G11	Quick	0.69	Medium	0.46	Quick	0.17	3.51
G12	Quick	0.16	Quick	0.09	Quick	0.02	0.82
G13	Medium	0.13	Medium	0.14	Medium	0.03	0.59
G14	Medium	0.04	Slow	0.04	Quick	0.02	0.19
G15	Quick	0.13	Slow	0.11	Quick	0.09	0.78
G16	Quick	0.02	Slow	0.02	Quick	0.02	0.13
G17	Medium	0.05	Medium	0.04	Medium	0.01	0.19
G18	Slow	0.02	Slow	0.01	Slow	0.01	0.04
G19	Slow	0.02	Slow	0.07	Slow	0.01	0.09
G20	Quick	0.04	Quick	0.02	Quick	0.01	0.19
G21	Quick	0.11	Quick	0.12	Medium	0.01	0.69

■ Slow speed or low uniformity ■ Medium speed or medium uniformity ■ Quick speed or high uniformity

Figure 5

Click here to access/download;Figure;Slide5.jpeg

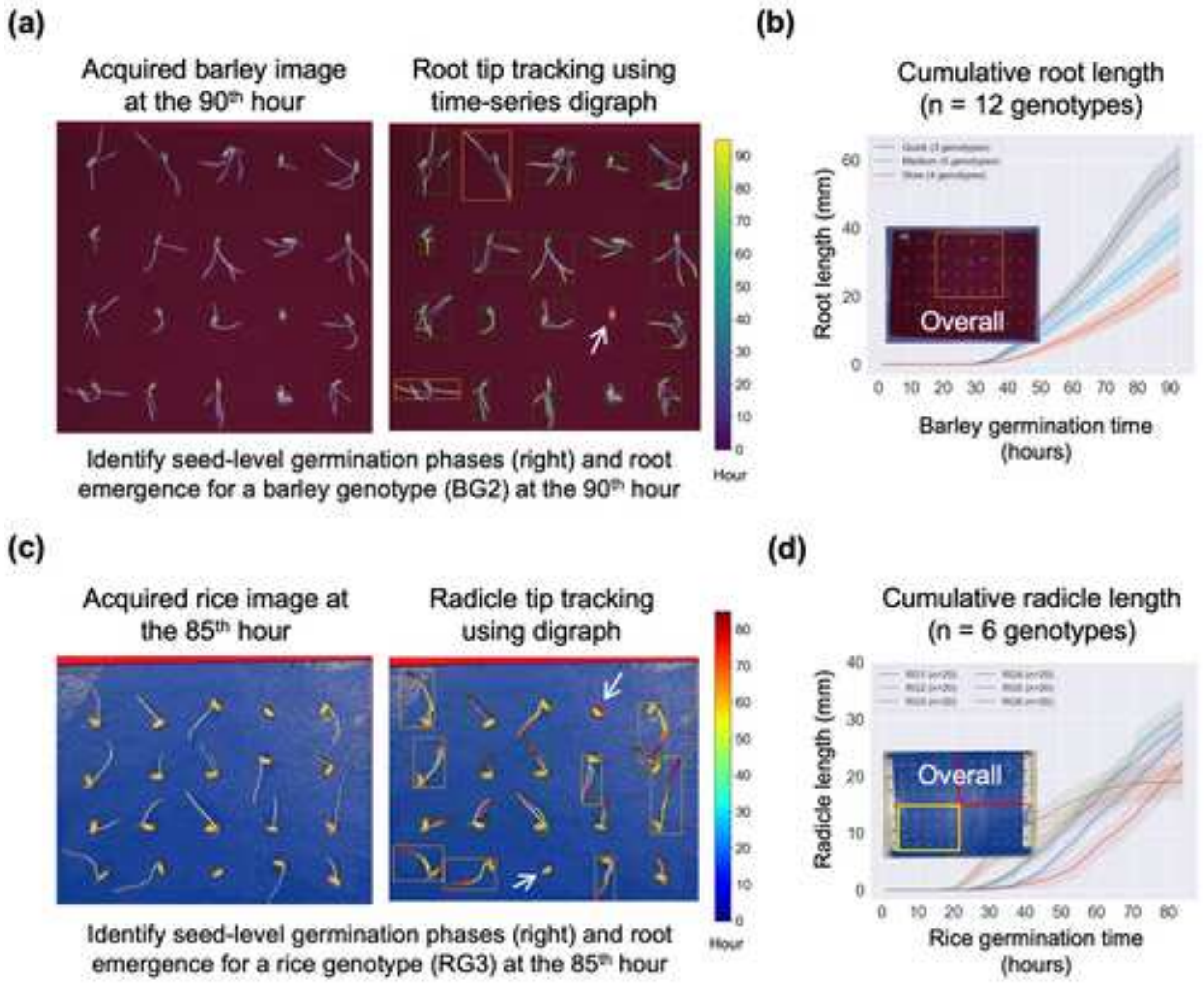

Click here to access/download
Supplementary Material
Supplementary Material_all-V1.1_JZ.docx

Response to reviewers:

Dear Reviewers and Handling Editor,

First, we would like to thank the three reviewers and the handling editor for their time and constructive comments. We apologise for the delay of our response due to a range of reasons! The first authors and I have spent sufficient time to go through our experiments, datasets, analysis results, and developed a graphic user interface (GUI) to enhance the quality of our manuscript.

As you will see from the markup version of the current manuscript, comments made by the reviewers have all been justified. Also, we invited an English senior scientist to go through the manuscript thoroughly, helping us improve the readability of the manuscript for scientists from different backgrounds.

A point-by-point response to the reviewers' comments (coloured red) are listed below with **line numbers based on the markup version** of the revised manuscript. Finally, I would like to take this opportunity to thank you again for your time and consideration for reviewing our manuscript, which assisted us to improve the quality and the presentation of our work!

Sincerely yours,

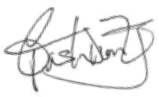

Ji Zhou

01-September-2025

Editor's comments

Please register any new software application in the bio.tools and SciCrunch.org databases to receive RRID (Research Resource Identification Initiative ID) and biotoolsID identifiers, and include these in your manuscript. Computational workflows should be registered in workflowhub.eu and the DOIs cited in the relevant places in the manuscript. These will facilitate tracking, reproducibility and re-use of your tool.

Response: Thanks for your suggestions. We have created a GUI using widget enabled by Jupyter notebook to facilitate non-expert accessing the SeedGerm-VIG (Lines 620-622 and Lines 238-241; Fig. S14). As we did not package the Jupyter notebook due to different runtime environments that different end users with varied operating systems might adopt, we therefore chose to share the modular source codes, Jupyter notebook files, DOME-ML and BioImage Archive (DOI: 10.6019/S-

BIAD1852) to respond to the editor's request.

Reviewer #1

The authors present a pipeline for the identification of growth stages in germinating seedlings. YOLO is used to identify seedlings and growth stages, a U-Net is used to segment these seedlings including the emerging root and shoot. The root is tracked over time using a graph approach, allowing temporal dynamics to be measured.
The paper does a good job of explaining the pipeline, and has a lot of tables of results showing various traits and experiments that have been conducted. Most of the work takes place on wheat, with smaller studies on other species. The work builds upon a previous work SeedGerm, which has attracted a good amount of interest, and as such I think there is demand for tools such as this. I have some suggestions that I think could improve the manuscript and its impact:
Response: Many thanks for your positive comments.

- The distinction between this work and it's predecessor was not completely clear to me. Seedgerm outlines the platform, but also machine learning derived traits? It would be helpful to be very clear what is new here.
Response: Thanks for your comments! We have now justified this question in Lines 374-378, stating that SeedGerm was developed for seed-lot level germination analysis, our study partially inherited its hardware design and seed-lot level source codes for analysis. Instead of traditional ML models, SeedGerm-VIG incorporated DL models into the pipeline to improve the generalisation and accuracy for **temporal seed-level analysis** and vigour-related feature assessment.

- The adaptation of the work to new species e.g. barley could be more clear. Did you re-train the network to account for the change in species and background? Or does the network work here without retraining? In which case the generality is impressive, and something to note in the paper.
Response: Thanks for your question! We used transfer learning to train learning models for rice and barely, with significantly reduced epochs and smaller training sets, suggesting how to apply DL techniques in future multi-species studies (see Line 409-412).

- The use of the phrase "optimised u-net" isn't common. What do you mean by optimised here? That it is trained? Or have you made changes to the architecture to make it more efficient? If not, the "u-net" is sufficient. Or "fine-tuned" if you wish to make clear it has been re-trained.
Response: Thanks for pointing it out! In the Materials and Methods section, we explained that our U-Net was based upon the optimised worked published by Hasal et al. (2023; Ref 40), which is not a standard U-Net (we improved wording in Line 534).

- Line 498, it isn't clear to me why you would train a unet for different numbers of epochs for different classes. Are there multiple unets being trained, or are the classes outputs from the same model? This was not clear.

Response: Thanks for pointing it out! In the Materials and Methods section, we explained that our trained three U-Net models (Line 536) for seed coat, seed/roots, and seedlings

- In its current form the pipeline is not hugely user friendly. The source.zip doesn't contain any code, this is stored in a different file. The code itself isn't on the github code repository at all, only in a zip file within the releases. The instructions are fine, but require a user with python and conda expertise. Once installed, the tool is primarily jupyter notebooks with many relative path strings which will require re-coding should the data change. There is no user interface except that provided by the notebook itself. For a programmer or machine learning expert, none of this will be a problem, but for those who are actually studying seed germination, this may not be practical. It is not obvious which code file to run first, and how to string the different bits of the pipeline together. Arguably there should be a single python file (either UI or command line interface) that exposes all of this functionality in a way where it is easy to run.

Response: We agree with the reviewer's comments. Still, given the lesson learned from SeedGerm, to package the Jupyter notebook with different runtime environments and libraries could easily cause problems to run the packaged sofware, if the libraries and end users' operating systems are upgraded. We therefore chose to share the modular source codes, Jupyter notebook files, DOME-ML, and BioImage Archive (for testing datasets), so that the users could choose what to utilise in their research.

To justify the reviewer's comments, we have created a GUI using widget enabled by Jupyter notebook to facilitate non-expert accessing the SeedGerm-VIG (Lines 620-622 and Lines 238-241; Fig. S14). Screenshots of the GUI can be seen as follows.

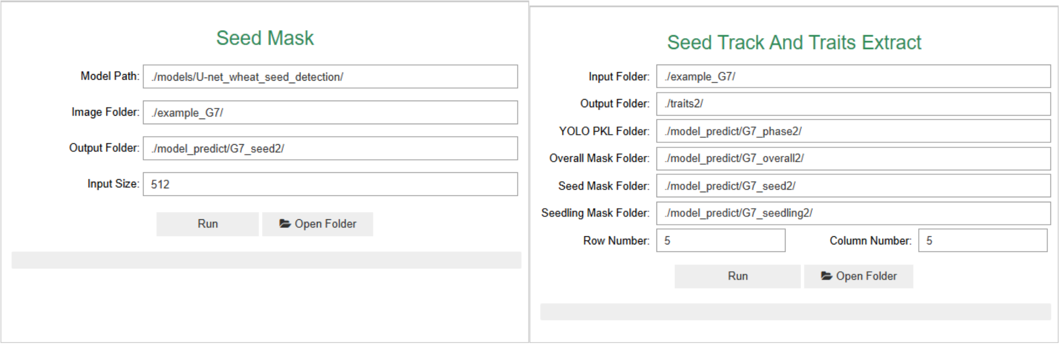

Page 71: "The sum of" doesn't make sense to me here. Do you mean "combination of"?

Response: Modified as requested (Line 72).

Reviewer #2

1. Summary

This manuscript introduces SeedGerm-VIG, an automated, open-source pipeline combining YOLOv8x and U-Net for assessing seed vigour using time-lapse phenotypic analysis. The pipeline is tested across wheat, barley, and rice, measuring both static and dynamic traits. A vigour scoring matrix is developed by integrating germination speed and uniformity across three phenological phases. The methodology is novel, and the application is timely and relevant to the plant phenotyping community.

2. Strengths

The integration of multiple deep learning models into a cohesive phenotyping pipeline is technically sound and innovative.

The manuscript presents strong validation using manual scoring, traditional software (SeedExtractor, SeedGerm), and heritability estimates.

The analysis includes a large dataset (29,500+ seed-level images), tested across 21 commercial genotypes.

The open-source release of code and data enhances reproducibility and community adoption.

Response: Many thanks for your positive comments! We really appreciate your supports!

3. Suggestions for Improvement

A. Deep Learning Model Choices:

While I agree that YOLO and U-Net are SOTA for the proposed technique, it is important to justify their use. Also, why was YOLOv8x specifically chosen when there are much more powerful models out there?

Response: Thanks for your comments! We have now made comparisons of different DL models' performance for seed detection (Table 2) and germination phases identification (Table 3). A line of discussion (Line 349) has also been included.

A. Statistical Analysis

The derivation of the uniformity score (W/L ratio of peak height to data coverage) lacks benchmarking. Suggest including comparisons with standard dispersion metrics such as IQR or coefficient of variation (CV).

Response: Thanks for your suggestion! We have added Supplementary Table S10 and S11and added Line 393 to justify this.

Confidence intervals (e.g., 75%) are shown in radicle growth plots (Fig. 3c) but the statistical basis for these is not explained. Please clarify if bootstrapping or parametric methods were used.

Response: Thanks for your comments! We used seaborn library and these were default parameters.

We have clarified in Line 616.

The clustering methods (e.g., affinity propagation, agglomerative clustering) are used without justification. A brief rationale for algorithm choice and cluster count selection is needed.
Response: Thanks for your suggestions! We selected clustering methods that automatically grouped data without requiring a predefined number of clusters, making them suitable for small- to medium-sized datasets (Lines 295-296). To verify the clustering results, we calculated the broad-sense heritability before and after clustering (Tables S2-S4) and found that all of them were above 0.9, indicating that after clustering, genetic effects could explain the vast majority of phenotypic variation in the 21 varieties, highlighting the essential differences between the 21 varieties.

B. Language and Grammar

Line 246: "growth rates did not follow a gradual and smooth increase pattern" could be rephrased for clarity.
Response: Corrected in Line 263.

Throughout: Several instances of overly informal tone (e.g., "nicely differentiate") should be revised to maintain scientific formality. Please conduct a grammar and tone check to ensure that the content is presented in a formal tone fit for a scientific paper.
Response: Thanks for pointing it out! Corrected in Line 396. A native English senior scientist was arranged to go through the manuscript as well.

C. Missing or Weak Analyses

The performance of the root tracking algorithm under conditions of seed/root movement or root intersection is not quantitatively assessed. Include failure case examples or tracking accuracy statistics (e.g., IoU or error rate).
Response: Thanks for your suggestion! When seeds and roots were moving, measures of the second and third roots were not very accurate (R^2 = ~0.4). We improved Fig. S4, Line 362/Lines 438-440, and added Note S2 to provide a complete evaluation.

Limited discussion is provided for how SeedGerm-VIG performs under suboptimal imaging conditions (e.g., occlusion, water reflection). These issues are mentioned in the Limitations (Line 399-416), but not supported with quantitative analysis or visual examples.
Response: Thanks for your suggestion! Note S2 and Lines 438-440 have now been added.

D. Image Augmentation:
The image augmentation techniques applied should be described to keep the reader more informed

(line 160).

Response: Improved in Line 161.

E. Future Study

Although this study computes seed morphometry using 2D images, the downsides of using 2D images has been well established. In the future, I suggest that the authors experiment with 3D imaging techniques. There are many low-cost turntables available in the market that could be coupled with the existing smartphones to capture a panoramic view of the seed for analysis.

Response: Thanks for your suggestions! Lines 452-454 have been added.

Reviewer #3

This manuscript by Dai et al. is a comprehensive and informative work. The process of data collection and analysis are described in good detail, so that on reading through the study most of the questions I thought of had been answered by the end of it. With regards to the requirements of GigaScience for data accessibility and transparency, I believe it fulfils the criteria well. Overall, I think it is an excellent paper thanks to the clear description of the work performed and the accessibility of the data associated with it. I found the figures and tables particularly informative and well-presented, and was grateful for the inclusion of extensive supplementary figures which further illustrated many of the author's points well.

Response: Many thanks for your supports and very positive comments!

I have a few minor comments that I think might improve the paper, but otherwise believe it is of excellent standard.

1.1. Computational reproducibility. Using the code and data that the authors provided, I attempted to reproduce some of the results in the paper. This was a little difficult as the structure of the github repository was not particularly clear. For example, the "release" page contains some of the jupyter notebooks but the main repository does not. Perhaps the jupyter notebooks could be added to the main repository? The same goes for the DL models. I had to add both directories to the repo after cloning it myself.

Response: Thanks for your suggestions! We have modified readme file in the GitHub repo for detailed explanation (https://github.com/The-Zhou-Lab/SeedGerm-VIG/blob/main/README.md).

1.2. After getting the code and models into the repo, the next issue was finding the appropriate version of python to use. I subsequently found that version 3.7 was specified in the methods, but it would be worth also indicating this in the repository.

Response: Thanks for your suggestions! Similarly, we have modified Readme.md to justify this.

1.3. Environments and packages. When installing some of the packages at the versions recorded in the repo, conflict errors arose. For example, between tensorflow 2.2. and scipy 1.7.3. Downgrading scipy fixed this issue, but perhaps the authors could create a virtual environment and a requirements.txt file to list python and package versions which work together.
Response: Thanks! We have modified Readme.md to justify this request.

1.4. Most of the notebooks ran without issues, however there were a few errors. The mask_prediction.ipynb ran but produced images without masks. This may just be an issue reproducing the analysis on a new machine, but I would be grateful if the authors could check that this notebook runs correctly on other computers.
Response: Thanks for trialling our work! We have now improved the .ipynb file and uploaded to the GitHub. Please do use the URL (https://github.com/The-Zhou-Lab/SeedGerm-VIG/releases) to access the latest version of the uploaded notebook file.

1.5. The authors could add a note to tell others to run phase_detection.ipynb before the phase_correction_and_quantification.ipynb if they do not have GPU support for pytorch (as I didn't), to avoid errors about using CPU instead of GPU. Or perhaps just add a note about setting up pytorch with GPU.
Response: Thanks for your comments! We have modified Readme.md to justify this request.

Overall, the notebooks were easy to follow and reproduce. There were some minor differences in results, which could maybe be mitigated by specifying a seed when running the analysis if that is possible. The main improvement would be to document the environment in more detail, and perhaps add greater detail on how to run the notebooks.
Response: Thanks for your comments! We have modified Readme.md to justify this request.

2. The only other significant question I have for the authors is regarding the DL models. These are described in the methods, but I would be interested to know more detail. For example, would it be possible for the authors to share the code used to build these models? I'm aware that the models themselves were included as part of the github release, but I could not easily find information on how exactly they were built. If the authors could share the code and/or other detail regarding how the DL models were built I believe that would be very helpful. The model performance figures in the supplementary are useful to see how well they perform, but if others would like to develop their own DL models or know more about the process I believe it is hard to find that information at the moment.
Response: Thanks for your comments! We have listed all of the libraries in the Readme.md.

3. My last question is only very minor and relates to how SeedGerm-VIG is connected to SeedGerm. Was the original SeedGerm code used in this project? Or was it more the concept of seed

germination analysis that is being extended here?

Response: Please refer to our answers to the similar question raised by Reviewer #1 (Lines 374-378). SeedGerm was developed for seed-lot level germination analysis, our study partially inherited its hardware design and seed-lot level source codes for analysis. Instead of traditional ML models, SeedGerm-VIG incorporated DL models into the pipeline to improve the generalisation and accuracy for **temporal seed-level analysis** and vigour-related feature assessment.

Aside from these minor comments/questions, I think the manuscript was very well presented and that the pipeline could be very useful for breeders with an interest in developing germplasm with improved seed vigour (if they can set it up and use it).

Response: A GUI interface has been added in this version of manuscript.
