# Supplementary material for: SeedGerm-VIG: an open and comprehensive pipeline to quantify seed vigor in wheat and other cereal crops using deep learning–powered dynamic phenotypic analysis
Source: Gigascience. 2025 Oct 16;14:giaf129. doi: 10.1093/gigascience/giaf129 (PMC12648739; doi:10.1093/gigascience/giaf129)
Supplement: giaf129_Authors_Response_To_Reviewer_Comments_Original_Submission [file giaf129_authors_response_to_reviewer_comments_original_submission.pdf]

Author's Response To Reviewer Comments

Editor’s comments
Please register any new software application in the bio.tools and SciCrunch.org databases to receive RRID (Research Resource Identification Initiative ID) and biotoolsID identifiers, and include these in your manuscript. Computational workflows should be registered in workflowhub.eu and the DOIs cited in the relevant places in the manuscript. These will facilitate tracking, reproducibility and re-use of your tool.
Response: Thanks for your suggestions. We have created a GUI using widget enabled by Jupyter notebook to facilitate non-expert accessing the SeedGerm-VIG (Lines 620-622 and Lines 238-241; Fig. S14). As we did not package the Jupyter notebook due to different runtime environments that different end users with varied operating systems might adopt, we therefore chose to share the modular source codes, Jupyter notebook files, DOME-ML and BioImage Archive (DOI: 10.6019/S-BIAD1852) to respond to the editor’s request.

Reviewer #1
The authors present a pipeline for the identification of growth stages in germinating seedlings. YOLO is used to identify seedlings and growth stages, a U-Net is used to segment these seedlings including the emerging root and shoot. The root is tracked over time using a graph approach, allowing temporal dynamics to be measured.
The paper does a good job of explaining the pipeline, and has a lot of tables of results showing various traits and experiments that have been conducted. Most of the work takes place on wheat, with smaller studies on other species. The work builds upon a previous work SeedGerm, which has attracted a good amount of interest, and as such I think there is demand for tools such as this. I have some suggestions that I think could improve the manuscript and its impact:
Response: Many thanks for your positive comments.

- The distinction between this work and it's predecessor was not completely clear to me. Seedgerm outlines the platform, but also machine learning derived traits? It would be helpful to be very clear what is new here.
Response: Thanks for your comments! We have now justified this question in Lines 374-378, stating that SeedGerm was developed for seed-lot level germination analysis, our study partially inherited its hardware design and seed-lot level source codes for analysis. Instead of traditional ML models, SeedGerm-VIG incorporated DL models into the pipeline to improve the generalisation and accuracy for temporal seed-level analysis and vigour-related feature assessment.

- The adaptation of the work to new species e.g. barley could be more clear. Did you re-train the network to account for the change in species and background? Or does the network work here without retraining? In which case the generality is impressive, and something to note in the paper.
Response: Thanks for your question! We used transfer learning to train learning models for rice and barely, with significantly reduced epochs and smaller training sets, suggesting how to apply DL techniques in future multi-species studies (see Line 409-412).

- The use of the phrase "optimised u-net" isn't common. What do you mean by optimised here? That it is trained? Or have you made changes to the architecture to make it more efficient? If not, the "u-net" is sufficient. Or "fine-tuned" if you wish to make clear it has been re-trained.
Response: Thanks for pointing it out! In the Materials and Methods section, we explained that our U-Net was based upon the optimised worked published by Hasal et al. (2023; Ref 40), which is not a standard U-Net (we improved wording in Line 534).

- Line 498, it isn't clear to me why you would train a unet for different numbers of epochs for different classes. Are there multiple unets being trained, or are the classes outputs from the same model? This was not clear.
Response: Thanks for pointing it out! In the Materials and Methods section, we explained that our trained three U-Net models (Line 536) for seed coat, seed/roots, and seedlings

- In its current form the pipeline is not hugely user friendly. The source.zip doesn't contain any code, this is stored in a different file. The code itself isn't on the github code repository at all, only in a zip file within the releases. The instructions are fine, but require a user with python and conda expertise. Once installed, the tool is primarily jupyter notebooks with many relative path strings which will require re-coding should the data change. There is no user interface except that provided by the notebook itself. For a programmer or machine learning expert, none of this will be a problem, but for those who are actually studying seed germination, this may not be practical. It is not obvious which code file to run first, and how to string the different bits of the pipeline together. Arguably there should be a single python file (either UI or command line interface) that exposes all of this functionality in a way where it is easy to run.
Response: We agree with the reviewer's comments. Still, given the lesson learned from SeedGerm, to package the Jupyter notebook with different runtime environments and libraries could easily cause problems to run the packaged sofware, if the libraries and end users' operating systems are upgraded. We therefore chose to share the modular source codes, Jupyter notebook files, DOME-ML, and BioImage Archive (for testing datasets), so that the users could choose what to utilise in their research.
To justify the reviewer's comments, we have created a GUI using widget enabled by Jupyter notebook to facilitate non-expert accessing the SeedGerm-VIG (Lines 620-622 and Lines 238-241; Fig. S14). Screenshots of the GUI can be seen as follows.

Page 71: "The sum of" doesn't make sense to me here. Do you mean "combination of"?
Response: Modified as requested (Line 72).

Reviewer #2
1. Summary
This manuscript introduces SeedGerm-VIG, an automated, open-source pipeline combining YOLOv8x and U-Net for assessing seed vigour using time-lapse phenotypic analysis. The pipeline is tested across wheat, barley, and rice, measuring both static and dynamic traits. A vigour scoring matrix is developed by integrating germination speed and uniformity across three phenological phases. The methodology is novel, and the application is timely and relevant to the plant phenotyping community.

2. Strengths
The integration of multiple deep learning models into a cohesive phenotyping pipeline is technically sound and innovative.
The manuscript presents strong validation using manual scoring, traditional software (SeedExtractor, SeedGerm), and heritability estimates.
The analysis includes a large dataset (29,500+ seed-level images), tested across 21 commercial genotypes.
The open-source release of code and data enhances reproducibility and community adoption.
Response: Many thanks for your positive comments! We really appreciate your supports!

3. Suggestions for Improvement
A. Deep Learning Model Choices:
While I agree that YOLO and U-Net are SOTA for the proposed technique, it is important to justify their use. Also, why was YOLOv8x specifically chosen when there are much more powerful models out there?
Response: Thanks for your comments! We have now made comparisons of different DL models' performance for seed detection (Table 2) and germination phases identification (Table 3). A line of discussion (Line 349) has also been included.

A. Statistical Analysis
The derivation of the uniformity score (W/L ratio of peak height to data coverage) lacks benchmarking. Suggest including comparisons with standard dispersion metrics such as IQR or coefficient of variation (CV).
Response: Thanks for your suggestion! We have added Supplementary Table S10 and S11and added Line 393 to justify this.

Confidence intervals (e.g., 75%) are shown in radicle growth plots (Fig. 3c) but the statistical basis for these is not explained. Please clarify if bootstrapping or parametric methods were used.

Response: Thanks for your comments! We used seaborn library and these were default parameters. We have clarified in Line 616.

The clustering methods (e.g., affinity propagation, agglomerative clustering) are used without justification. A brief rationale for algorithm choice and cluster count selection is needed.
Response: Thanks for your suggestions! We selected clustering methods that automatically grouped data without requiring a predefined number of clusters, making them suitable for small- to medium-sized datasets (Lines 295-296). To verify the clustering results, we calculated the broad-sense heritability before and after clustering (Tables S2-S4) and found that all of them were above 0.9, indicating that after clustering, genetic effects could explain the vast majority of phenotypic variation in the 21 varieties, highlighting the essential differences between the 21 varieties.

B. Language and Grammar

Line 246: "growth rates did not follow a gradual and smooth increase pattern" could be rephrased for clarity.
Response: Corrected in Line 263.

Throughout: Several instances of overly informal tone (e.g., "nicely differentiate") should be revised to maintain scientific formality. Please conduct a grammar and tone check to ensure that the content is presented in a formal tone fit for a scientific paper.
Response: Thanks for pointing it out! Corrected in Line 396. A native English senior scientist was arranged to go through the manuscript as well.

C. Missing or Weak Analyses

The performance of the root tracking algorithm under conditions of seed/root movement or root intersection is not quantitatively assessed. Include failure case examples or tracking accuracy statistics (e.g., IoU or error rate).
Response: Thanks for your suggestion! When seeds and roots were moving, measures of the second and third roots were not very accurate (R2 = ~0.4). We improved Fig. S4, Line 362/Lines 438-440, and added Note S2 to provide a complete evaluation.

Limited discussion is provided for how SeedGerm-VIG performs under suboptimal imaging conditions (e.g., occlusion, water reflection). These issues are mentioned in the Limitations (Line 399-416), but not supported with quantitative analysis or visual examples.
Response: Thanks for your suggestion! Note S2 and Lines 438-440 have now been added.

D. Image Augmentation:
The image augmentation techniques applied should be described to keep the reader more informed (line 160).
Response: Improved in Line 161.

E. Future Study
Although this study computes seed morphometry using 2D images, the downsides of using 2D images has been well established. In the future, I suggest that the authors experiment with 3D imaging techniques. There are many low-cost turntables available in the market that could be coupled with the existing smartphones to capture a panoramic view of the seed for analysis.
Response: Thanks for your suggestions! Lines 452-454 have been added.

Reviewer #3
This manuscript by Dai et al. is a comprehensive and informative work. The process of data collection and analysis are described in good detail, so that on reading through the study most of the questions I thought of had been answered by the end of it. With regards to the requirements of GigaScience for data accessibility and transparency, I believe it fulfils the criteria well. Overall, I think it is an excellent paper thanks to the clear description of the work performed and the accessibility of the data associated with it. I found the figures and tables particularly informative and well-presented, and was grateful for the inclusion

of extensive supplementary figures which further illustrated many of the author's points well.
Response: Many thanks for your supports and very positive comments!

I have a few minor comments that I think might improve the paper, but otherwise believe it is of excellent standard.

1.1. Computational reproducibility. Using the code and data that the authors provided, I attempted to reproduce some of the results in the paper. This was a little difficult as the structure of the github repository was not particularly clear. For example, the "release" page contains some of the jupyter notebooks but the main repository does not. Perhaps the jupyter notebooks could be added to the main repository? The same goes for the DL models. I had to add both directories to the repo after cloning it myself.
Response: Thanks for your suggestions! We have modified readme file in the GitHub repo for detailed explanation (https://github.com/The-Zhou-Lab/SeedGerm-VIG/blob/main/README.md).

1.2. After getting the code and models into the repo, the next issue was finding the appropriate version of python to use. I subsequently found that version 3.7 was specified in the methods, but it would be worth also indicating this in the repository.
Response: Thanks for your suggestions! Similarly, we have modified Readme.md to justify this.
1.3. Environments and packages. When installing some of the packages at the versions recorded in the repo, conflict errors arose. For example, between tensorflow 2.2. and scipy 1.7.3. Downgrading scipy fixed this issue, but perhaps the authors could create a virtual environment and a requirements.txt file to list python and package versions which work together.
Response: Thanks! We have modified Readme.md to justify this request.

1.4. Most of the notebooks ran without issues, however there were a few errors. The mask_prediction.ipynb ran but produced images without masks. This may just be an issue reproducing the analysis on a new machine, but I would be grateful if the authors could check that this notebook runs correctly on other computers.
Response: Thanks for trialling our work! We have now improved the .ipynb file and uploaded to the GitHub. Please do use the URL (https://github.com/The-Zhou-Lab/SeedGerm-VIG/releases) to access the latest version of the uploaded notebook file.

1.5. The authors could add a note to tell others to run phase_detection.ipynb before the phase_correction_and_quantification.ipynb if they do not have GPU support for pytorch (as I didn't), to avoid errors about using CPU instead of GPU. Or perhaps just add a note about setting up pytorch with GPU.
Response: Thanks for your comments! We have modified Readme.md to justify this request.

Overall, the notebooks were easy to follow and reproduce. There were some minor differences in results, which could maybe be mitigated by specifying a seed when running the analysis if that is possible. The main improvement would be to document the environment in more detail, and perhaps add greater detail on how to run the notebooks.
Response: Thanks for your comments! We have modified Readme.md to justify this request.

2. The only other significant question I have for the authors is regarding the DL models. These are described in the methods, but I would be interested to know more detail. For example, would it be possible for the authors to share the code used to build these models? I'm aware that the models themselves were included as part of the github release, but I could not easily find information on how exactly they were built. If the authors could share the code and/or other detail regarding how the DL models were built I believe that would be very helpful. The model performance figures in the supplementary are useful to see how well they perform, but if others would like to develop their own DL models or know more about the process I believe it is hard to find that information at the moment.
Response: Thanks for your comments! We have listed all of the libraries in the Readme.md.

3. My last question is only very minor and relates to how SeedGerm-VIG is connected to SeedGerm. Was the original SeedGerm code used in this project? Or was it more the concept of seed germination analysis

that is being extended here?
Response: Please refer to our answers to the similar question raised by Reviewer #1 (Lines 374-378). SeedGerm was developed for seed-lot level germination analysis, our study partially inherited its hardware design and seed-lot level source codes for analysis. Instead of traditional ML models, SeedGerm-VIG incorporated DL models into the pipeline to improve the generalisation and accuracy for temporal seed-level analysis and vigour-related feature assessment.

Aside from these minor comments/questions, I think the manuscript was very well presented and that the pipeline could be very useful for breeders with an interest in developing germplasm with improved seed vigour (if they can set it up and use it).
Response: A GUI interface has been added in this version of manuscript.

Editorial Manager®

Cookie Preference Center

We use cookies which are necessary to make our site work. We may also use additional cookies to analyze, improve and personalize our content and your digital experience. For more information, see our Cookie Policy.

You may choose not to allow some types of cookies. However, blocking some types may impact your experience of our site and the services we are able to offer. See the different category headings below to find out more or change your settings.

Allow all

Manage Consent Preferences

Strictly Necessary Cookies

Always active

These cookies are necessary for the website to function and cannot be switched off in our systems. They are usually only set in response to actions made by you which amount to a request for services, such as setting your privacy preferences, logging in or filling in forms. You can set your browser to block or alert you about these cookies, but some parts of the site will not then work.

Functional Cookies

These cookies enable the website to provide enhanced functionality and personalisation. They may be set by us or by third party providers whose services we have added to our pages. If you do not allow these cookies then some or all of these services may not function properly.

Performance Cookies

These cookies allow us to count visits and traffic sources so we can measure and improve the performance of our site. They help us to know which pages are the most and least popular and see how visitors move around the site.

Targeting Cookies

These cookies may be set through our site by our advertising partners. They may be used by those companies to build a profile of your interests and show you relevant adverts on other sites. If you do not allow these cookies, you will experience less targeted advertising.

Cookie List

Clear

Apply Cancel
Consent Leg.Interest

Confirm my choices

Powered by onetrus
