# Supplementary material for: SeedGerm-VIG: an open and comprehensive pipeline to quantify seed vigor in wheat and other cereal crops using deep learning–powered dynamic phenotypic analysis
Source: Gigascience. 2025 Oct 16;14:giaf129. doi: 10.1093/gigascience/giaf129 (PMC12648739; doi:10.1093/gigascience/giaf129)
Supplement: giaf129_Reviewer_1_Report_Original_Submission — Michael Pound -- 5/11/2025 [file giaf129_reviewer_1_report_original_submission.pdf]

**Reviewer Report**

**Title: SeedGerm-VIG: an open and comprehensive pipeline to quantify seed vigour in wheat and other cereal crops using deep learning powered dynamic phenotypic analysis**

**Version: Original Submission** **Date:** 5/11/2025

**Reviewer name: Michael Pound**

**Reviewer Comments to Author:**

The authors present a pipeline for the identification of growth stages in germinating seedlings. YOLO is used to identify seedlings and growth stages, a U-Net is used to segment these seedlings including the emerging root and shoot. The root is tracked over time using a graph approach, allowing temporal dynamics to be measured.

The paper does a good job of explaining the pipeline, and has a lot of tables of results showing various traits and experiments that have been conducted. Most of the work takes place on wheat, with smaller studies on other species. The work builds upon a previous work SeedGerm, which has attracted a good amount of interest, and as such I think there is demand for tools such as this. I have some suggestions that I think could improve the manuscript and its impact:

- The distinction between this work and it's predecessor was not completely clear to me. Seedgerm outlines the platform, but also machine learning derived traits? It would be helpful to be very clear what is new here.
- The adaptation of the work to new species e.g. barley could be more clear. Did you re-train the network to account for the change in species and background? Or does the network work here without retraining? In which case the generality is impressive, and something to note in the paper.
- The use of the phrase "optimised u-net" isn't common. What do you mean by optimised here? That it is trained? Or have you made changes to the architecture to make it more efficient? If not, the "u-net" is sufficient. Or "fine-tuned" if you wish to make clear it has been re-trained.
- Line 498, it isn't clear to me why you would train a unet for different numbers of epochs for different classes. Are there multiple unets being trained, or are the classes outputs from the same model? This was not clear.
- In its current form the pipeline is not hugely user friendly. The source.zip doesn't contain any code, this is stored in a different file. The code itself isn't on the github code repository at all, only in a zip file within the releases. The instructions are fine, but require a user with python and conda expertise. Once installed, the tool is primarily jupyter notebooks with many relative path strings which will require re-coding should the data change. There is no user interface except that provided by the notebook itself. For a programmer or machine learning expert, none of this will be a problem, but for those who are actually studying seed germination, this may not be practical. It is not obvious which code file to run first, and how to string the different bits of the pipeline together. Arguably there should be a single python file (either UI or command line interface) that exposes all of this functionality in a way where it is easy to run.

Page 71: "The sum of" doesn't make sense to me here. Do you mean "combination of"?

**Methods**

Are the methods appropriate to the aims of the study, are they well described, and are necessary controls included? Choose an item.

**Conclusions**

Are the conclusions adequately supported by the data shown? Choose an item.

**Reporting Standards**

Does the manuscript adhere to the journal's guidelines on minimum standards of reporting? Choose an item.

Choose an item.

**Statistics**

Are you able to assess all statistics in the manuscript, including the appropriateness of statistical tests used? Choose an item.

**Quality of Written English**

Please indicate the quality of language in the manuscript: Choose an item.

**Declaration of Competing Interests**

Please complete a declaration of competing interests, considering the following questions:

- Have you in the past five years received reimbursements, fees, funding, or salary from an organisation that may in any way gain or lose financially from the publication of this manuscript, either now or in the future?
- Do you hold any stocks or shares in an organisation that may in any way gain or lose financially from the publication of this manuscript, either now or in the future?
- Do you hold or are you currently applying for any patents relating to the content of the manuscript?
- Have you received reimbursements, fees, funding, or salary from an organization that holds or has applied for patents relating to the content of the manuscript?
- Do you have any other financial competing interests?
- Do you have any non-financial competing interests in relation to this paper?

If you can answer no to all of the above, write 'I declare that I have no competing interests' below. If your reply is yes to any, please give details below.

I declare that I have no competing interests

I agree to the open peer review policy of the journal. I understand that my name will be included on my report to the authors and, if the manuscript is accepted for publication, my named report including any attachments I upload will be posted on the website along with the authors' responses. I agree for my report to be made available under an Open Access Creative Commons CC-BY license (http://creativecommons.org/licenses/by/4.0/). I understand that any comments which I do not wish to be included in my named report can be included as confidential comments to the editors, which will not be published.

Choose an item.

To further support our reviewers, we have joined with Publons, where you can gain additional credit to further highlight your hard work (see: https://publons.com/journal/530/gigascience). On publication of this paper, your review will be automatically added to Publons, you can then choose whether or not to claim your Publons credit. I understand this statement.

Yes Choose an item.
