# Supplementary material for: SeedGerm-VIG: an open and comprehensive pipeline to quantify seed vigor in wheat and other cereal crops using deep learning–powered dynamic phenotypic analysis
Source: Gigascience. 2025 Oct 16;14:giaf129. doi: 10.1093/gigascience/giaf129 (PMC12648739; doi:10.1093/gigascience/giaf129)
Supplement: giaf129_Reviewer_2_Report_Original_Submission — Venkat Margapuri -- 5/22/2025 [file giaf129_reviewer_2_report_original_submission.pdf]

Reviewer Report

Title: SeedGerm-VIG: an open and comprehensive pipeline to quantify seed vigour in wheat and other cereal crops using deep learning powered dynamic phenotypic analysis

Version: Original Submission **Date:** 5/22/2025

Reviewer name: Venkat Margapuri

Reviewer Comments to Author:

1. Summary
This manuscript introduces SeedGerm-VIG, an automated, open-source pipeline combining YOLOv8x and U-Net for assessing seed vigour using time-lapse phenotypic analysis. The pipeline is tested across wheat, barley, and rice, measuring both static and dynamic traits. A vigour scoring matrix is developed by integrating germination speed and uniformity across three phenological phases. The methodology is novel, and the application is timely and relevant to the plant phenotyping community.
2. Strengths
The integration of multiple deep learning models into a cohesive phenotyping pipeline is technically sound and innovative.
The manuscript presents strong validation using manual scoring, traditional software (SeedExtractor, SeedGerm), and heritability estimates.
The analysis includes a large dataset (29,500+ seed-level images), tested across 21 commercial genotypes.
The open-source release of code and data enhances reproducibility and community adoption.
3. Suggestions for Improvement
A. Deep Learning Model Choices:
While I agree that YOLO and U-Net are SOTA for the proposed technique, it is important to justify their use. Also, why was YOLOv8x specifically chosen when there are much more powerful models out there?
A. Statistical Analysis
The derivation of the uniformity score (W/L ratio of peak height to data coverage) lacks benchmarking. Suggest including comparisons with standard dispersion metrics such as IQR or coefficient of variation (CV).
Confidence intervals (e.g., 75%) are shown in radicle growth plots (Fig. 3c) but the statistical basis for these is not explained. Please clarify if bootstrapping or parametric methods were used.
The clustering methods (e.g., affinity propagation, agglomerative clustering) are used without justification. A brief rationale for algorithm choice and cluster count selection is needed.
B. Language and Grammar
Line 246: "growth rates did not follow a gradual and smooth increase pattern" could be rephrased for clarity.
Throughout: Several instances of overly informal tone (e.g., "nicely differentiate") should be revised to maintain scientific formality. Please conduct a grammar and tone check to ensure that the content is presented in a formal tone fit for a scientific paper.

C. Missing or Weak Analyses
The performance of the root tracking algorithm under conditions of seed/root movement or root intersection is not quantitatively assessed. Include failure case examples or tracking accuracy statistics (e.g., IoU or error rate).
Limited discussion is provided for how SeedGerm-VIG performs under suboptimal imaging conditions (e.g., occlusion, water reflection). These issues are mentioned in the Limitations (Line 399-416), but not supported with quantitative analysis or visual examples.
D. Image Augmentation:
The image augmentation techniques applied should be described to keep the reader more informed (line 160).
E. Future Study
Although this study computes seed morphometry using 2D images, the downsides of using 2D images has been well established. In the future, I suggest that the authors experiment with 3D imaging techniques. There are many low-cost turntables available in the market that could be coupled with the existing smartphones to capture a panoramic view of the seed for analysis.

Methods

Are the methods appropriate to the aims of the study, are they well described, and are necessary controls included? Choose an item.

Conclusions

Are the conclusions adequately supported by the data shown? Choose an item.

Reporting Standards

Does the manuscript adhere to the journal's guidelines on minimum standards of reporting? Choose an item.

Choose an item.

Statistics

Are you able to assess all statistics in the manuscript, including the appropriateness of statistical tests used? Choose an item.

Quality of Written English

Please indicate the quality of language in the manuscript: Choose an item.

Declaration of Competing Interests

Please complete a declaration of competing interests, considering the following questions:

- Have you in the past five years received reimbursements, fees, funding, or salary from an organisation that may in any way gain or lose financially from the publication of this manuscript, either now or in the future?
- Do you hold any stocks or shares in an organisation that may in any way gain or lose financially from the publication of this manuscript, either now or in the future?
- Do you hold or are you currently applying for any patents relating to the content of the manuscript?
- Have you received reimbursements, fees, funding, or salary from an organization that holds or has applied for patents relating to the content of the manuscript?
- Do you have any other financial competing interests?
- Do you have any non-financial competing interests in relation to this paper?

If you can answer no to all of the above, write 'I declare that I have no competing interests' below. If your reply is yes to any, please give details below.

I declare that I have no competing interests

I agree to the open peer review policy of the journal. I understand that my name will be included on my report to the authors and, if the manuscript is accepted for publication, my named report including any attachments I upload will be posted on the website along with the authors' responses. I agree for my report to be made available under an Open Access Creative Commons CC-BY license (http://creativecommons.org/licenses/by/4.0/). I understand that any comments which I do not wish to be included in my named report can be included as confidential comments to the editors, which will not be published.

Choose an item.

To further support our reviewers, we have joined with Publons, where you can gain additional credit to further highlight your hard work (see: https://publons.com/journal/530/gigascience). On publication of this paper, your review will be automatically added to Publons, you can then choose whether or not to claim your Publons credit. I understand this statement.

Yes Choose an item.
