# Supplementary material for: SeedGerm-VIG: an open and comprehensive pipeline to quantify seed vigor in wheat and other cereal crops using deep learning–powered dynamic phenotypic analysis
Source: Gigascience. 2025 Oct 16;14:giaf129. doi: 10.1093/gigascience/giaf129 (PMC12648739; doi:10.1093/gigascience/giaf129)
Supplement: giaf129_Reviewer_3_Report_Original_Submission — Joseph Oddy -- 5/27/2025 [file giaf129_reviewer_3_report_original_submission.pdf]

Reviewer Report

Title: SeedGerm-VIG: an open and comprehensive pipeline to quantify seed vigour in wheat and other cereal crops using deep learning powered dynamic phenotypic analysis

Version: Original Submission **Date:** 5/27/2025

Reviewer name: Joseph Oddy

Reviewer Comments to Author:

This manuscript by Dai et al. is a comprehensive and informative work. The process of data collection and analysis are described in good detail, so that on reading through the study most of the questions I thought of had been answered by the end of it. With regards to the requirements of GigaScience for data accessibility and transparency, I believe it fulfils the criteria well. Overall, I think it is an excellent paper thanks to the clear description of the work performed and the accessibility of the data associated with it. I found the figures and tables particularly informative and well-presented, and was grateful for the inclusion of extensive supplementary figures which further illustrated many of the author's points well.

I have a few minor comments that I think might improve the paper, but otherwise believe it is of excellent standard.

1.1. Computational reproducibility. Using the code and data that the authors provided, I attempted to reproduce some of the results in the paper. This was a little difficult as the structure of the github repository was not particularly clear. For example, the "release" page contains some of the jupyter notebooks but the main repository does not. Perhaps the jupyter notebooks could be added to the main repository? The same goes for the DL models. I had to add both directories to the repo after cloning it myself.

1.2. After getting the code and models into the repo, the next issue was finding the appropriate version of python to use. I subsequently found that version 3.7 was specified in the methods, but it would be worth also indicating this in the repository.

1.3. Environments and packages. When installing some of the packages at the versions recorded in the repo, conflict errors arose. For example, between tensorflow 2.2. and scipy 1.7.3. Downgrading scipy fixed this issue, but perhaps the authors could create a virtual environment and a requirements.txt file to list python and package versions which work together.

1.4. Most of the notebooks ran without issues, however there were a few errors. The mask_prediction.ipynb ran but produced images without masks. This may just be an issue reproducing the analysis on a new machine, but I would be grateful if the authors could check that this notebook runs correctly on other computers.

1.5. The authors could add a note to tell others to run phase_detection.ipynb before the phase_correction_and_quantification.ipynb if they do not have GPU support for pytorch (as I didn't), to avoid errors about using CPU instead of GPU. Or perhaps just add a note about setting up pytorch with GPU.

Overall, the notebooks were easy to follow and reproduce. There were some minor differences in

results, which could maybe be mitigated by specifying a seed when running the analysis if that is possible. The main improvement would be to document the environment in more detail, and perhaps add greater detail on how to run the notebooks.
2. The only other significant question I have for the authors is regarding the DL models. These are described in the methods, but I would be interested to know more detail. For example, would it be possible for the authors to share the code used to build these models? I'm aware that the models themselves were included as part of the github release, but I could not easily find information on how exactly they were built. If the authors could share the code and/or other detail regarding how the DL models were built I believe that would be very helpful. The model performance figures in the supplementary are useful to see how well they perform, but if others would like to develop their own DL models or know more about the process I believe it is hard to find that information at the moment.
3. My last question is only very minor and relates to how SeedGerm-VIG is connected to SeedGerm. Was the original SeedGerm code used in this project? Or was it more the concept of seed germination analysis that is being extended here?
Aside from these minor comments/questions, I think the manuscript was very well presented and that the pipeline could be very useful for breeders with an interest in developing germplasm with improved seed vigour (if they can set it up and use it).

Methods

Are the methods appropriate to the aims of the study, are they well described, and are necessary controls included? Choose an item.

Conclusions

Are the conclusions adequately supported by the data shown? Choose an item.

Reporting Standards

Does the manuscript adhere to the journal's guidelines on minimum standards of reporting? Choose an item.

Choose an item.

Statistics

Are you able to assess all statistics in the manuscript, including the appropriateness of statistical tests used? Choose an item.

Quality of Written English

Please indicate the quality of language in the manuscript: Choose an item.

Declaration of Competing Interests

Please complete a declaration of competing interests, considering the following questions:

- Have you in the past five years received reimbursements, fees, funding, or salary from an organisation that may in any way gain or lose financially from the publication of this manuscript, either now or in the future?
- Do you hold any stocks or shares in an organisation that may in any way gain or lose financially from the publication of this manuscript, either now or in the future?
- Do you hold or are you currently applying for any patents relating to the content of the manuscript?
- Have you received reimbursements, fees, funding, or salary from an organization that holds or has applied for patents relating to the content of the manuscript?
- Do you have any other financial competing interests?
- Do you have any non-financial competing interests in relation to this paper?

If you can answer no to all of the above, write 'I declare that I have no competing interests' below. If your reply is yes to any, please give details below.

I declare that I have no competing interests

I agree to the open peer review policy of the journal. I understand that my name will be included on my report to the authors and, if the manuscript is accepted for publication, my named report including any attachments I upload will be posted on the website along with the authors' responses. I agree for my report to be made available under an Open Access Creative Commons CC-BY license (http://creativecommons.org/licenses/by/4.0/). I understand that any comments which I do not wish to be included in my named report can be included as confidential comments to the editors, which will not be published.

Choose an item.

To further support our reviewers, we have joined with Publons, where you can gain additional credit to further highlight your hard work (see: https://publons.com/journal/530/gigascience). On publication of this paper, your review will be automatically added to Publons, you can then choose whether or not to claim your Publons credit. I understand this statement.

Yes Choose an item.
